# Urea-Based [2]Rotaxanes as Effective Phase-Transfer Organocatalysts: Hydrogen-Bonding Cooperative Activation Enabled by the Mechanical Bond

**DOI:** 10.1021/jacs.4c06630

**Published:** 2024-07-08

**Authors:** Julio Puigcerver, Jose M. Zamora-Gallego, Marta Marin-Luna, Alberto Martinez-Cuezva, Jose Berna

**Affiliations:** † Departamento de Quimica Organica, Facultad de Quimica, Regional Campus of International Excellence “Campus Mare Nostrum”, Universidad de Murcia, E-30100 Murcia, Spain

## Abstract

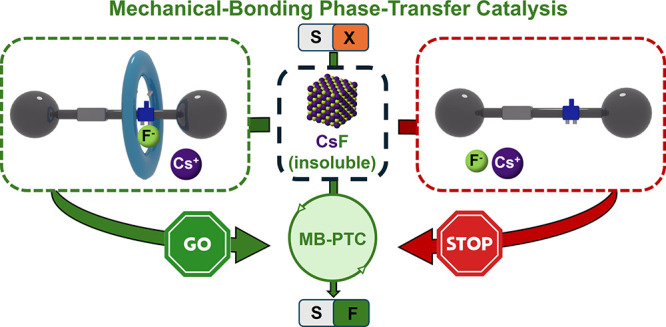

We finely designed a set of [2]rotaxanes with urea threads and tested them as hydrogen-bonding phase-transfer catalysts in two different nucleophilic substitutions requiring the activation of the reactant fluoride anion. The [2]rotaxane bearing a fluorinated macrocycle and a fluorine-containing urea thread displayed significantly enhanced catalytic activity in comparison with the combination of both noninterlocked components. This fact highlights the notably beneficial role of the mechanical bond, cooperatively activating the processes through hydrogen-bonding interactions.

Mechanically interlocked molecules (MIMs),^1^ specially [2]rotaxanes, have emerged as promising ligands in metal-mediated catalysis and as organocatalysts.^2^ The unique orthogonal entwining of the two components enables tailored environments around the catalytic active sites.^3^ Additionally, the stabilizing effect of the macrocycle when placed over different functional groups at the thread,^4^ coupled with the relative movement of the two components, makes rotaxanes ideal candidates for designing switchable catalysts^5^ by facilitating both activation or deactivation of catalytic sites (ON/OFF) or the selection of different activation modes.^6^ Recent investigations have demonstrated that organocatalysts embedded in [2]rotaxane architectures with benzylic amide-based macrocycles show no decrease in their catalytic activity, but instead, the mechanical bond enhances the efficiency of the interlocked catalyst.^7^

Hydrogen-bonding catalysis is a prevalent activation mode in homogeneous organocatalysis where small molecules with hydrogen bond donating groups, like diols,^8^ (thio)ureas,^9^ squaramides,^10^ or guanidinium ions, are employed.^11^ This activation mode has also been recently incorporated into a few examples of rotaxanes acting as interlocked catalysts.^12^

Another well-known feature of hydrogen bond donors is their ability to interact with anions, thereby facilitating tasks such as recognition or anion-binding.^13^ Taking advantage of this property, Gouverneur and co-workers have recently reported an asymmetric fluorination process under hydrogen-bonding phase-transfer catalysis (HB-PTC) using inorganic CsF as the nucleophilic fluoride source (Figure 1a).^14^ Their initial studies with monourea derivatives as catalysts indicated the convenience of activating the urea function with fluorine-containing *N*-aryl groups to satisfactorily catalyze the process, whereas nonactivated ureas were found to be inactive because of their disability of transporting insoluble CsF into the organic solution. Inspired by that work, we designed a series of hydrogen-bonded rotaxanes **2** featuring a urea group at the thread serving as the hydrogen bond donor catalytic site. Additionally, we functionalized the isophthalamide fragments of the macrocycle with fluorine atoms in order to increase the acidity of its amide NH groups. This type of polyamide rings are well known to selectively recognize anions, often in a volume-selective manner, on the basis of their cavity size.^15^ Consequently, our designed systems incorporate two components, a macrocycle and thread, that could desirably shape an optimal environment for a cooperative interaction with the small fluoride anion, as shown in Figure 1b. As a result, the catalytic activity of the putative rotaxanes **2** under HB-PTC might be enhanced in comparison with the noninterlocked threads **1**, and we hopefully expect a notable acceleration with the rotaxanes bearing activated fluorine-containing macrocycles.

**Figure 1:**
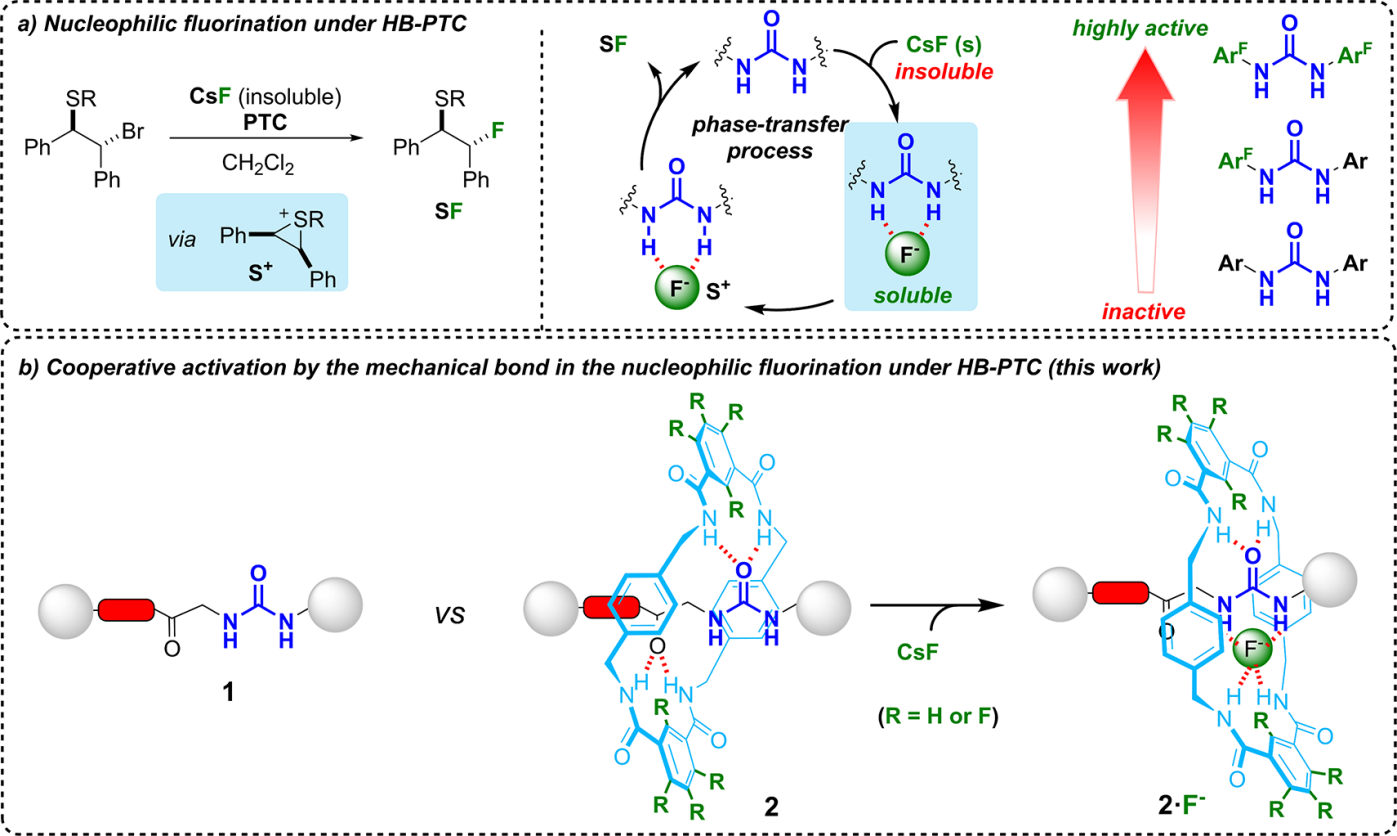
(a) CsF nucleophilic fluorination process under hydrogen-bonding phase-transfer catalysis (HB-PTC).^14^ (b) Design of interlocked urea-based organocatalysts for HB-PTC with a cooperative activation by the mechanical bond (this work).

For the synthesis of Leigh-type [2]rotaxanes, a suitable template on the thread is essential to facilitate the assembling of an entwined polyamide macrocycle via a five-component reaction with *p*-xylylenediamine and an isophthloyl dichloride.^16^ We selected the glycylglycine (GlyGly) as binding site, which was previously employed for this goal in hydrogen-bonded rotaxane synthesis (Scheme 1).^17^ Reaction of the GlyGly-containing derivative **3**(18) with 2,2-diphenylethyl isocyanate or 3,5-bis(trifluoromethyl)phenyl isocyanate yielded the urea-based threads **1a** and **1b**, respectively (see the Supporting Information for full synthetic procedures). Rotaxanes **2** were formed in reasonable yields by subjecting threads **1** to the standard conditions for hydrogen-bonded rotaxane formation using isophthaloyl chloride or perfluoroisophthaloyl dichloride, which has never been employed for this goal.^19^ The presence of fluorine atoms on the thread and macrocycle increases the acidity of the NH groups at both components of the rotaxanes. Computational calculations on the acidity of the amide groups within the macrocyclic rings were conducted using simplified models, which revealed a lower p*K*_a_ for the amide groups in rotaxane **2c** (p*K*_a_ = 10.6) compared with rotaxane **2b** (p*K*_a_ = 15.0) (see Scheme S4).^20^ The diverse modifications at both threads and macrocycles, with the presence or absence of activating fluorine atoms, enable us to compare the catalytic capability of these systems and determine if, as we initially envisioned, the mechanical bond can cooperatively activate phase-transfer catalysis.

**Scheme 1:**
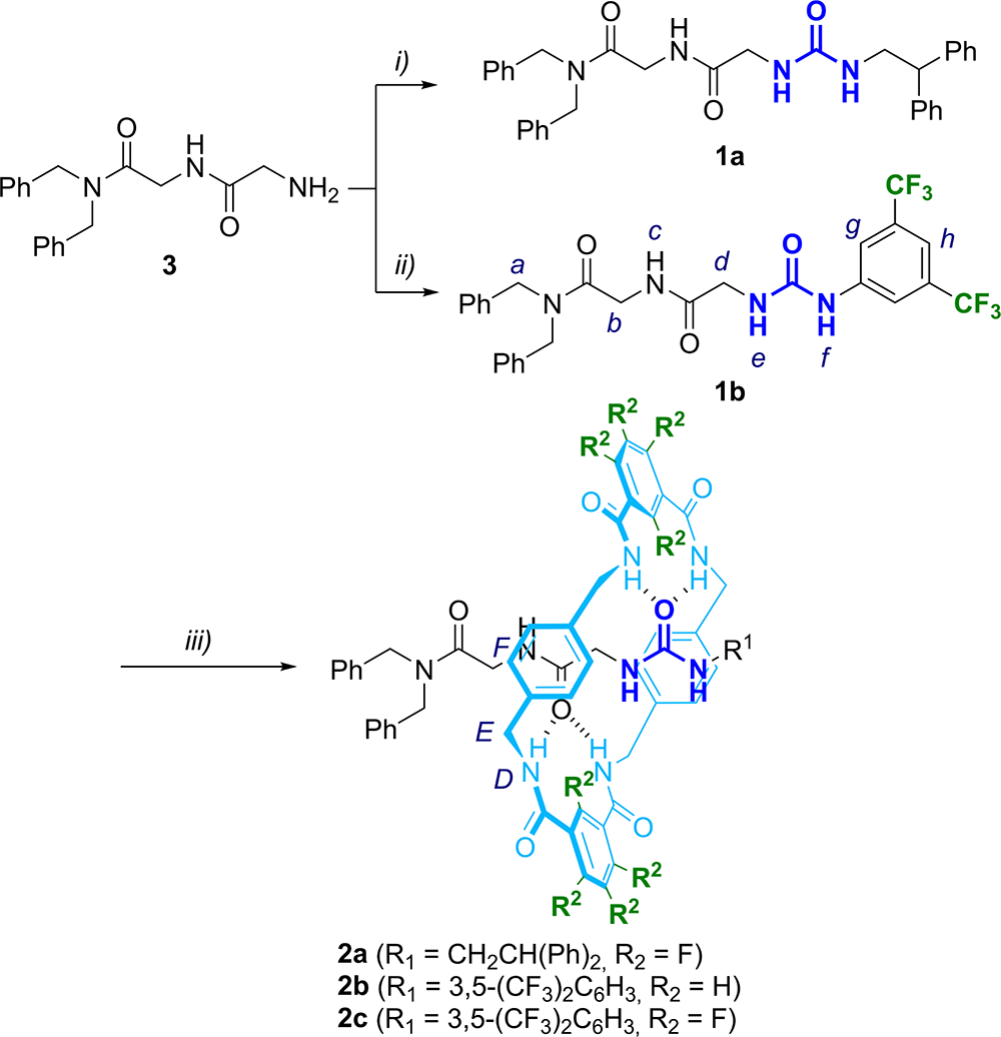
Synthesis of the Interlocked Systems **2** from Threads **1** Reaction conditions: (i) 2,2-diphenylethyl isocyanate, CH_2_Cl_2_, 0 °C, 41%; (ii) 3,5-bis(trifluoromethyl)phenyl isocyanate, THF, 25 °C, 54%; (iii) *p*-xylylenediamine (8 equiv), isophthaloyl dichloride or perfluoroisophthaloyl dichloride (8 equiv), Et_3_N (24 equiv), CHCl_3_, 25 °C, 4 h, 10% for **2a**, 6% for **2b**, 10% for **2c**.

It is known that ureas (**U**) similar to our threads **1** dimerize in solution.^21^ Indeed, we observed a concentration-dependent homodimerization process of the linear urea-based thread **1b** in solution (also at the solid state, see the Supporting Information for the single-crystal X-ray diffraction data of **1b**, Figure S1) with a calculated constant of 3.6 × 10^3^ M^–1^ (Figures S3 and S4). In contrast, similar dimerization processes were negligible for rotaxanes **2c** (*k*_dim_ = 63 M^–1^) and **2b** (*k*_dim_ = ∼0 M^–1^; no changes were observed at the ^1^H NMR spectra when diluting) where the presence of the bulky macrocycles avoids that scenario (Figures S5–S7). Moreover, we investigated the formation of supramolecular complexes between thread **1b** or rotaxanes **2b**,**c** with the fluoride anion through titration experiments with tetra-*n*-butylammonium fluoride (TBAF), by ^1^H NMR and UV–vis spectroscopic monitoring. The ^1^H NMR spectra showed notable shifts of the urea protons (H_e_ and H_f_, as labeled in Scheme 1) upon the incremental addition of TBAF, thereby indicating strong hydrogen-bonding interactions with the fluoride anion. Moreover, signals corresponding to the NH amide protons at the macrocycles (H_D_) in rotaxanes **2** also underwent significant shifts, thereby suggesting the cooperative participation of these protons in complexing the fluoride anion (see Figures S8–21). Monourea-related systems predominantly formed **U**_**2**_**:F**^**–**^ (2:1) complexes upon interaction with fluoride anions.^13b^ Accordingly, the Job plot derived from titration data of thread **1b** clearly indicated the formation of the (2:1) complex **1b**_**2**_**:F**^**–**^ (Figures S14). UV–vis spectra data well fitted a 2:1 model using Bindfit software^22^ with association constants of *k*_11_ = 1.0 × 10^6^ M^–1^ and *k*_12_ = 1.1 × 10^5^ M^–1^ (±8% error, Figure S8). In contrast, rotaxanes **2**, in which the macrocycle imposes significant steric hindrance, were unable to form 2:1 complexes with fluoride anions and preferentially assembled 1:1 complexes. Consequently, titrations for **2b** and **2c** clearly indicated the formation of **2:F**^**–**^ (1:1) complexes (Figure S17 and S21), with UV–vis data well-fitted to a 1:1 model, thereby yielding association constants of *k*_assoc_ = 3.6 × 10^5^ M^–1^ (±13% error) for **2b** and *k*_assoc_ = 2.6 × 10^5^ M^–1^ (±12% error) for **2c** (Figures S10 and S11).

We next explored the catalytic activity of threads **1** and their respective rotaxanes **2** in the nucleophilic fluorination reaction of compounds **4** and **6** under HB-PTC with the aim to discern the impact of the mechanical bond on their respective performance (see optimization of the reaction conditions on Tables S1 and S2). By employing CsF as an insoluble inorganic fluoride source, no background reactions occurred (Table 1, entries 1 and 8).^23^ Threads **1a** and **1b** exhibited minimal activity in both reactions, which yielded low conversions of compounds **4** and **6** to the fluorinated products **5** and **7** (Table 1, entries 2 and 3; 9 and 10). Comparatively, the presence of the entwined fluorinated macrocycle in rotaxane **2a** marginally enhanced its catalytic activity compared with the free thread **1a** (Table 1, entries 2 and 4; 9 and 11). A similar trend was observed when comparing rotaxane **2b**, featuring a nonfluorinated macrocycle, with its parent thread **1b**, showing a slightly higher yield in the fluorinated derivative **5** by using as catalyst the interlocked species (Table 1, entries 3 and 5). Remarkably, rotaxane **2c** comprising a fluorinated urea thread and a fluorinated macrocycle (for a total of 14 fluorine atoms) emerged as the most effective catalyst for both nucleophilic fluorinations to yield nearly quantitative yields of products **5** and **7** (Table 1, entries 6 and 12). As we expected, the mechanical bond, which linked both components, is crucial for the best catalytic performance of these systems. For the sake of further confirming the special role of the mechanical bond, we used an equimolecular mixture of free thread **1b** and free fluorinated macrocycle (**Mac**) as the catalytic system, but such combination did not accelerate the nucleophilic fluorination (Table 1, entry 7).

**Table 1:**
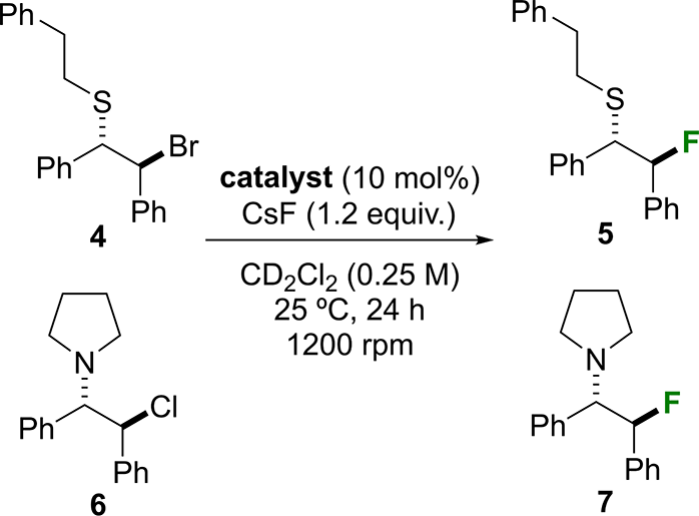
Evaluation of Threads **1** and Rotaxanes **2** in the HB-PTC with CsF^a^

entry	substrate	catalyst	**5** or **7** yield (%)^b^
1	**4**	none	0
2	**4**	**1a**	7
3	**4**	**1b**	8
4	**4**	**2a**	26
5	**4**	**2b**	30
6	**4**	**2c**	97
7	**4**	**1b+Mac**	9
8	**6**^c^	none	0
9	**6**^c^	**1a**	0
10	**6**^c^	**1b**	23
11	**6**^c^	**2a**	13
12	**6**^c^	**2c**	99

a Reaction conditions: **4** or **6** (0.025 mmol), CsF (1.2 equiv), CD_2_Cl_2_ (0.1 mL), 25 °C, 1200 rpm stirring; CsF used as provided by the supplier without any prior drying.

b Determined by ^19^F NMR using 4-fluoroanisole as internal standard.

c Reactions carried out with 5 mol % of catalyst for 10 h.

Having in mind that both threads **1b** and rotaxanes **2b,c** similarly complex the fluoride anion present in solution (see titration data with TBAF in the Supporting Information), the enhanced catalytic activity showed by rotaxane **2c** is mainly attributed to its capability to facilitate the transfer of the fluoride anion from solid CsF (insoluble in dichloromethane) into the solution. As we initially hypothesized, the fluorinated macrocycle in **2c** featuring NH groups of high acidity likely participates in intramolecular hydrogen bonding with the oxygen of the urea moiety.^24^ This intramolecular interaction in **2c** is evident at the solid state (Figure 2a) where one isophthalamide unit within the ring forms two bifurcated hydrogen bonds with the oxygen of the urea function. This cooperative interaction should enhance the affinity of the urea function toward the fluoride anion. Additionally, upon interaction with the fluoride anion, the second isophthalamide unit is available to establish additional hydrogen bonds with it.

**Figure 2:**
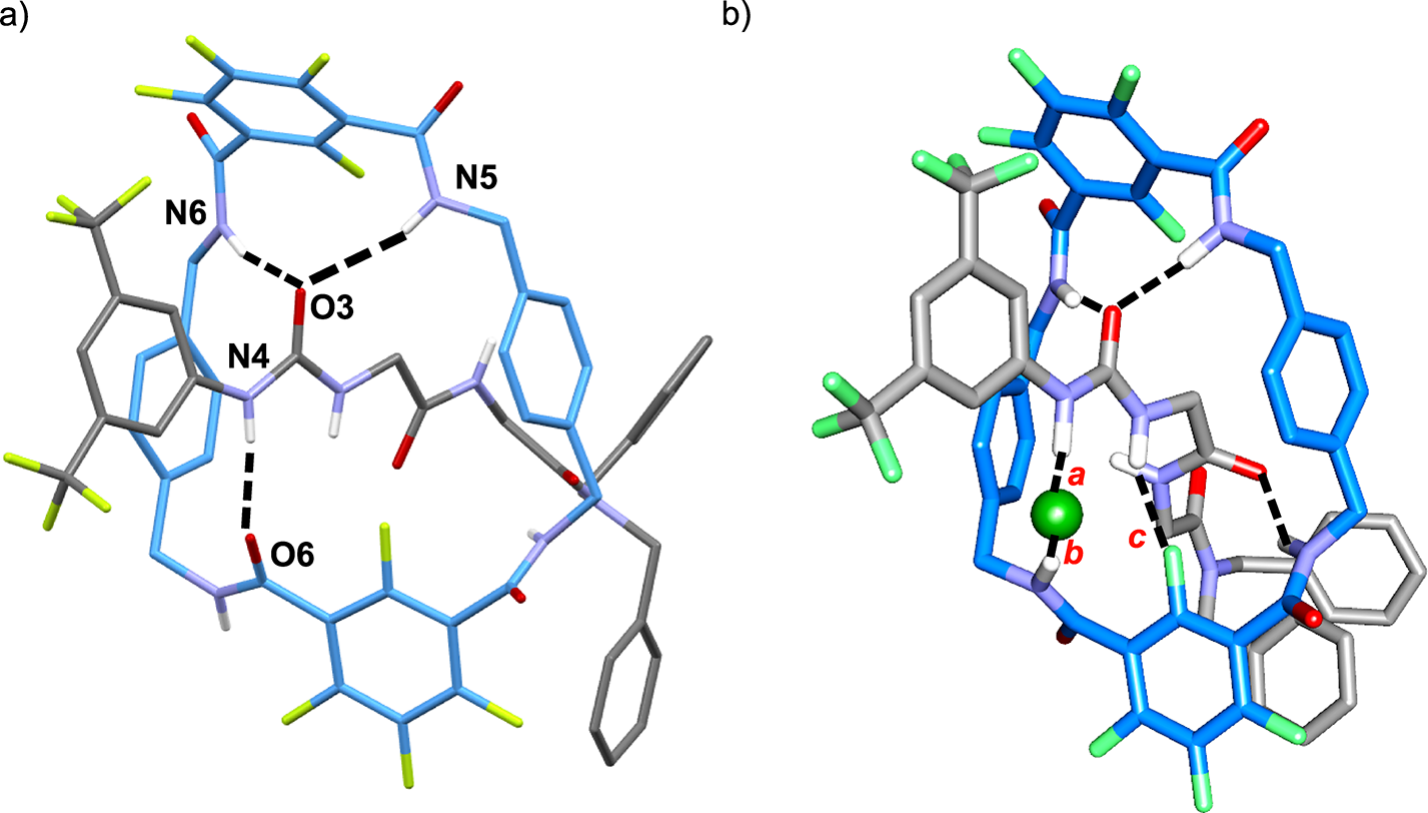
(a) X-ray structure of rotaxane **2c**. Intramolecular hydrogen bond lengths [Å] (and angles [°]): N5–H05···O3, 2.56 (163.4); N6–H06···O3, 2.20 (166.3); and N4–H04···O6, 2.02 (175). For clarity, selected hydrogens and solvent molecules have been deleted. (b) Computed structure of the **2c:F^–^** complex displaying the fluoride–rotaxane interactions and selected distances: *a* = 1.358; *b* = 1.498; and *c* = 2.146 Å.

In solution, analysis of the ^1^H–^1^H NOESY spectrum of rotaxane **2c** in the presence of 1 equiv of TBAF reveals intense cross peaks between some signals of the macrocycle (H_F_) with others of the bis(trifluoromethyl)phenyl stopper (H_g_), thereby indicating their spatial proximity once the 1:1 complex is formed (see Scheme 1 for lettering, Figures S22 and S23). This proximity is also observable in the ^1^H–^19^F HOESY spectrum from which we find crosspeaks between the fluorine atoms at the macrocycle and the H_g_ proton of the stopper (see Scheme 1 for lettering, Figures S24–27). Upon addition of increasing amount of TBAF, the macrocycle tends to be closer to the urea moiety for which we observe a deshielding of the signal attributed to the H_b_ of the methylene group at the thread (see Scheme 1 for lettering, Figure S19). Computational simulations also revealed that the optimized structure of the **2c:F**^**–**^ (1:1) complex shows a cooperative bidentate binding mode in which **2c** holds the fluoride atom involving the most acidic NH of the urea system (dF^–^···H = 1.358 Å, distance *a* in Figure 2b) and one NH of the isophthalamide moiety (dF^–^···H = 1.498 Å, distance *b* in Figure 2b). Besides, one fluorine atom at the ortho position of one isophthalamide ring is directly interacting with the second NH of the urea fragment (dF···H = 2.146 Å, distance *c* in Figure 2b), thus further enhancing the stability of the complex. Calculations also predict that the complexation energy of **2c:F**^**–**^ is higher than those of the other fluoride complexes tested (those with thread **1b** and rotaxane **2b**), which supports the conclusion that the capability of catalyst **2c** to induce the phase transfer of the fluoride anion is the highest of the herein designed catalysts (see the Supporting Information).

In summary, we successfully synthesized a series of hydrogen-bonded interlocked urea derivatives and evaluated their efficacy as HB-PTC organocatalysts in two fluorination processes by using CsF as the nonsoluble nucleophilic fluoride source, and their reactivity was compared with their noninterlocked counterparts. As presumed, when the isophthalamide units of the macrocycle were substituted with electron-withdrawing fluorine atoms, the resulting rotaxane exhibited a spectacular improvement in its catalytic activity. These findings underscore the stark influence of the mechanical bond on the catalytic performance of these systems, which cooperatively activates the process by intercomponent hydrogen-bonding. Indeed, in the absence of the mechanical bond—more specifically by using the two segregated components of the rotaxane, the noninterlocked thread and macrocycle, as the catalytic system—the reaction did not occur. Our ongoing research aims to further explore the design of novel mechanical bonding phase-transfer catalysts, including their asymmetric variants, with the goal of enhancing the utility and versatility of mechanically interlocked catalysts.

## Supporting Information Available

Supplemental experimental procedures, Figures S1–S30, Tables S1–S7, Cartesian coordinates of the computed structures and supplemental references (PDF)
