# Supplementary material for: Urea-Based [2]Rotaxanes as Effective Phase-Transfer Organocatalysts: Hydrogen-Bonding Cooperative Activation Enabled by the Mechanical Bond
Source: J Am Chem Soc. 2024 Jul 8;146(33):22887–92. doi: 10.1021/jacs.4c06630 (PMC11345763; doi:10.1021/jacs.4c06630)
Supplement: Supplementary file 1 — ja4c06630_si_001.pdf [file ja4c06630_si_001.pdf]

SUPPORTING INFORMATION

Urea-based [2]Rotaxanes as Effective Phase-Transfer Organocatalysts: Hydrogen-Bonding Cooperative Activation Enabled by the Mechanical Bond

Julio Puigcerver,[a] Jose M. Zamora-Gallego,[a] Marta Marin-Luna,[a] Alberto Martinez-Cuezva,*[a] Jose Berna*[a]

[a]Departamento de Química Orgánica, Facultad de Química, Regional Campus of International Excellence "Campus Mare Nostrum", Universidad de Murcia, E-30100 Murcia, Spain.

Email: amcuezva@um.es; ppberna@um.es

Table of Contents ………………………………………………………………………………

1. General experimental section………………………………………………………… **S1**

2. Synthesis of the catalysts ……………………………………………………………… **S2**

2.1. Synthesis of urea threads 1 ………………………………………………………… **S2**

2.2. Synthesis of urea rotaxanes 2 ……………………………………………………… **S4**

2.3. Synthesis of macrocycle (Mac) ……………………………………………………… **S8**

3. Synthesis of the substrates and identification of the products ……………………… **S9**

4. Phase-transfer nucleophilic fluorinations ……………………………………………… **S11**

5. Estimation of the pKa for the deprotonation of model amides ……………………… **S13**

6. Crystal data and structure refinements ……………………………………………… **S14**

7. Variable concentration studies ………………………………………………………… **S18**

8. Fluoride binding studies ……………………………………………………………… **S23**

8.1. UV titrations ……………………………………………………………………… **S23**

8.2. NMR titrations …………………………………………………………………… **S26**

9. NMR study of the position of the macrocycle in the rotaxane complex 2c·F^- ………… **S33**

10. Computational studies ………………………………………………………………… **S37**

11. ^1H, ^{13}C and ^{19}F NMR spectra of synthesized compounds ……………………… **S52**

12. References……………………………………………………………………………… **S78**

1. General Experimental Section

Unless stated otherwise, all reagents were purchased from Aldrich Chemicals and used without further purification. HPLC grade solvents (Scharlab) were nitrogen saturated and were dried and deoxygenated using an Innovative Technology Inc. Pure-Solv 400 Solvent Purification System. Column chromatography was carried out using silica gel (60 Å, 70-200 µm, SDS) as stationary phase, and TLC was performed on precoated silica gel on aluminium cards (0.25 mm thick, with fluorescent indicator 254 nm, Fluka) and observed under UV light. Size exclusion chromatography (SEC) was carried out using TOYOPEARL® size exclusion media (HW-40S, 20 – 40 µm). All melting points were determined on a Kofler hot-plate melting point apparatus and are uncorrected. ^{1}H- and ^{13}C-NMR spectra were recorded on a Bruker Avance 300, 400 and 600 MHz instruments. ^{1}H NMR chemical shifts are reported relative to Me_4Si and were referenced via residual proton resonances of the corresponding deuterated solvent, whereas ^{13}C NMR spectra are reported relative to Me_4Si using the carbon signals of the deuterated solvent. Signals in the ^{1}H and ^{13}C NMR spectra of the synthesized compounds were assigned with the aid of DEPT, APT, or two-dimensional NMR experiments (COSY, HMQC and HMBC). Abbreviations of coupling patterns are as follows: br, broad; s, singlet; d, doublet; t, triplet; q, quadruplet; qui, quintuplet; m, multiplet. The deuterated solvent $CDCl_3$ was dried over $CaCl_2$ and stored with molecular sieves prior to use. Coupling constants (J) are expressed in Hz. High-resolution mass spectra (HRMS) were obtained using a time-of-flight (TOF) instrument equipped with electrospray ionization (ESI).

Abbreviation list:

HOBt: hydroxybenzotriazole

DIPEA: *N*,*N*-diisopropylethylamine

EDCI·HCl: *N*-(3-dimethylaminopropyl)-*N*’-ethylcarbodiimide hydrochloride

TFA: trifluoroacetic acid

THF: tetrahydrofurane

DCM: dichloromethane

DMSO: dimethyl sulfoxide

DMF: dimethylformamide

PTFE: polytetrafluoroethylene

2. Synthesis of the catalysts

2.1. Synthesis of urea threads 1

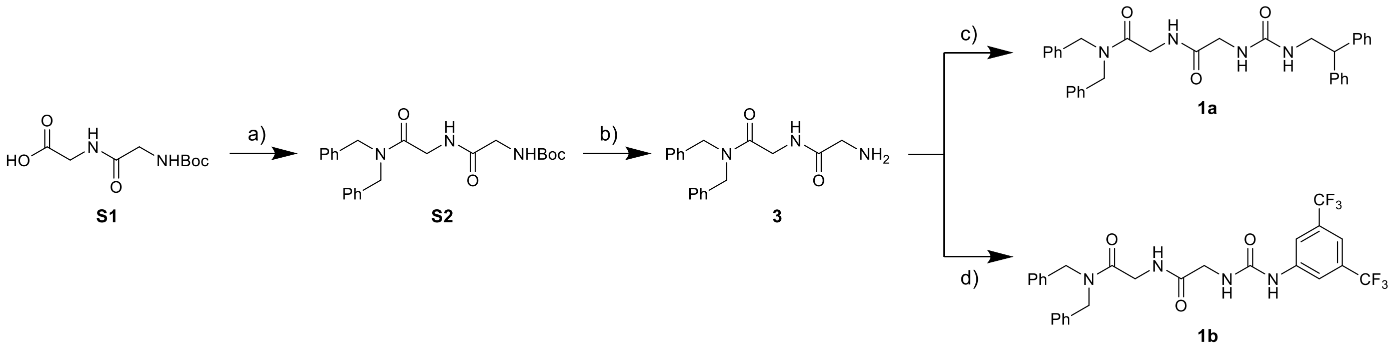

Scheme S1. *Reactions conditions*: a) Dibenzylamine, HOBt, DiPEA, EDCI·HCl, CH_2Cl_2, 0 °C to 25 °C, overnight; b) TFA, $CHCl_3$, 0 °C to 25 °C, overnight; c) 2,2-Diphenylethyl isocyanate, triethylamine, CH_2Cl_2, 25 °C, overnight; d) 3,5-*bis*(trifluoromethyl)phenyl isocyanate, THF, 0 ºC to 25 ºC, overnight.

Compound S2

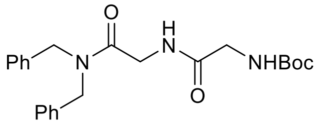

S2

Fragment **S2** was synthesized following a described procedure previously reported in literature and showed identical spectroscopic data as those reported therein.[1]

Compound 3

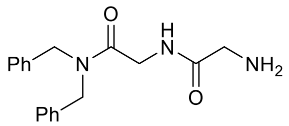

3

Fragment **3** was synthesized following a described procedure previously reported in literature and showed identical spectroscopic data as those reported therein.[1]

Thread 1a

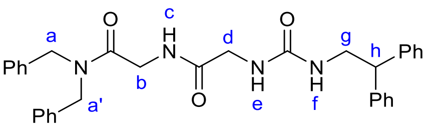

1a

To a stirred solution of triphosgene (1.63 g, 5.5 mmol) in dry toluene (30 mL) was added 2,2-diphenylethylamine (2.17 g, 11.0 mmol) and the mixture was refluxed for 4 h. After this time, the solvent was removed under reduced pressure to obtain the 2,2-diphenylethyl isocyanate as an orange oil. That compound was solved in DCM (20 mL) and was added dropwise to a cooled (0 ºC) solution of amine **3**

(3.6 g, 11.6 mmol) and triethylamine (1.53 mL, 11.0 mmol) in DCM (60 mL). The mixture was stirred at room temperature overnight. Then, the mixture was washed with NaOH 1M (3 x 30 mL) and the aqueous phase was extracted again with DCM (2 x 20 mL). The organic extracts were combined, dried over anhydrous $MgSO_4$, filtered, and concentrated to dryness. The resulting crude was subjected to column chromatography on silica gel using $CHCl_3$/acetone (4:1 to 1:1) as eluent to give the title product as a white solid (2.42 g, 41%); mp 169-171 °C; **^{1}H-NMR (400 MHz, $CDCl_3$, 298 K) δ**: 7.42 – 7.07 (m, 21H, H_{Ph+c}), 5.31 (t, *J* = 5.4 Hz, 1H, H_e), 4.91 (t, *J* = 5.3 Hz, 1H, H_f), 4.54 (s, 2H, $H_{a'}$), 4.37 (s, 2H, H_a), 4.17 (t, *J* = 7.9 Hz, 1H, H_h), 4.08 (d, *J* = 4.3 Hz, 2H, H_b), 3.83 – 3.78 (m, 4H, H_{d+g}) ppm; **^{1}H-NMR (400 MHz, DMSO-d_6, 298 K) δ:** 7.95 (t, *J* = 5.4 Hz, 1H, H_c), 7.41 – 7.13 (m, 20H, H_{Ph}), 6.20 (t, *J* = 5.5 Hz, 1H, H_e), 6.11 (t, *J* = 5.7 Hz, 1H, H_f), 4.53 (s, 2H, $H_{a'}$), 4.50 (s, 2H, H_a), 4.11 (t, *J* = 7.8 Hz, 1H, H_h), 4.03 (d, *J* = 5.3 Hz, 2H, H_b), 3.69 – 3.63 (m, 4H, H_{d+g}) ppm; **^{13}C-NMR (100 MHz, $CDCl_3$, 298 K) δ**: 170.5 (CO), 168.7 (CO), 158.0 (CO), 142.2 (C), 136.4 (C), 135.2 (C), 129.3 (CH), 128.9 (CH), 128.8 (CH), 128.3 (CH), 128.3 (CH), 128.2 (CH), 127.9 (CH), 126.8 (CH), 126.6 (CH), 51.3 (CH), 49.2 (CH_2), 48.8 (CH_2), 45.0 (CH_2), 43.9 (CH_2), 41.4 (CH_2) ppm; **HRMS (ESI)**: calcd for $C_{33}H_{33}N_4O_3$ [M-H]$^-$ 533.2558, found 533.2555; **IR (neat) ν:** 3281.3, 1641.1, 1629.6, 1561.1, 1450.2, 695.2, 626.8, 534.2 cm^{-1}.

Thread 1b

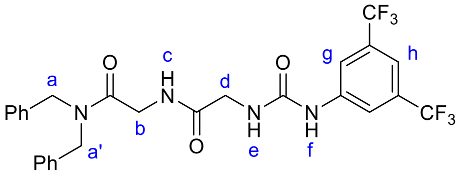

1b

To a cooled at 0 °C solution of amine **3** (3.6 g, 11.6 mmol) in dry THF (70 mL) was added dropwise a solution of 2,2-*bis*(trifluoromethyl)phenyl isocyanate (1.81 mL, 10.6 mmol) in dry THF (10 mL). The mixture was stirred at room temperature overnight. After this time, the solvent was removed under reduced pressure and the crude was redissolved in DCM (60 mL). The solution was washed with NaOH 1M (3 x 30 mL) and the aqueous phase was extracted again with DCM (2 x 20 mL). The organic extracts were combined, dried over anhydrous $MgSO_4$, filtered, and concentrated to dryness. The resulting crude was subjected to column chromatography on silica gel using $CHCl_3$/acetone (4:1 to 1:1) as eluent to give the title product as a white solid (3.26 g, 54%); mp 220-222 °C; **^{1}H-NMR (400 MHz, $CDCl_3$, 298 K) δ**: 8.20 (s, 1H, H_f), 7.71 (s, 2H, H_g), 7.40 – 7.26 (m, 8H, H_{Ph+h}), 7.19 – 7.11 (m, 4H, H_{Ph+c}), 6.40 (s, 1H, H_e), 4.61 (s, 2H, $H_{a'}$), 4.44 (s, 2H, H_a), 4.22 (d, *J* = 4.6 Hz, 2H, H_b), 4.02 (d, *J* = 5.8 Hz, 2H, H_d) ppm; **^{1}H-NMR (400 MHz, DMSO-d_6, 298 K) δ:** 9.55 (s, 1H, H_f), 8.19 (t, *J* = 5.5 Hz, 1H, H_c), 8.07 (s, 2H, H_g), 7.56 (s, 1H, H_h), 7.42 – 7.19 (m, 10H, H_{Ph}), 6.70 (t, *J* = 5.5 Hz, 1H, H_e), 4.54 (s, 2H, $H_{a'}$), 4.50 (s, 2H, H_a), 4.08 (d, *J* = 5.4 Hz, 2H, H_b), 3.80 (d, *J* = 5.4 Hz, 2H, H_d) ppm; **^{13}C-NMR (100 MHz, DMSO-d_6, 298 K) δ**: 169.4 (CO), 168.9 (CO), 154.8 (CO), 142.4 (C), 137.4 (C), 136.8 (C), 130.7 (q, *J* = 32.4 Hz, *C*-CF_3),

128.8 (CH), 128.4 (CH), 127.7 (CH), 127.4 (CH), 127.1 (CH), 126.6 (CH), 123.4 (q, J = 273.0 Hz, CF_3), 117.2 (CH), 113.6 (CH), 49.2 (CH_2), 48.5 (CH_2), 42.5 (CH_2), 40.6 (CH_2) ppm; **^{19}F-NMR (376 MHz, DMSO-d6, 298 K) δ:** -61.73 (s, 6F, CF_3) ppm; **HRMS (ESI)**: calcd for $C_{27}H_{25}F_6N_4O_3$ $[M+H]^+$ 567.1825, found 567.1826; **IR (neat) ν:** 3403.7, 3333.4, 3290.0, 1661.4, 1636.3, 1527.4, 1386.6, 1273.8, 1166.7, 1134.9, 692.3 cm^{-1}.

2.2. Synthesis of urea rotaxanes 2

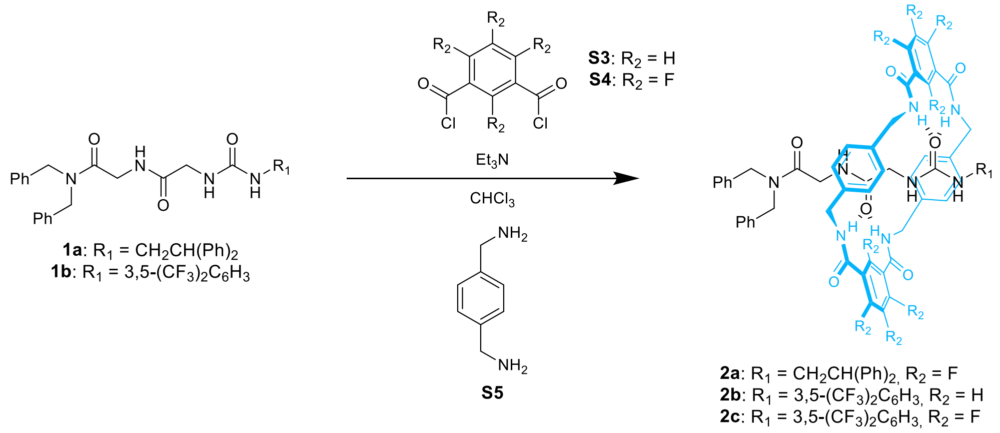

Scheme S2. *Reactions conditions*: Acid dichloride, *p*-xylylenediamine, triethylamine, $CHCl_3$, 25 ºC.

Thread (1 equiv.) and Et_3N (24 equiv.) in anhydrous $CHCl_3$ (500 mL/mmol of thread) were stirred vigorously whilst solutions of *p*-xylylenediamine (**S5**) (8 equiv.) in anhydrous $CHCl_3$ (20 mL) and the corresponding acid dichloride (8 equiv.) in anhydrous $CHCl_3$ (20 mL) were simultaneously added (0.06 mL/min) for 5-6 h using motor-driven syringe pumps. After a further 4 h, the resulting suspension was filtered through a Celite® pad and the solvent was removed under reduced pressure. The resulting solid was subjected to column chromatography (silica gel) and/or size exclusion chromatography to yield unconsumed thread and the corresponding rotaxane.

Perfluoroisophthaloyl dichloride (S4)

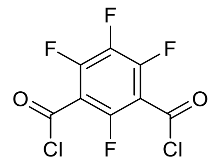

S4

To a stirred suspension of perfluoroisophthalic acid (4.99 g, 20.9 mmol) and a drop of DMF in anhydrous DCM (40 mL) was carefully added oxalyl chloride (7.29 mL, 83.8 mmol). The mixture was refluxed during 3-4 h until a yellow solution was obtained. After that, the solvent was removed several times under reduced pressure to obtain the title product **S4** as a yellow oil, which was used directly in the rotaxane formation.

Rotaxane 2a

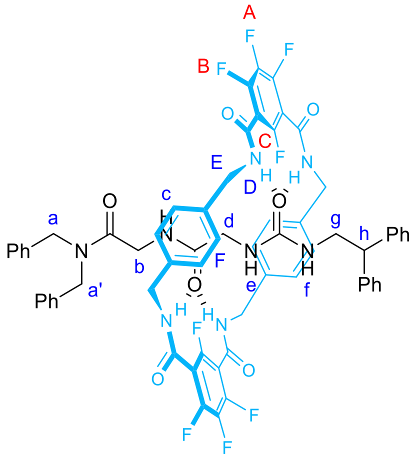

2a

Rotaxane **2a** was obtained following the described method from thread **1a** (1.40 g, 2.62 mmol), triethylamine (8.75 mL, 62.9 mmol), *p*-xylylenediamine (**S5**) (2.91 g, 20.9 mmol) and perfluoroisophthaloyl dichloride (**S4**) (5.75 g, 20.9 mmol). The resulting crude was purified by column chromatography (silica gel) using $CHCl_3$/Acetone (2:1) as eluent to give the title product as a white solid (0.32 g, 10%); mp 132 – 134 °C; **^{1}H-NMR (400 MHz, $CDCl_3$, 298 K) δ**: 7.45 – 7.25 (m, 14H, H_{D+Ph}), 7.25 – 7.16 (m, 2H, H_{Ph}), 7.16 – 7.04 (m, 8H, H_{Ph}), 7.00 (s, 8H, H_F), 6.18 (t, *J* = 4.5 Hz, 1H, H_c), 4.52 (dd, *J* = 14.5, 5.8 Hz, 4H, H_E), 4.46 (s, 2H, H_a), 4.37 (s, 2H, $H_{a'}$), 4.29 (dd, *J* = 14.5, 4.5 Hz, 4H, $H_{E'}$), 4.15 (t, *J* = 5.5 Hz, 1H, H_f), 3.97 (t, *J* = 5.5 Hz, 1H, H_e), 3.81 (t, *J* = 8.1 Hz, 1H, H_h), 3.59 (d, *J* = 4.4 Hz, 2H, H_b), 3.30 (dd, *J* = 7.8, 5.6 Hz, 2H, H_g), 2.87 (d, *J* = 5.5 Hz, 2H, H_d) ppm; **^{1}H-NMR (400 MHz, DMSO-d_6, 298 K) δ:** 9.10 (t, *J* = 4.9 Hz, 4H, H_D), 7.46 – 7.07 (m, 20H, H_{Ph}), 7.06 (s, 8H, H_F), 6.20 (bs, 1H, H_c), 5.51 (t, *J* = 5.6 Hz, 1H, H_f), 5.36 (t, *J* = 6.2 Hz, 1H, H_e), 4.49 – 4.23 (m, 10H, H_{E+a}), 4.17 (s, 2H, $H_{a'}$), 3.98 (t, *J* = 7.8 Hz, 1H, H_h), 3.13 (d, *J* = 3.2 Hz, 2H, H_b), 2.45 (d, *J* = 5.7 Hz, 2H, H_d) ppm (signal related with protons H_g is overlapped under intense water signal); **^{13}C-NMR (100 MHz, $CDCl_3$, 298 K) δ**: 170.4 (CO), 168.4 (CO), 158.1 (CO), 157.4 (CO), 141.9 (C), 136.7 (C), 136.1 (C), 134.9 (C), 129.5 (CH), 129.0 (CH), 129.0 (CH), 128.9 (CH), 128.6 (CH), 128.2 (CH), 128.0 (CH), 127.8 (CH), 127.1 (CH), 126.5 (CH), 50.9 (CH), 50.1 (CH_2), 49.7 (CH_2), 44.5 (CH_2), 44.4 (CH_2), 42.7 (CH_2), 41.4 (CH_2) ppm, (the signals from aromatic carbons of the macrocycle coupled with fluorine atoms could not be identified due to the strength and difficult multiplicity patterns of these couplings); **^{19}F-NMR (376 MHz, $CDCl_3$, 298 K) δ:** -115.57 (d, *J* = 11.0 Hz, 2F, F_C), -130.30 (d, *J* = 21.9 Hz, 4F, F_B), -161.58 (td, *J* = 22.2, 11.1 Hz, 2F, F_A) ppm; **HRMS (ESI)**: calcd for $C_{65}H_{55}F_8N_8O_7$ $[M+H]^+$ 1211.4060, found 1211.4066. **IR (neat) ν:** 3267.8, 1641.1, 1536.0, 1480.1, 1452.1, 1267.0, 1223.6, 697.1 cm^{-1}.

Rotaxane 2b

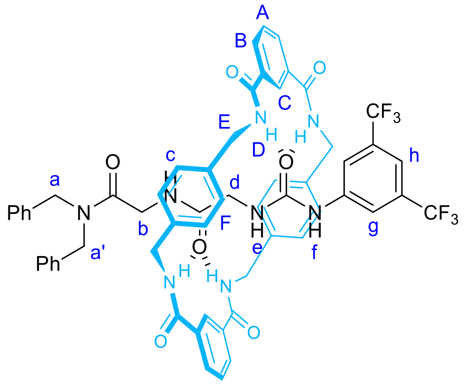

2b

Rotaxane **2b** was obtained following the described method from thread **1b** (0.80 g, 1.41 mmol), triethylamine (4.70 mL, 33.8 mmol), *p*-xylylenediamine (**S5**) (1.57 g, 11.3 mmol) and isophthaloyl chloride (**S3**) (2.29 g, 11.3 mmol). The resulting crude was purified by column chromatography (silica gel) using $CHCl_3$/Acetone (4:1 to 1:1) as eluent to give a fraction that contains unconsumed thread and the desired rotaxane. This fraction was subjected to a size exclusion chromatography using $CHCl_3$/MeOH (1:1) as eluent to give the title product as a white solid (92 mg, 6%); mp 252-254 °C; **^{1}H-NMR (400 MHz, $CDCl_3$, 323 K) δ**: 8.55 (s, 1H, H_f), 8.38 (s, 2H, H_C), 8.06 (dd, *J* = 7.8, 1.7 Hz, 4H, H_B), 7.64 (s, 2H, H_g), 7.52 (t, *J* = 7.7 Hz, 2H, H_A), 7.42 (s, 1H, H_h), 7.35 – 7.16 (m, 10H, H_{Ph+D}), 7.06 (s, 8H, H_F), 6.96 (d, *J* = 7.1 Hz, 2H, H_{Ph}), 6.70 (d, *J* = 7.5 Hz, 2H, H_{Ph}), 6.40 (s, 1H, H_c), 5.28 (t, *J* = 6.2 Hz, 1H, H_e), 4.46 (ddd, *J* = 78.0, 14.4, 5.5 Hz, 8H, H_E), 4.32 (s, 2H, $H_{a'}$), 4.03 (s, 2H, H_a), 3.45 (d, *J* = 6.0 Hz, 2H, H_d), 3.05 (d, *J* = 3.8 Hz, 2H, H_b) ppm; **^{1}H-NMR (400 MHz, DMSO-d_6, 298 K) δ**: 9.44 (s, 1H, H_f), 8.46 (s, 2H, H_C), 8.36 (t, *J* = 5.0 Hz, 4H, H_D), 8.00 (dd, *J* = 7.7, 1.6 Hz, 4H, H_B), 7.85 (s, 2H, H_g), 7.57 (t, *J* = 7.8 Hz, 2H, H_A), 7.52 (s, 1H, H_h), 7.48 (t, *J* = 4.9 Hz, 1H, H_c), 7.25 – 7.14 (m, 6H, H_{Ph}), 7.05 (s, 8H, H_F), 7.05 – 7.02 (m, 2H, H_{Ph}), 6.93 – 6.85 (m, 2H, H_{Ph}), 6.04 (t, *J* = 5.0 Hz, 1H, H_e), 4.45 – 4.19 (m, 12H, $H_{E+a+a'}$), 3.28 (d, *J* = 4.8 Hz, 2H, H_b), 2.99 (d, *J* = 5.0 Hz, 2H, H_d) ppm; **^{13}C-NMR (100 MHz, DMSO-d_6, 298 K) δ**: 169.5 (CO), 168.0 (CO), 165.8 (CO), 154.7 (CO), 141.7 (C), 136.9 (C), 136.2 (C), 134.1 (C), 130.6 (q, *J* = 32.7 Hz, *C*-CF_3), 130.4 (CH), 128.7 (CH), 128.6 (CH), 128.3 (CH), 127.9 (CH), 127.3 (CH), 127.2 (CH), 126.2 (CH), 125.6 (CH), 123.3 (q, *J* = 273.2 Hz, CF_3), 117.4 (CH), 114.0 (CH), 49.5 (CH_2), 49.3 (CH_2), 43.6 (CH_2), 41.9 (CH_2), 40.5 (CH_2) ppm; **^{19}F-NMR (376 MHz, $CDCl_3$, 298 K) δ:** -63.31 (s, 6F, CF_3) ppm; **HRMS (ESI)**: calcd for $C_{59}H_{53}F_6N_8O_7$ $[M+H]^+$ 1099.3936, found 1099.3925. **IR (neat) ν:** 3319.9, 1641.1, 1529.3, 1514.8, 1473.4, 1274.7, 1174.4, 1126.2, 699.1, 681.7 cm^{-1}.

Rotaxane 2c

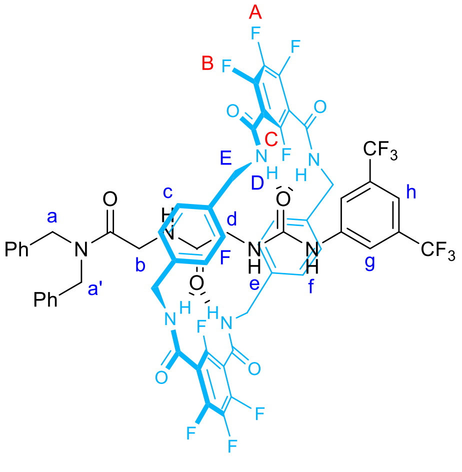

2c

Rotaxane **2c** was obtained following the described method from thread **1b** (1.00 g, 1.77 mmol), triethylamine (5.90 mL, 42.4 mmol), *p*-xylylenediamine (**S5**) (1.97 g, 14.1 mmol) and perfluoroisophthaloyl chloride (**S4**) (3.88 g, 14.1 mmol). The resulting crude was purified by column chromatography (silica gel) using $CHCl_3$/Acetone (4:1 to 2:1) as eluent to give a fraction that contains unconsumed thread and the desired rotaxane. This fraction was subjected to a size exclusion chromatography using $CHCl_3$/MeOH (1:1) as eluent to give the title product as a white solid (220 mg, 10%); mp 134-136 °C; **^{1}H-NMR (600 MHz, $CDCl_3$, 298 K) δ**: 7.78 (s, 1H, H_f), 7.66 (s, 2H, H_g), 7.50 – 7.33 (m, 11H, H_{Ph+h+D}), 7.15 – 7.10 (m, 4H, H_{Ph}), 7.04 (s, 8H, H_F), 6.29 (s, 1H, H_c), 4.97 (s, 1H, H_e), 4.58 – 4.49 (m, 6H, $H_{E'+a'}$), 4.45 – 4.32 (m, 6H, H_{E+a}), 3.59 (d, *J* = 4.6 Hz, 2H, H_b), 2.88 (d, *J* = 5.1 Hz, 2H, H_d) ppm; **^{1}H-NMR (400 MHz, DMSO-d$_6$, 298 K) δ**: 9.11 (s, 1H, H_f), 8.89 (t, *J* = 5.3 Hz, 4H, H_D), 7.93 (s, 2H, H_g), 7.60 (s, 1H, H_h), 7.47 – 7.22 (m, 6H, H_{Ph}), 7.19 – 7.08 (m, 4H, H_{Ph}), 7.14 (s, 8H, H_F), 6.23 (t, *J* = 5.6 Hz, 1H, H_e), 6.16 (bs, 1H, H_c), 4.51 – 4.27 (m, 10H, H_{E+a}), 4.19 (s, 2H, $H_{a'}$), 3.16 (d, *J* = 4.2 Hz, 2H, H_b), 2.69 (d, *J* = 5.8 Hz, 2H, H_d) ppm; **^{13}C-NMR (151 MHz, $CDCl_3$, 298 K) δ**: 169.6 (CO), 168.2 (CO), 158.5 (CO), 154.8 (CO), 140.9 (C), 136.6 (C), 136.5 (C), 135.3 (C), 132.2 (q, *J* = 32.5 Hz, *C*-CF_3), 129.5 (CH), 129.0 (CH), 128.6 (CH), 128.2 (CH), 128.1 (CH), 126.5 (CH), 123.3 (q, *J* = 272.4 Hz, CF_3), 117.5 (CH), 115.5 (CH), 50.1 (CH_2), 50.0 (CH_2), 44.5 (CH_2), 42.1 (CH_2), 41.5 (CH_2) ppm (the signals from aromatic carbons of the macrocycle coupled with fluorine atoms could not be identified due to the strength and difficult multiplicity patterns of these couplings); **^{19}F-NMR (376 MHz, $CDCl_3$, 298 K) δ:** -63.29 (s, 6F, C*F*$_3$), -115.34 (d, *J* = 8.6 Hz, 2F, F_C), -129.81 (d, *J* = 22.0 Hz, 4F, F_B), -161.23 (td, *J* = 22.1, 11.0 Hz, 2F, F_A) ppm; **HRMS (ESI)**: calcd for $C_{59}H_{45}F_{14}N_8O_7$ $[M+H]^+$ 1243.3182, found 1243.3195. **IR (neat) ν:** 3283.2, 2924.5, 1648.8, 1541.8, 1515.8, 1482.0, 1385.6, 1275.7, 1212.0, 1176.4, 1127.2, 1109.8, 700.0, 680.7 cm^{-1}.

2.3. Synthesis of macrocycle (Mac)

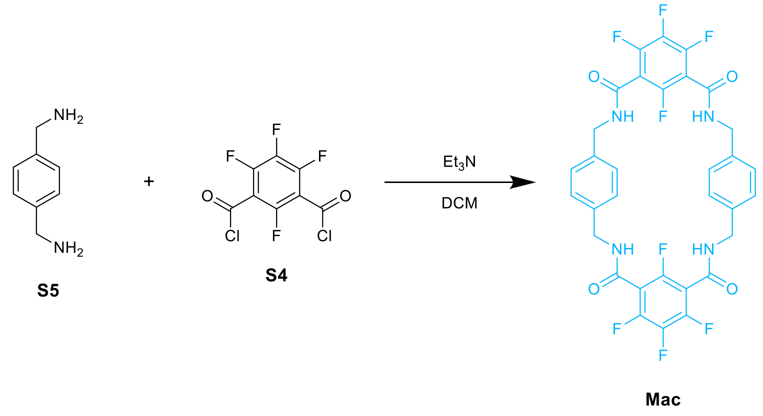

Scheme S3. *Reactions conditions*: perfluoroisophthaloyl dichloride, *p*-xylylenediamine, triethylamine, CH_2Cl_2, 25 ºC.

Macrocycle (Mac)

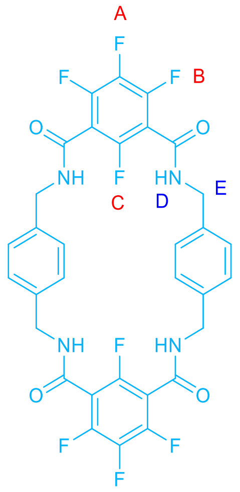

To a stirred solution of *p*-xylylenediamine (**S5**) (0.29 g, 2.1 mmol) and triethylamine (0.88 mL, 6.3 mmol) in anhydrous CH_2Cl_2 (700 mL) was slowly added (0.013 mL/min) a solution of perfluoroisophthaloyl dichloride (**S4**) (0.58 g, 2.1 mmol) in anhydrous CH_2Cl_2 (20 mL) during 24 h. After the addition, the mixture was stirred for another 24 h, then the resulting white suspension was filtered and the solid was washed several times with portions of CH_2Cl_2. The solid was recrystallized in DMF to isolate the title product as a white solid (192 mg, 27%); mp > 300 °C; **^{1}H-NMR (400 MHz, DMSO, 298 K) δ**: 9.51 (t, *J* = 6.1 Hz, 4H, H_D), 7.28 (s, 8H, H_F), 4.49 (d, *J* = 6.2 Hz, 8H, H_E) ppm; **^{13}C-NMR (100 MHz, DMSO, 298 K) δ**: 157.4 (CO), 137.2 (C), 126.8 (CH), 42.2 (CH_2) ppm, (the signals from aromatic carbons of the macrocycle coupled with fluorine atoms could not be identified due to the strength and difficult multiplicity patterns of these couplings); **^{19}F-NMR (376 MHz, DMSO, 298 K) δ:** -120.42 (d, *J* = 11.2 Hz, 2F, F_C), -134.56 (d, *J* = 23.6 Hz, 4F, F_B), -163.58 (td, *J* = 23.5, 11.2 Hz, 2F, F_A) ppm; **HRMS (ESI)**: calcd for $C_{32}H_{21}F_8N_4O_4$ $[M+H]^+$ 677.1430, found 677.1428. **IR (neat) ν:** 3258.1, 1651.7, 1638.2, 1557.2, 1482.0, 1275.7 cm^{-1}.

3. Synthesis of the substrates and identification of the products

<u>Substrates:</u>

2-bromo-1,2-diphenylethyl(phenethyl)sulfane (4)

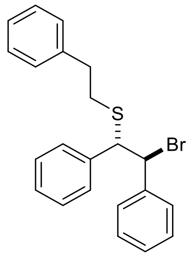

4

Compound **4** was synthesized following a synthetic route described in bibliography and showed identical spectroscopic data as those reported therein[2]; **^{1}H-NMR (300 MHz, $CDCl_3$, 298 K) δ**: 7.29 – 7.01 (m, 15H), 5.23 (d, *J* = 9.0 Hz, 1H), 4.45 (d, *J* = 9.0 Hz, 1H), 2.87 – 2.68 (m, 2H), 2.59 – 2.49 (m, 2H) ppm.

2-chloro-1,2-diphenylethyl(phenethyl)sulfane (4')

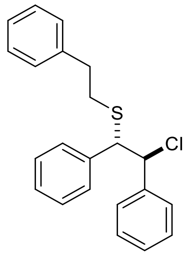

4'

Compound **4'** was synthesized following a synthetic route described in bibliography and showed identical spectroscopic data as those reported therein[2]; **^{1}H-NMR (300 MHz, $CDCl_3$, 298 K) δ**: 7.28 – 6.97 (m, 15H), 5.14 (d, *J* = 8.4 Hz, 1H), 4.34 (d, *J* = 8.4 Hz, 1H), 2.75 (m, 2H), 2.54 (t, *J* = 7.4 Hz, 2H) ppm.

1-(2-chloro-1,2-diphenylethyl)pyrrolidine (6)

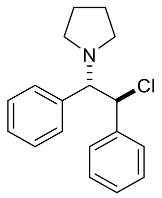

6

Compound **6** was synthesized following a synthetic route described in bibliography and showed identical spectroscopic data as those reported therein[3]; **^{1}H-NMR (300 MHz, $CDCl_3$, 298 K) δ**: 7.19 – 7.08 (m, 8H), 7.00 – 6.95 (m, 2H), 5.43 (d, *J* = 7.5 Hz, 1H), 4.07 (d, *J* = 7.5 Hz, 1H), 2.75 – 2.51 (m, 4H), 1.80 – 1.69 (m, 4H) ppm.

Products:

2-fluoro-1,2-diphenylethyl(phenethyl)sulfane (5)

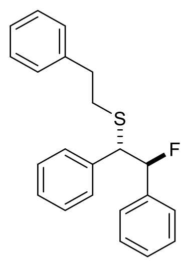

5

Compound **5** was synthesized according to the general procedure for catalytic phase-transfer nucleophilic fluorinations (see section 4) and purified by column chromatography (silica gel) using hexane/Et_2O (95:5 to 80:20). The product showed identical spectroscopic data as those reported in bibliography[2]; **^{1}H-NMR (400 MHz, $CDCl_3$, 298 K) δ**: 7.29 – 7.18 (m, 9H), 7.14 – 7.11 (m, 2H), 7.10 – 7.05 (m, 4H), 5.65 (dd, *J* = 46.3, 7.5 Hz, 1H), 4.20 (dd, *J* = 13.6, 7.5 Hz, 1H), 2.88 – 2.59 (m, 4H) ppm; **^{19}F-NMR (376 MHz, $CDCl_3$, 298 K) δ:** -172.60 (dd, *J* = 46.3, 13.5 Hz, 1F) ppm.

1-(2-fluoro-1,2-diphenylethyl)pyrrolidine (7)

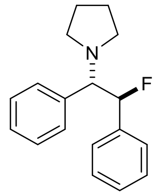

7

Compound **7** was synthesized according to the general procedure for catalytic phase-transfer nucleophilic fluorinations (see section 4) and purified by column chromatography (silica gel) using hexane/Et_2O (9:1 to 8:2). The product showed identical spectroscopic data as those reported in bibliography[3]; **^{1}H-NMR (400 MHz, $CDCl_3$, 298 K) δ**: 7.17 – 7.08 (m, 6H), 7.04 – 6.97 (m, 4H), 5.74 (dd, *J* = 46.9, 7.6 Hz, 1H), 3.69 (dd, *J* = 13.2, 7.6 Hz, 1H), 2.81 – 2.68 (m, 2H), 2.61 – 2.53 (m, 2H), 1.82 – 1.72 (m, 4H) ppm; **^{19}F-NMR (376 MHz, $CDCl_3$, 298 K) δ:** -170.86 (dd, *J* = 46.9, 13.2 Hz, 1F) ppm.

4. Phase-transfer nucleophilic fluorinations

In a 2 mL screw-cap vial (12 x 32 mm) equipped with a cylindric PTFE magnetic stir bar (5 x 2 mm) were sequentially added the appropriate substrate (0.025 mmol, 1 equiv.), the urea catalyst (2.5 – 10 mol%), the fluoride source (1.2 equiv.) and deuterated dichloromethane (0.1 mL, 0.25 M) as solvent. The vial was sealed, and the reaction mixture was stirred at 1200 rpm and at room temperature.

In a first approximation, three aliquots (25 µL) of the crude mixture were taken at different times and analysed by ^{1}H NMR to follow the course of the reaction. In this way the optimal reaction time for each substrate was estimated. The reactions were repeated under the same conditions during the optimal reaction time (10 h for substrate **6** and 24 h for substrate **4**) and after that, the reaction crudes were diluted with further deuterated dichloromethane (0.4 mL), transferred to NMR tubes and analysed by ^{1}H and ^{19}F NMR (4-fluoroanisole as internal standard) for a proper calculation of the NMR yield. The catalytic tests were conducted three times, yielding results with an error margin of less than 5%.

Table S1. Evaluation of different catalysts and conditions for the phase-transfer nucleophilic fluorination of substrates **4**.

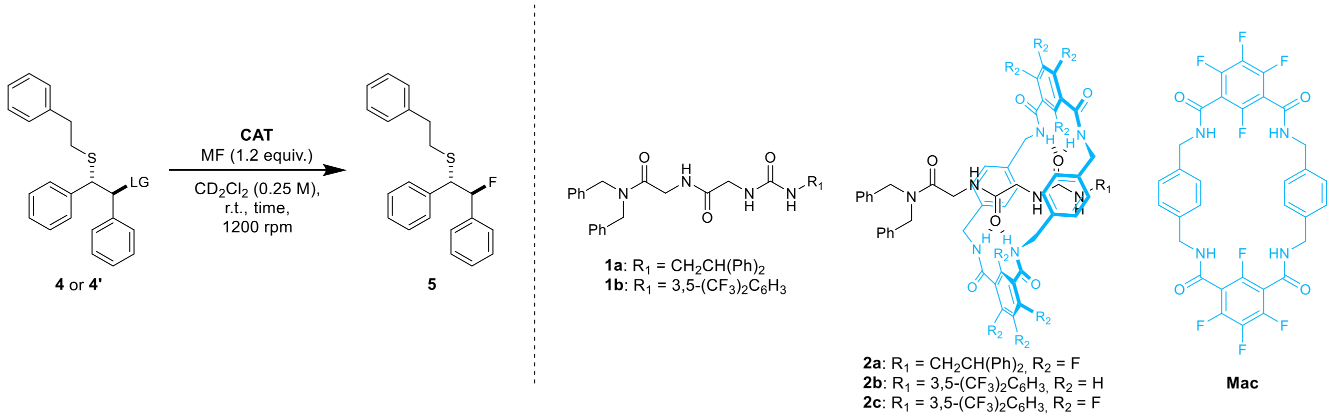

	Entry	LG	F$^-$ source*	Catalyst	Cat. Loading (mol %)	Time (h)	Yield (%)‡
Catalyst screening (threads *vs* rotaxanes)	1	Br	CsF	--	--	24	No reaction
	2	Br	CsF	**1a**	10	24	7
	3	Br	CsF	**1b**	10	24	8
	4	Br	CsF	**2a**	10	24	26
	5	Br	CsF	**2b**	10	24	30
	6	Br	CsF	**2c**	10	24	97
Stirring	7†	Br	CsF	**2c**	10	20	34
Catalyst loading	8	Br	CsF	**2c**	5	24	72
	9	Br	CsF	**2c**	2.5	24	12
Leaving group	10	Cl	CsF	**2c**	10	24	10

F⁻ source	11	Br	NaF	**2c**	10	24	No reaction
	12	Br	KF	**2c**	10	24	16
Interlocked *vs* Non-interlocked components	13	Br	CsF	**Mac**	10	24	No reaction
	14	Br	CsF	**Mac + 1b**	10	24	9

General conditions: Substrate (0.025 mmol), 0.1 mL of CD_2Cl_2, stirring at 1200 rpm; * Flouride source (CsF, NaF and KF) used as provided by the supplier without any prior drying; ‡ Determined by ^{19}F NMR using 4-fluoroanisole as internal standard; † Stirring at 600 rpm.

Table S2. Evaluation of different catalysts for the phase-transfer nucleophilic fluorination of substrate **6**.

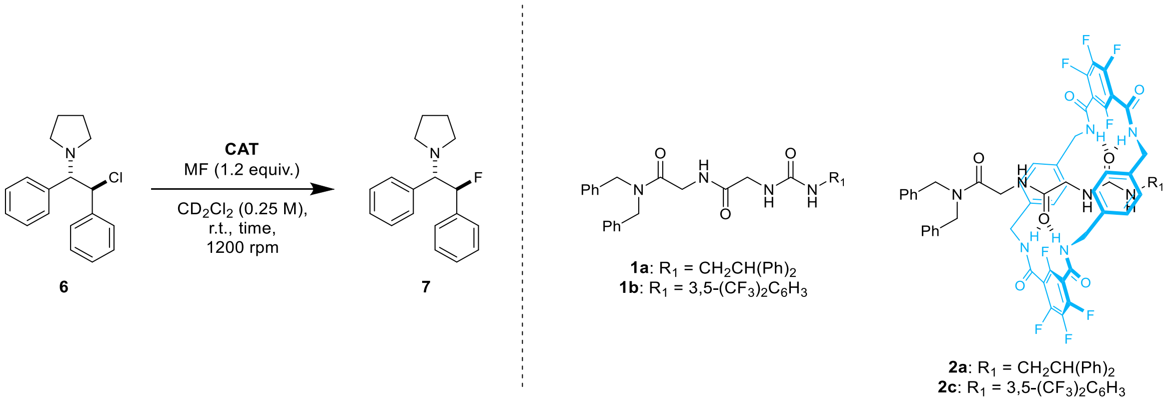

	Entry	LG	F⁻ source*	Catalyst	Cat. Loading (mol %)	Time (h)	Yield (%)‡
Catalyst screening (threads *vs* rotaxanes)	1	Cl	CsF	--	--	10	No reaction
	2	Cl	CsF	**1a**	5	10	No reaction
	3	Cl	CsF	**1b**	5	10	23
	4	Cl	CsF	**2a**	5	10	13
	5	Cl	CsF	**2c**	5	10	99
	6	Cl	KF	--	--	10	No reaction
	7	Cl	KF	**1a**	5	10	13
	8	Cl	KF	**1b**	5	10	32
	9	Cl	KF	**2a**	5	10	30
	10	Cl	KF	**2c**	5	10	80

General conditions: Substrate (0.025 mmol), 0.1 mL of CD_2Cl_2, stirring at 1200 rpm; * Flouride source (CsF and KF) used as provided by the supplier without any prior drying; ‡ Determined by ^{19}F NMR using 4-fluoroanisole as internal standard.

5. Estimation of the pKa for the deprotonation of model amides

The pK_a of the NH amide groups has been estimated for two model molecules: a) *N*-benzylbenzamide and b) *N*-benzyl-2,3,4,5,6-pentafluorobenzamide, which mimic the amides present at the macrocycle in rotaxanes **2b** and **2a**, **2c**, respectively (Scheme S4). The calculations were carried out using the software package Marvin Suite version 5.11.5 and chemicalize.org, both developed by ChemAxom.[4] The obtained data indicates the main acidic character of the NH group in the *N*-benzyl-2,3,4,5,6-pentafluorobenzamide (rotaxanes **2a**, **2c**) compared to that of *N*-benzylbenzamide (rotaxane **2b**).

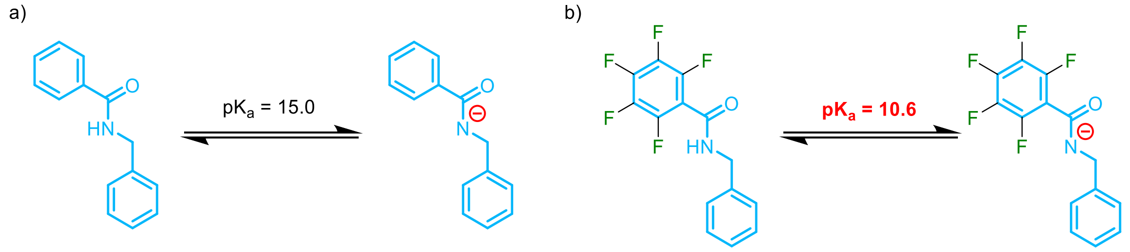

Scheme S4. Calculation of the pK_a for the deprotonation of: a) *N*-benzylbenzamide and b) *N*-benzyl-2,3,4,5,6-pentafluorobenzamide.

6. Crystal data and structure refinements

Thread 1b

Single crystals of $C_{27}H_{24}F_6N_4O_3$ [JPA_III_63_TBAF_0msp_a] were obtained by slow diffusion of hexane in a solution of $CHCl_3$. Intensities were registered at low temperature (100.0 K) on a Bruker D8 QUEST system equipped with a multilayer monochromator and a Mo K/a Incoatec microfocus sealed tube (λ = 0.71073 Å). Absorption corrections were based on multi-scans (program SADABS). Using Olex2,[5] the structure was solved with the SHELXT structure solution program using Intrinsic Phasing and refined with the SHELXL refinement package using Least Squares minimisation.[6,7] Hydrogen atoms were included using a riding model. The structure was deposited with CSD (deposition number CCDC-2349096).

Table S3. Crystal data and structure refinement for **1b**.

Empirical formula	$C_{27}H_{24}F_6N_4O_3$	
Formula weight	566.50	
Temperature	100(2) K	
Wavelength	0.71073 Å	
Crystal system	Triclinic	
Space group	P-1	
Unit cell dimensions	a = 8.9570(3) Å	α = 69.3350(10) °
	b = 11.6096(4) Å	β = 84.5020(10) °
	c = 13.8227(4) Å	γ = 71.0660(10) °
Volume	1271.85(7) $Å^3$	
Z	2	
Density (calculated)	1.479 g/cm^3	
Absorption coefficient	0.127 mm^{-1}	
F(000)	584.0	
Crystal size	0.24 x 0.14 x 0.09 mm^3	
Theta range for data collection	3.946 to 61.338 °	
Index ranges	$-12<h<12, -16<k<16, -19<l<19$	
Reflections collected	107962	
Independent reflections	7860 [R_{int}= 0.0343]	
Absorption correction	Semi-empirical from equivalents	
Refinement method	Full-matrix least-squares on F^2	
Data / restraints / parameters	7860/0/361	
Goodness-of-fit on F^2	1.087	
Final R indices [I>2sigma(I)]	R_1= 0.0569, wR_2= 0.1268	
R indices (all data)	R_1= 0.0781, wR_2= 0.1513	
Largest diff. peak and hole	0.62/-0.61 e.$Å^{-3}$	

Table S4. Intermolecular hydrogens bonds for **1b** [Å and (°)]

D-H...A	d(D-H)	d(H...A)	d(D...A)	<(DHA)
N2H2…O3[1]	0.88	2.15	2.8690(17)	138.0
N3H3…O2[2]	0.88	2.20	2.9192(18)	138.8
N4H4…O1[2]	0.88	1.97	2.8350(18)	168.8

Symmetry transformations used to generate equivalent atoms:
[1] 2-X,1-Y,1-Z; [2] 1-X,1-Y,1-Z

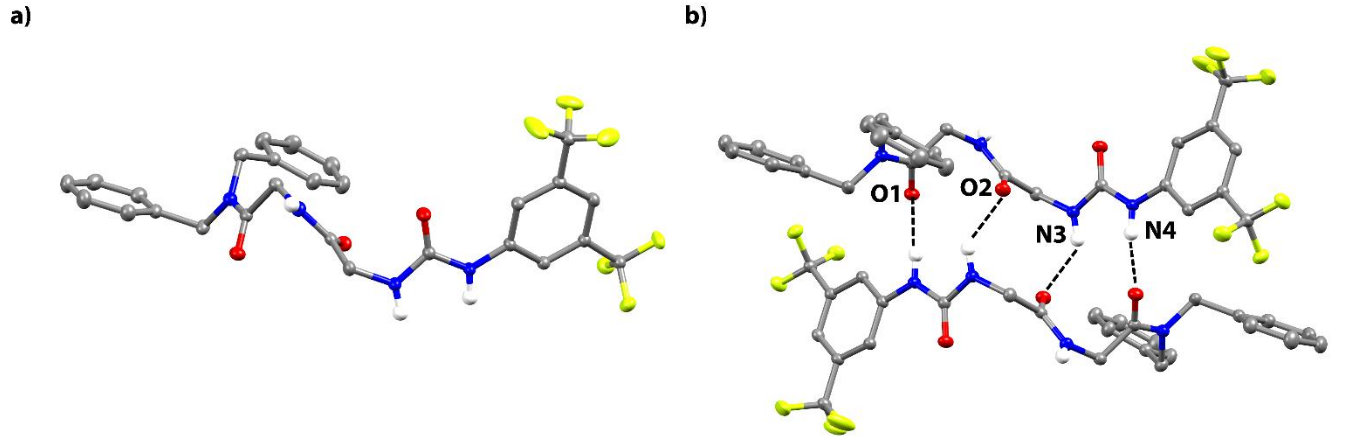

Figure S1. Molecular structure of thread **1b** with thermal ellipsoids drawn at 50% probability: a) lateral view; b) homodimerization of thread **1b**. For clarity, selected hydrogens atoms have been deleted.

Rotaxane 2c

Single crystals of $C_{60}H_{45}Cl_3F_{14}N_8O_7$ [JPA_III_69_D_0msp_a] were obtained by slow diffusion of hexane in a solution of $CHCl_3$. Intensities were registered at low temperature (100.0 K) on a Bruker D8 QUEST system equipped with a multilayer monochromator and a Mo K/a Incoatec microfocus sealed tube (λ = 0.71073 Å). Absorption corrections were based on multi-scans (program SADABS). Using Olex2,[5] the structure was solved with the SHELXT structure solution program using Intrinsic Phasing and refined with the SHELXL refinement package using Least Squares minimisation.[6,7] Hydrogen atoms were included using a riding model. Two molecules of chloroform are included in the unit cell. The structure was deposited with CSD (deposition number CCDC-2349097).

Table S5. Crystal data and structure refinement for **2c**.

Empirical formula	$C_{60}H_{45}Cl_3F_{14}N_8O_7$	
Formula weight	1362.39	
Temperature	100(2) K	
Wavelength	0.71073 Å	
Crystal system	Triclinic	
Space group	P-1	
Unit cell dimensions	a = 13.9699(6) Å	α = 91.362(2)°
	b = 14.1740(6) Å	β = 94.825(2)°
	c = 14.9438(6) Å	γ = 96.724(2)°
Volume	2926.6(2) Å^3	
Z	2	
Density (calculated)	1.546 g/cm^3	
Absorption coefficient	0.264 mm^{-1}	
F(000)	1388.0	
Crystal size	0.19 x 0.04 x 0.04 mm^3	
Theta range for data collection	3.842 to 59.284°	
Index ranges	-19<h<19, -19<k<19, -20<l<20	
Reflections collected	150654	
Independent reflections	16488 [R_{int}= 0.0396]	
Absorption correction	Semi-empirical from equivalents	
Refinement method	Full-matrix least-squares on F^2	
Data / restraints / parameters	16488/0/858	
Goodness-of-fit on F^2	1.058	
Final R indices [I>2sigma(I)]	R1= 0.0379, wR2= 0.0883	
R indices (all data)	R1= 0.0548, wR2= 0.1017	
Largest diff. peak and hole	0.52 and -0.69 e.Å^{-3}	

Table S6. Polar hydrogens bonds for **2c** [Å and (°)]

**D-H...A**	**d(D-H)**	**d(H...A)**	**d(D...A)**	**<(DHA)**
N2H2…O5[1]	0.85(2)	2.10(2)	2.9359(16)	168.6(18)
N4H4…O6	0.81(2)	2.02(2)	2.8314(16)	175(2)
N5H5…O3	0.84(2)	2.56(2)	3.3655(16)	163.4(18)
N6H6…O3	0.88(2)	2.20(2)	3.0569(17)	166.3(17)
N7H7…O4[2]	0.86(2)	2.27(2)	2.9585(17)	136.0(19)
N8H8…O1	0.83(2)	2.11(2)	2.7630(16)	136.2(18)

Symmetry transformations used to generate equivalent atoms:
[1] 1-X,1-Y,1-Z; [2] +X,-1+Y,+Z;

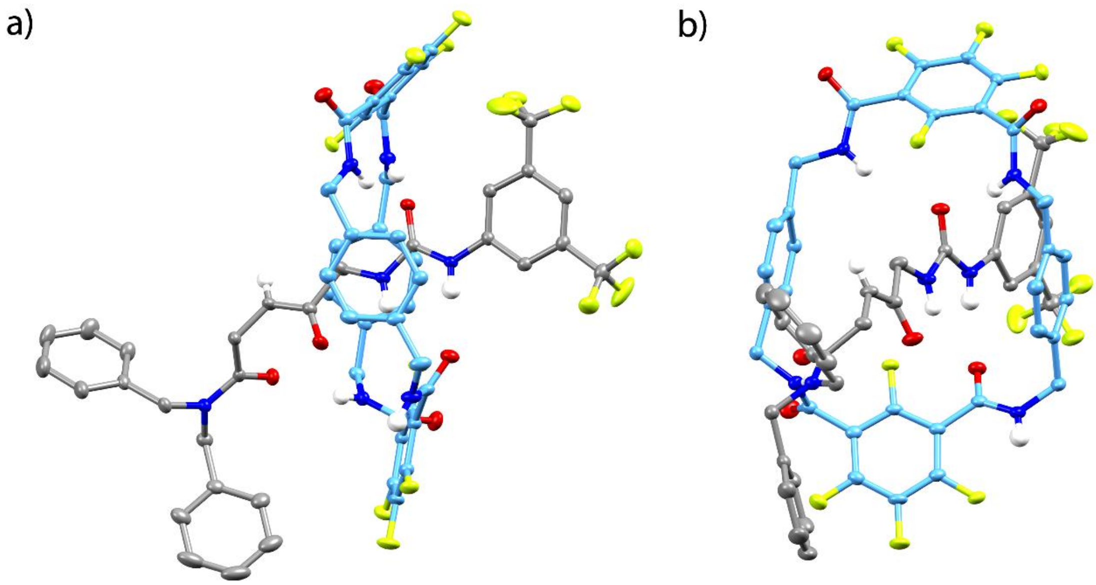

Figure S2. Molecular structure of **2c** with thermal ellipsoids drawn at 50% probability: a) lateral view; b) tilted view. For clarity, selected hydrogens atoms and solvent molecules have been deleted.

7. Variable concentration studies

To gain valuable information about the intermolecular self-interactions in the thread **1b** and in the rotaxanes **2b** and **2c** in non-competitive solvents, ^{1}H NMR spectra in CD_2Cl_2 were recorded at increasing concentrations (0.25 – 3.0 mM) and the shift of the signals related with the NHs of the molecules was analysed. For the thread **1b** and the rotaxane **2c**, dimeric self-aggregation constants were calculated using free online tool Bindfit software (Supramolecular.org).[8] The software plots the shift of the signals against the concentration of the specie and refines using nonlinear least-squares analysis to obtain the best fit between empirical and calculated data. For the rotaxane **2b** no variation of the signals shift was observed with increasing concentration.

Thread 1b

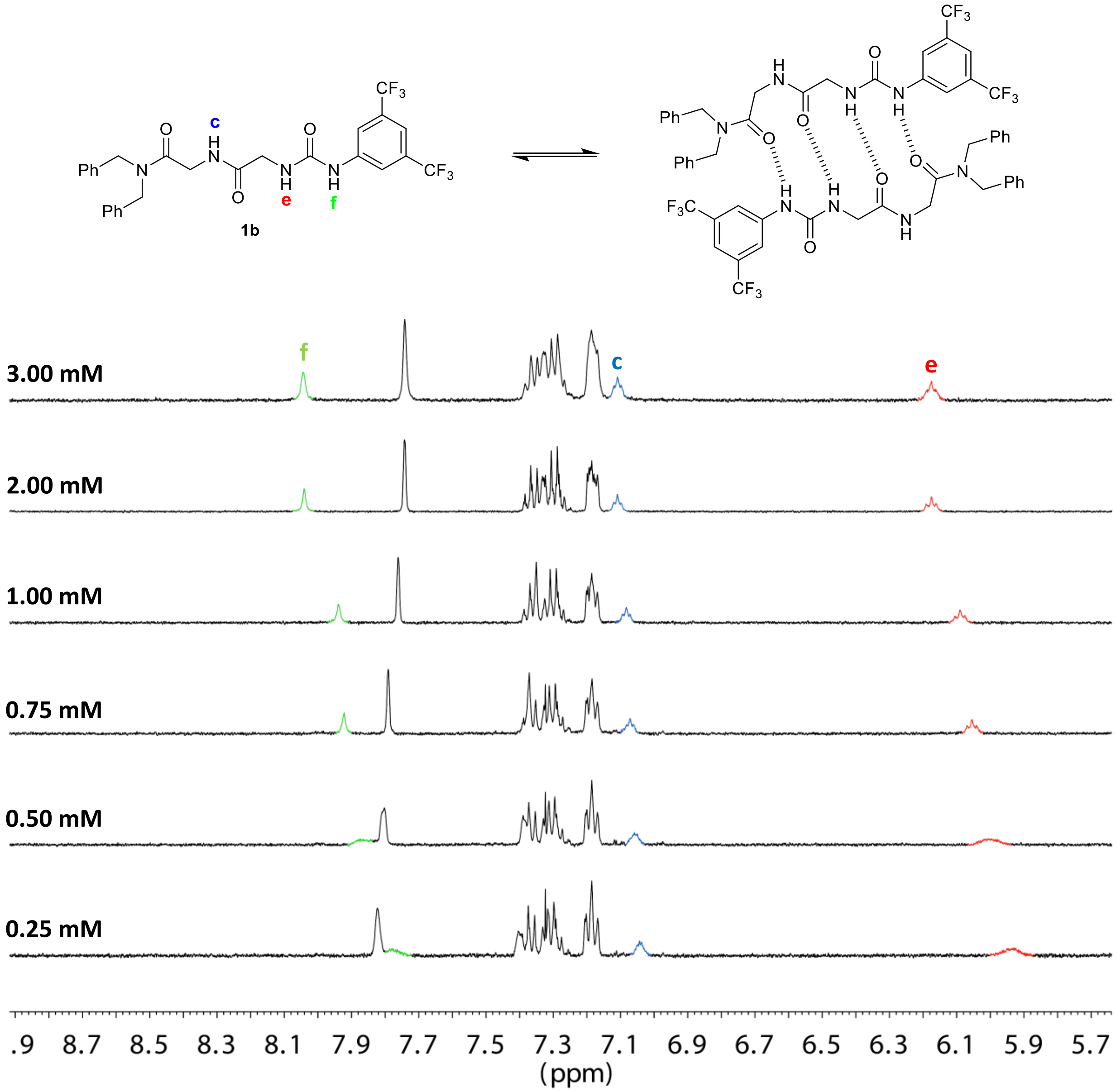

Figure S3. Stacked partial of ^{1}H NMR spectra (400 MHz, CD_2Cl_2) of thread **1b** at 0.25 to 3 mM concentration.

Fitter	NMR dimer aggregation
K guess	100
Method	Nelder-Mead
Fitted datapoints	18
Fitted parameters	7
K_{dim}	**3643 M^{-1}**
K_{dim} error	7%
SSR[a]	0.001

[a]Sum of squares residuals.

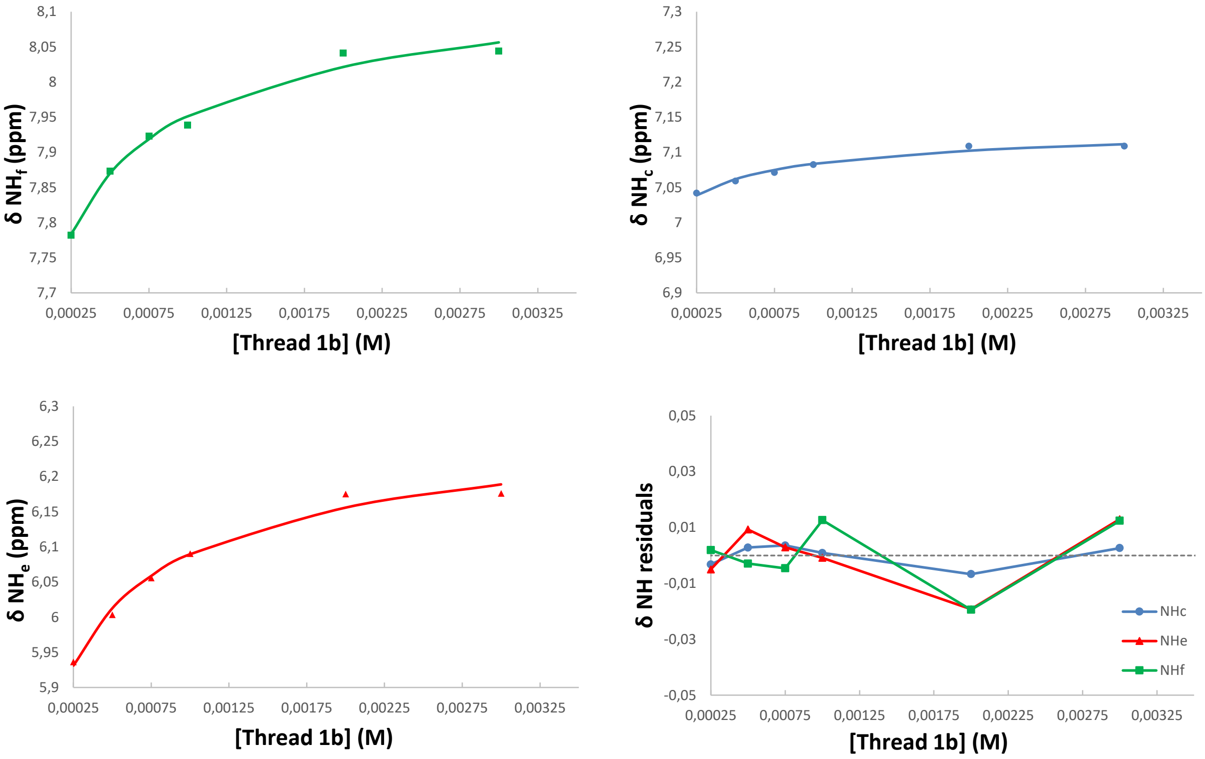

Figure S4. Input and output parameters of fitting for thread **1b** (top); fitting plots for NH_f (green), NH_c (blue) and NH_e (red) signals, experimental (shapes) and fitted function (lines) and residual plot of the fitting. Link to the open fitting data: http://app.supramolecular.org/bindfit/view/0bd18ad4-e42c-4f27-a669-3a529dc87fa6

Rotaxane 2b

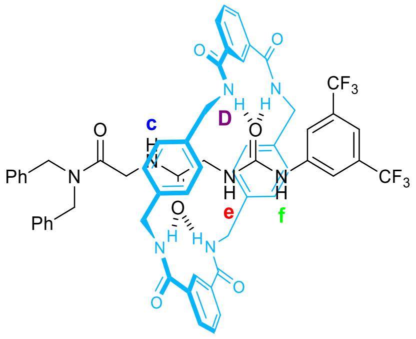

2b

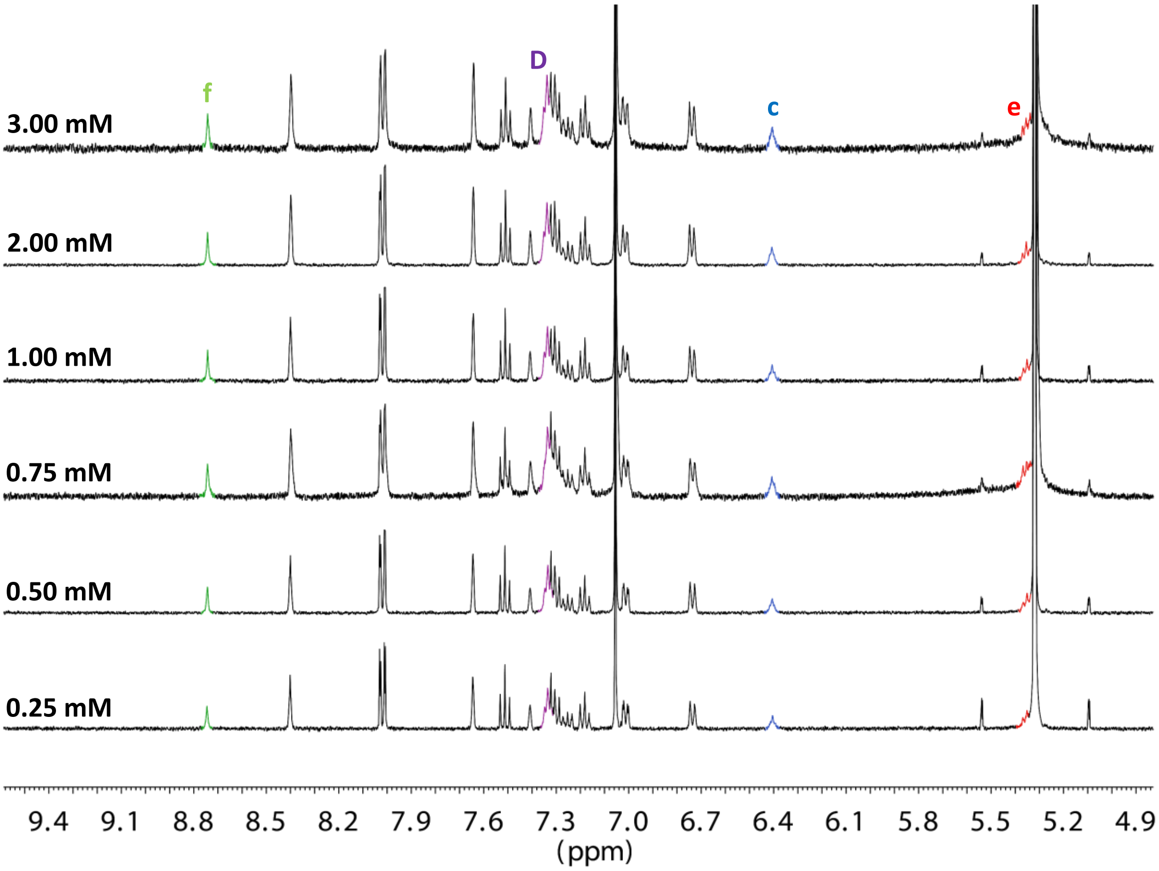

Figure S5. Stacked partial of ^{1}H NMR spectra (400 MHz, CD_2Cl_2) of rotaxane **2b** at 0.25 to 3 mM concentration.

Rotaxane 2c

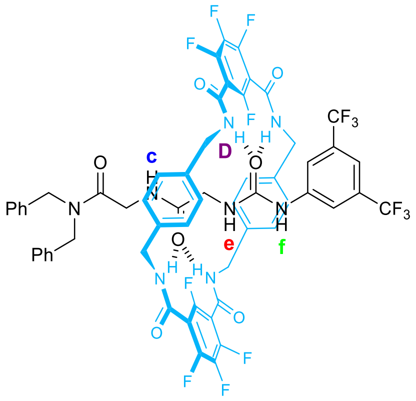

2c

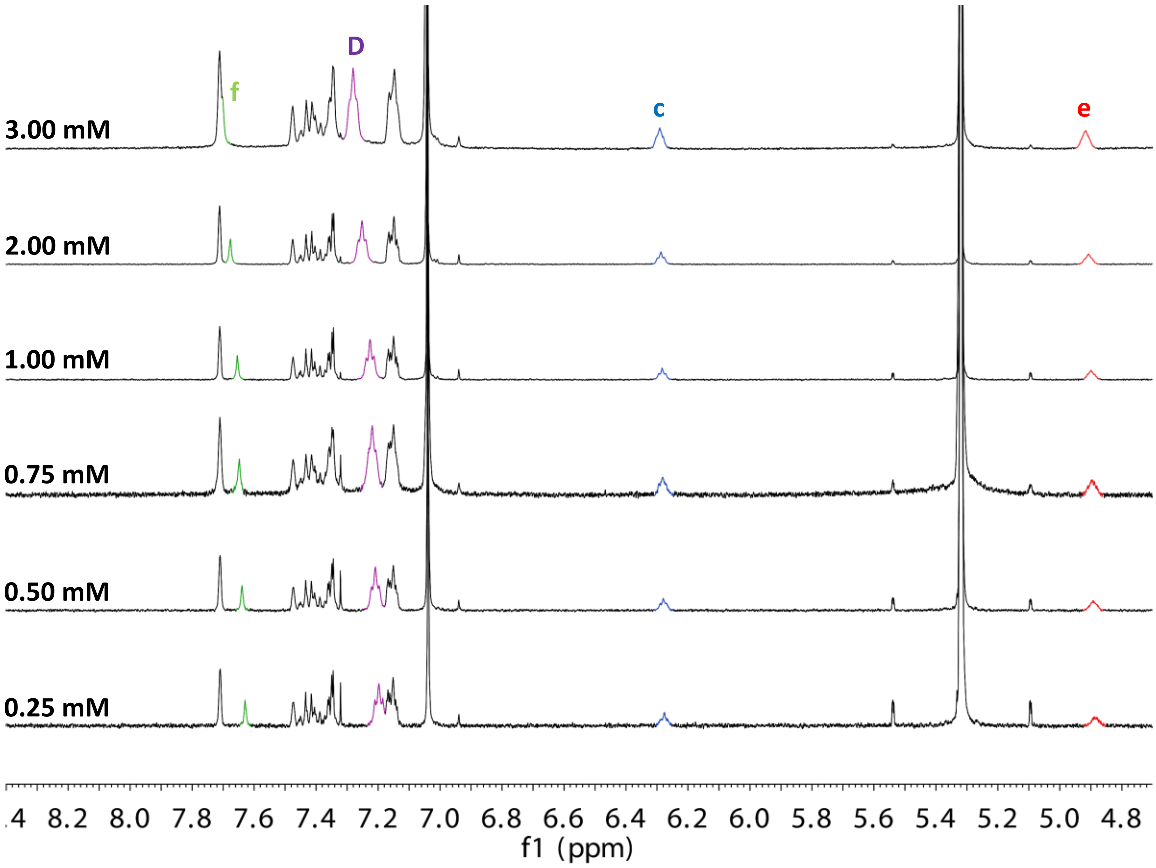

Figure S6. Stacked partial of ^{1}H NMR spectra (400 MHz, CD_2Cl_2) of rotaxanes **2c** at 0.25 to 3 mM concentration.

Fitter	NMR dimer aggregation
K guess	100
Method	Nelder-Mead
Fitted datapoints	18
Fitted parameters	7
K_{dim}	**63 M^{-1}**
K_{dim} error	3%
SSR[a]	0.00004

[a]Sum of squares residuals.

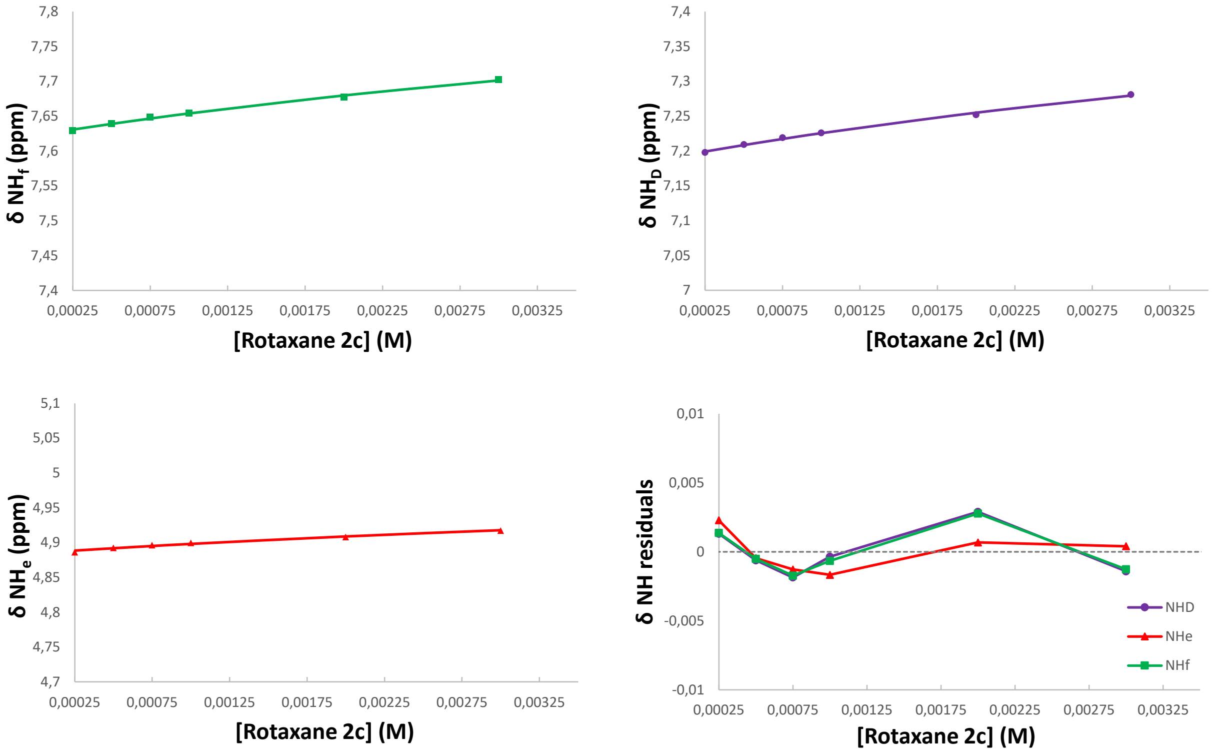

Figure S7. Input and output parameters of fitting of rotaxane **2c** (top); fitting plots for NH_f (green), NH_D (purple) and NH_e (red) signals, experimental (shapes) and fitted function (lines) and residual plot of the fitting. Link to the open fitting data: http://app.supramolecular.org/bindfit/view/ea36876b-e0d5-4e4b-9e08-d48b31d472f6

8. Fluoride binding studies

8.1. UV titrations

A 0.02 mM solution of urea host (thread **1b** or rotaxanes **2b** and **2c**) in DCM was prepared. A solution of approximately 1 mM of fluoride in DCM was prepared using TBAF·3H_2O as guest source. To avoid the dilution effect, this solution was also 0.02 mM in the host compound. The exact concentration of fluoride was determined by quantitative ^{19}F NMR using an aliquot of knowing volume and 4-Fluoroanisole as internal standard.[9] 2.5 mL of urea host solution were transferred into a quartz cuvette, and aliquots of TBAF·3H_2O solution (10-50 µL) were successively added until reaching an excess of fluoride equivalents. After each addition, a UV absorption spectrum (200-700 nm) was recorded.

Association constants (K_a) were determined from the titration data points by fitting the most suitable model using free online tool Bindfit software (Supramolecular.org).[8] The absorption at three wavelengths (around the new λ_{max}), host, and fluoride concentration were entered into the Bindfit software. The software plots the absorptions against the concentration of fluoride and refines using nonlinear least-squares analysis to obtain the best fit between empirical and calculated signals based on different host-guest binding models. The input parameters were varied until achieving optimal fitting values for the association constants.

Thread 1b

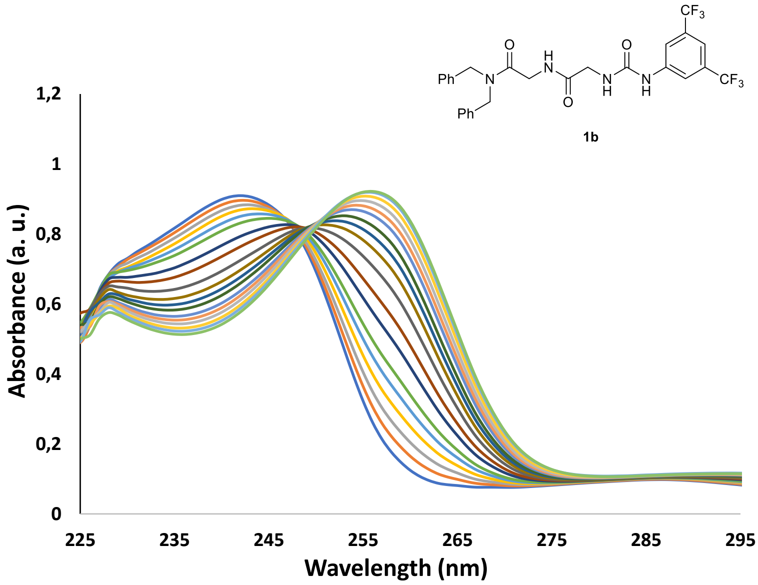

Fitter	UV 2:1
$K_{(1:1)}$ guess	1000000 M^{-1}
$K_{(2:1)}$ guess	100000 M^{-1}
Method	Nelder-Mead
Fitted datapoints	54
Fitted parameters	8
$K_{a\,(1:1)}$	**1039536 M^{-1}**
$K_{a\,(1:1)}$ error	4.3 %
$K_{a\,(2:1)}$	**111585 M^{-1}**
$K_{a\,(2:1)}$ error	7.7 %
SSR[a]	0.0009

[a]Sum of squares residuals.

Figure S8. Stacked UV spectra for the titration of thread **1b** with TBAF·3H_2O in DCM at 298 K (left); input and output parameters of fitting (right). Link to the open fitting data: http://app.supramolecular.org/bindfit/view/a2e1b488-24c1-4a74-8890-2e9ab801275c

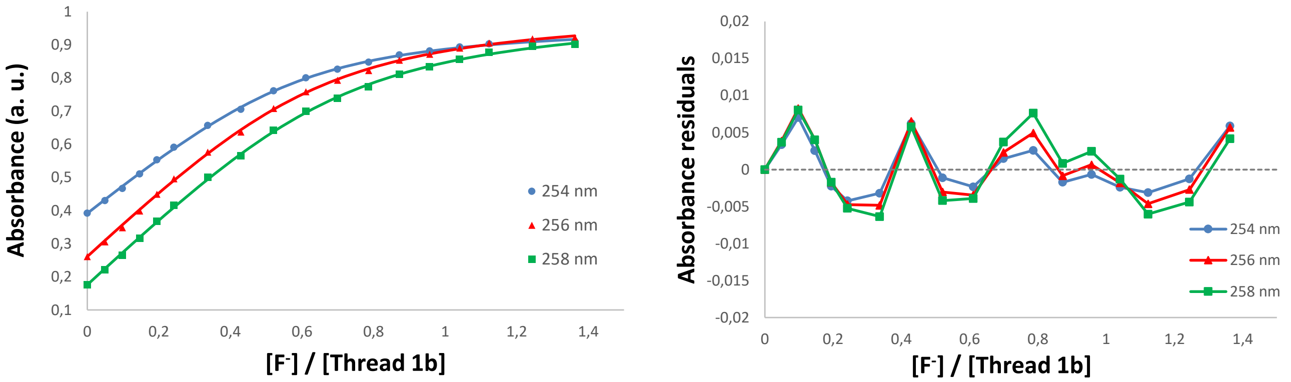

Figure S9. Titration profiles at 254, 256 and 258 nm, experimental (shapes) and fitted function (lines) (left); residual plot of the fitting (right).

Rotaxane 2b

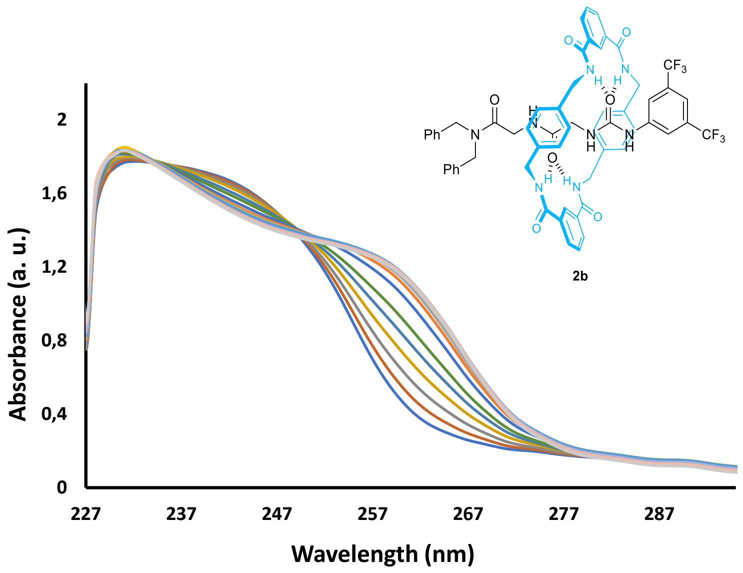

Fitter	UV 1:1
K guess	100 M^{-1}
Method	Nelder-Mead
Fitted datapoints	42
Fitted parameters	4
$K_{a\,(1:1)}$	**356978 M^{-1}**
$K_{a\,(1:1)}$ error	13%
SSR[a]	0.01

[a]Sum of squares residuals.

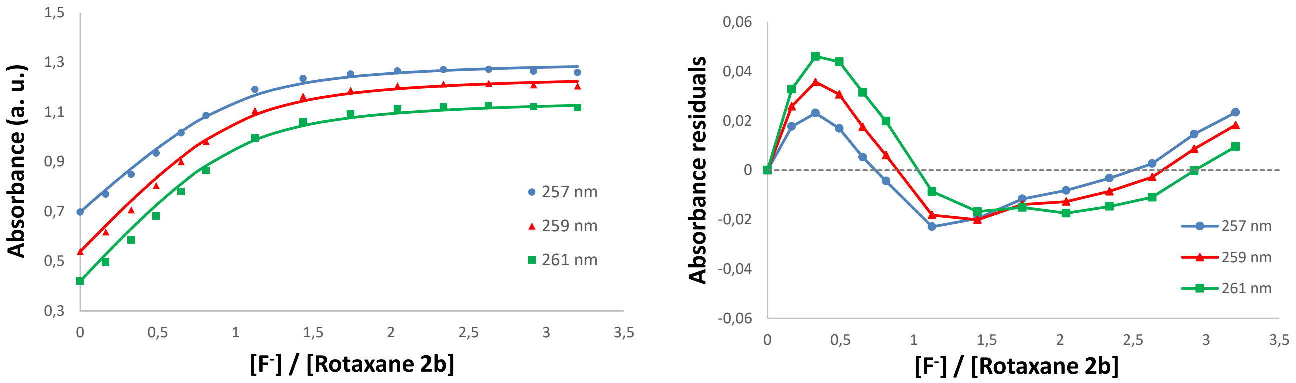

Figure S10. Stacked UV spectra for the titration of rotaxane **2b** with TBAF·3H_2O in DCM at 298 K (top left); input and output parameters of fitting (right top); titration profiles at 257, 259 and 261 nm, experimental (shapes) and fitted function (lines) (left bottom); residual plot of the fitting (right bottom). Link to the open fitting data: http://app.supramolecular.org/bindfit/view/1c5b2b15-f952-41f4-9b95-13207d294659

Rotaxane 2c

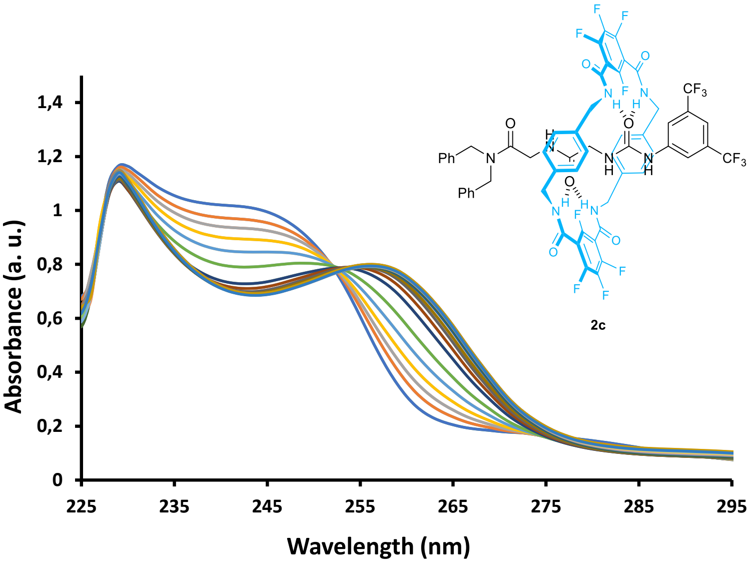

Fitter	UV 1:1
K guess	100 M^{-1}
Method	Nelder-Mead
Fitted datapoints	69
Fitted parameters	4
$K_{a\ (1:1)}$	**257297 M^{-1}**
$K_{a\ (1:1)}$ error	12 %
SSR[a]	0.01

[a]Sum of squares residuals.

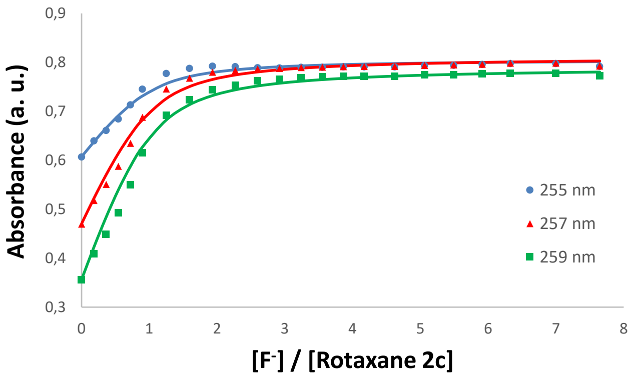

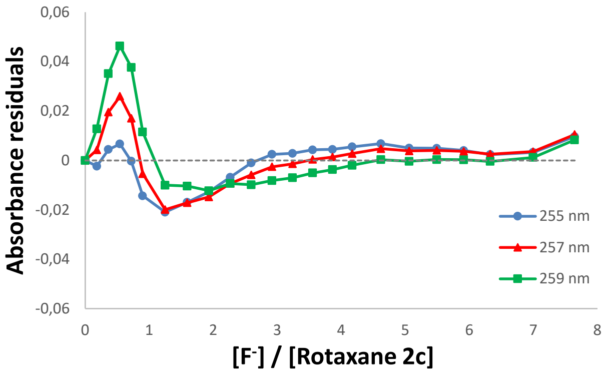

Figure S11. Stacked UV spectra for the titration of rotaxane **2c** with TBAF·3H_2O in DCM at 298 K (top left); input and output parameters of fitting (right top); titration profiles at 255, 257 and 259 nm, experimental (shapes) and fitted function (lines) (left bottom); residual plot of the fitting (right bottom). Link to the open fitting dat: http://app.supramolecular.org/bindfit/view/1a32ea91-0645-4460-8fc9-8a0929f30277

8.2. NMR titrations

A 1 mM solution of urea host (thread **1b** or rotaxanes **2b** and **2c**) in DCM-d_2 was prepared. A solution of approximately 20 mM of fluoride in DCM-d_2 was prepared using TBAF·3H_2O as guest source. To avoid the dilution effect, this solution was also 1 mM in the host compound. The exact concentration of fluoride was determined by quantitative ^{19}F NMR using an aliquot of knowing volume and 4-Fluoroanisole as internal standard.[9] 0.5 mL of urea host solution were transferred into a NMR tube, and aliquots of TBAF·3H_2O solution (3-50 µL) were successively added until reaching an excess of fluoride equivalents. After each addition, a ^{1}H and ^{19}F NMR spectra were recorded. As association constants were $K > 10^5$ M^{-1}, it was not possible to estimate their values accurately by NMR.[8b] Instead, they were calculated by UV titrations (see section 8.1). In addition, the binding stoichiometry of the urea hosts with fluoride was estimated using the method of continuous variation (Job's method).[10]

Thread 1b

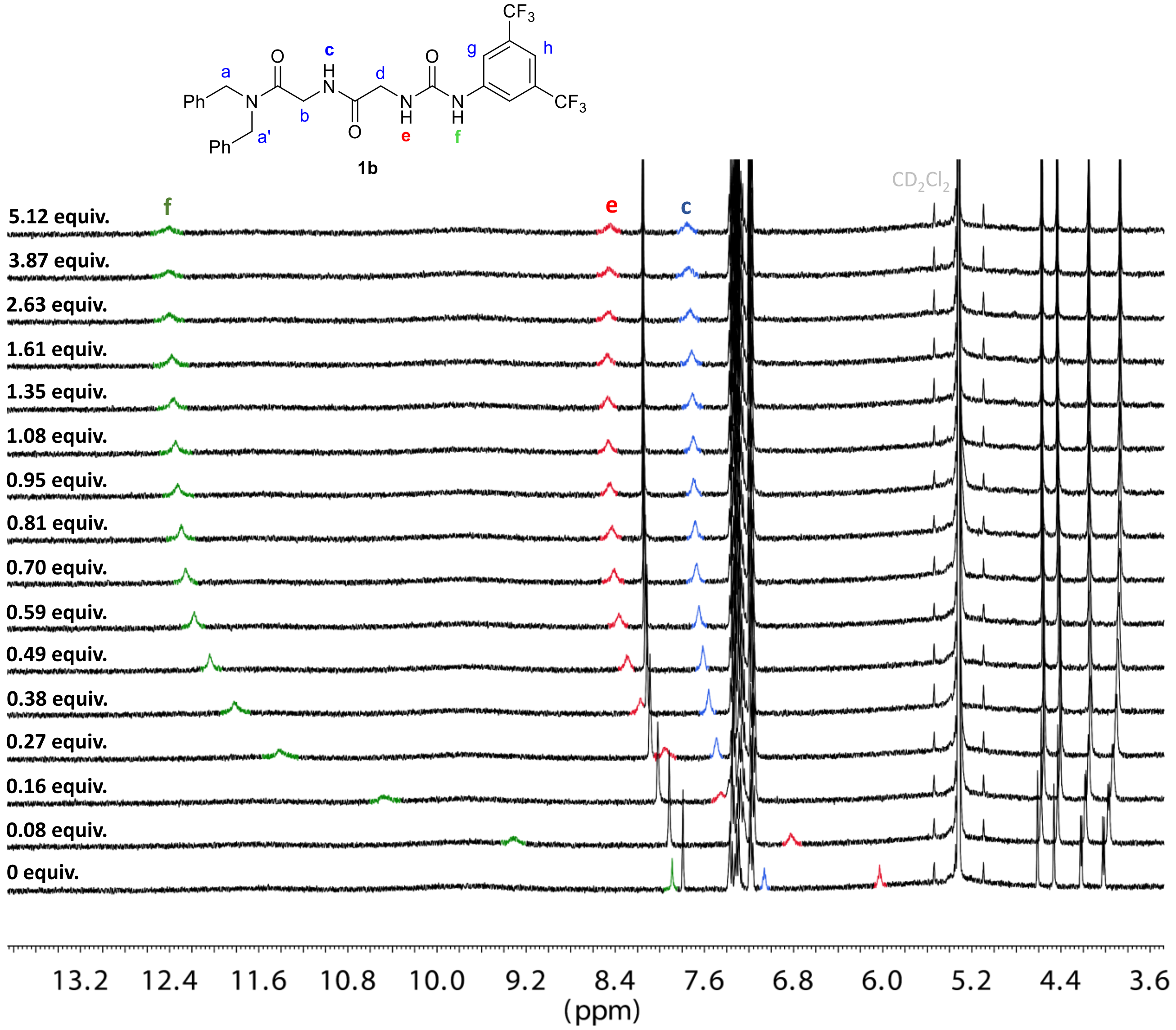

Figure S12. Stacked partial ^{1}H NMR spectra (400 MHz) for the titration of thread **1b** with TBAF·3H_2O in DCM-d_2 at 298 K. Easy to see the shifting of NHs signals.

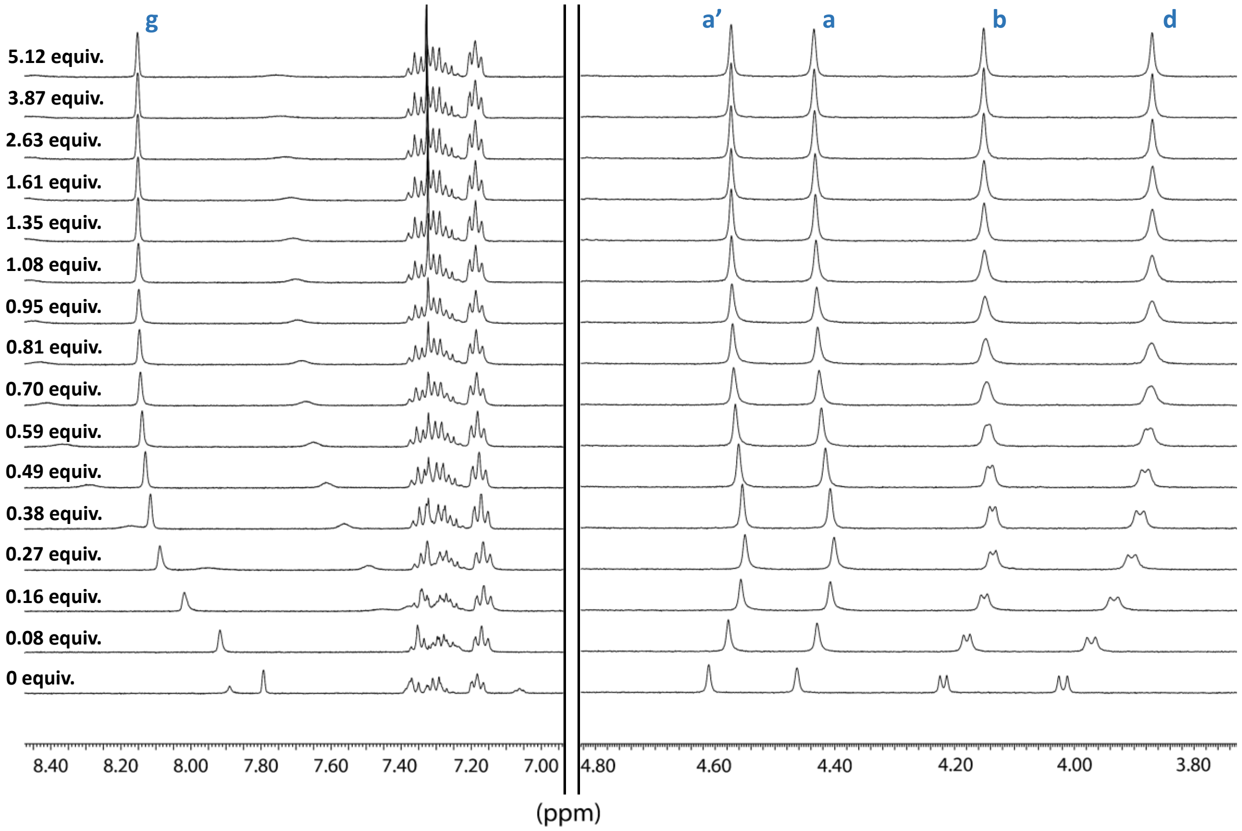

Figure S13. Stacked amplified ^{1}H NMR spectra (400 MHz) for the titration of thread **1b** with TBAF·3H_2O in DCM-d_2 at 298 K. Easy to see the shifting of aromatic (left) and methylenes (right) signals.

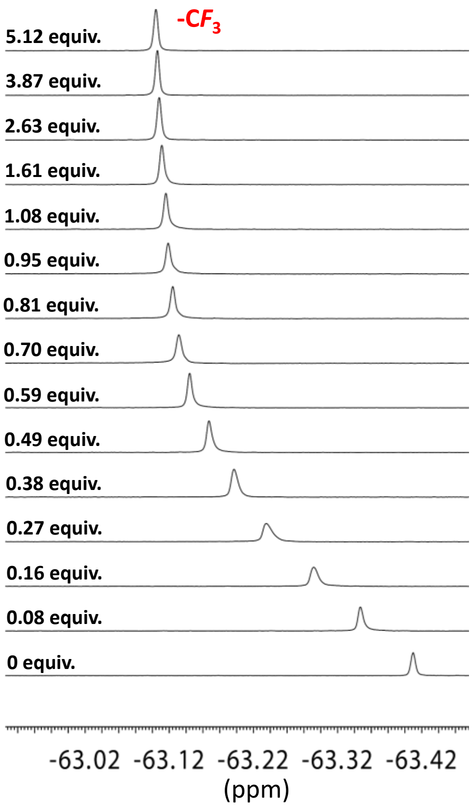

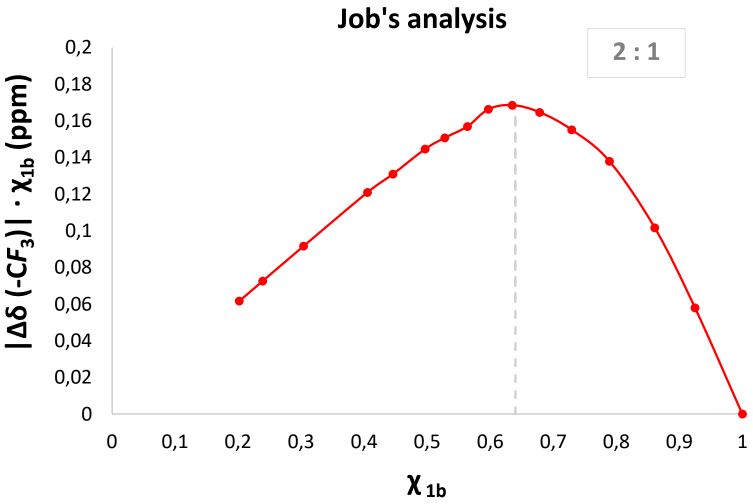

Figure S14. Stacked amplified ^{19}F NMR spectra (400 MHz) for the titration of thread **1b** with TBAF·3H_2O in DCM-d_2 at 298 K (left); Job's plot for Δδ (-C*F*$_3$) signal for titration of thread **1b** with TBAF·3H_2O in DCM-d_2 at 298 K. A maximum near 0.66 molar fraction of thread **1b** points towards the 2:1 stoichiometry in a **(1b)$_2$:F$^-$** complex.

Rotaxane 2b

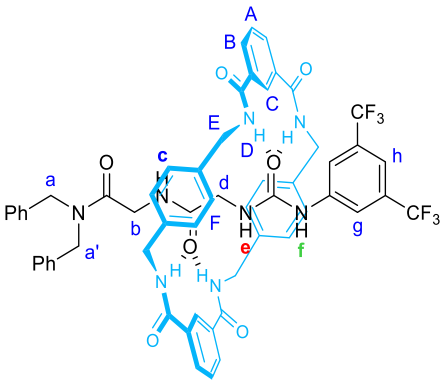

2b

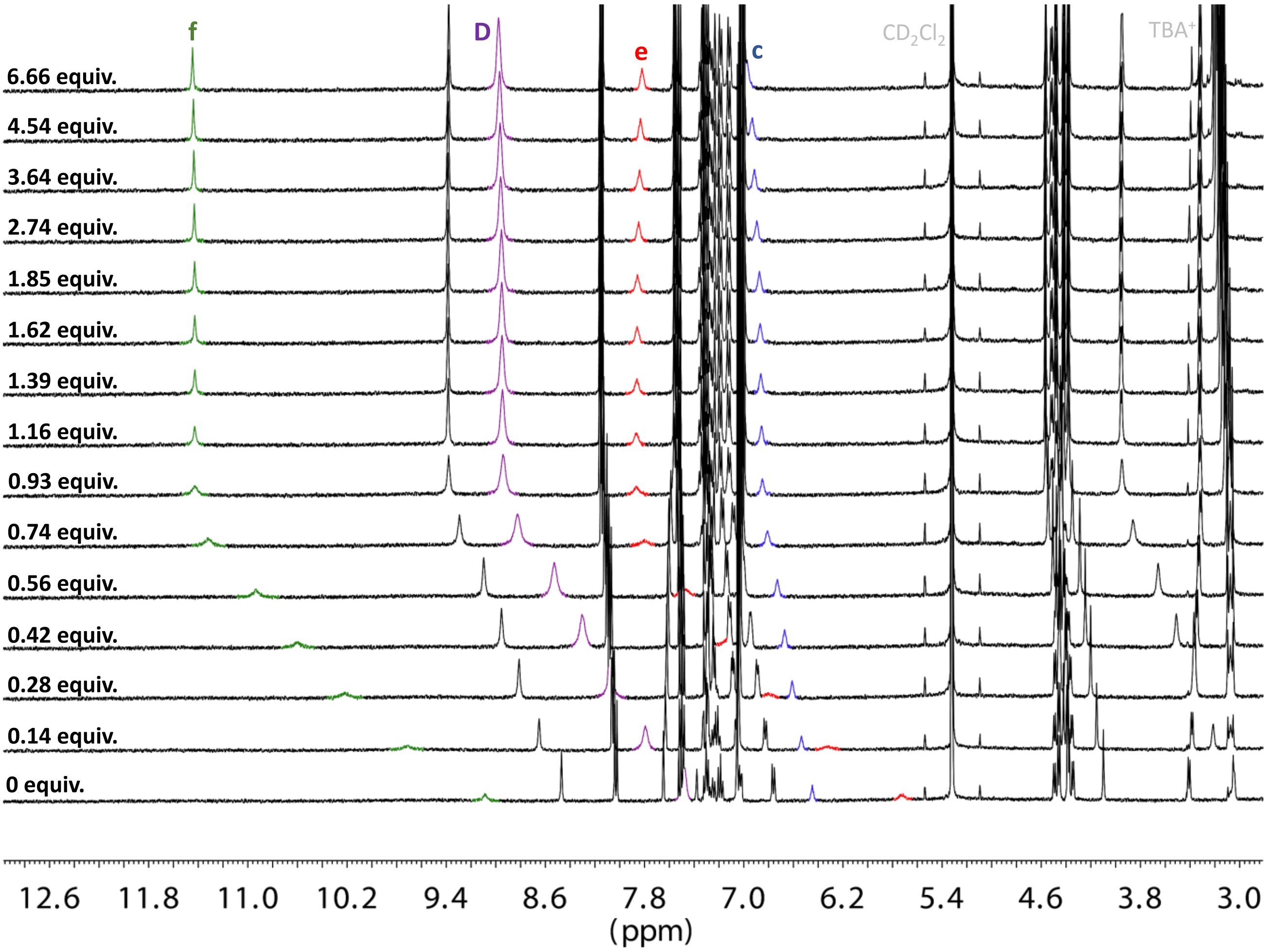

Figure S15. Stacked partial ^{1}H NMR spectra (400 MHz) for the titration of rotaxane **2b** with TBAF·3H_2O in DCM-d_2 at 298 K. Easy to see the shifting of NHs signals.

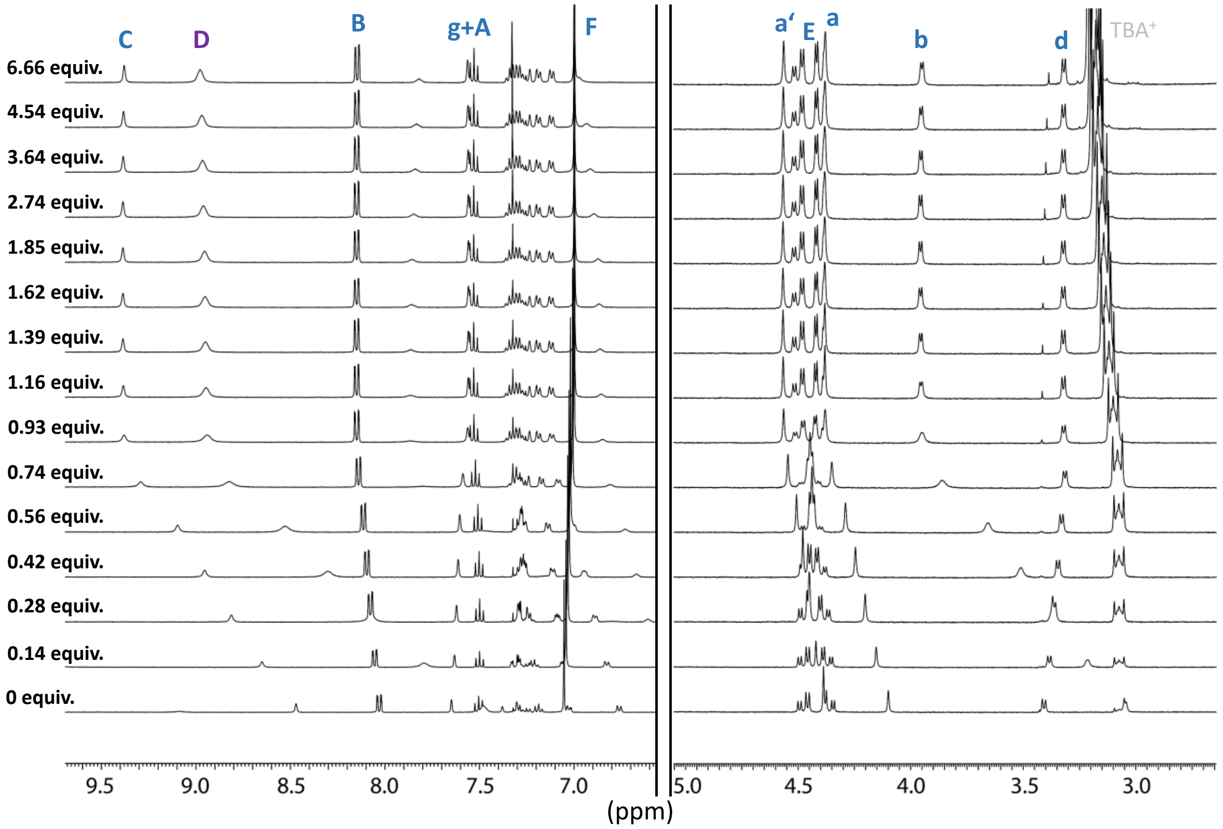

Figure S16. Stacked amplified ^{1}H NMR spectra (400 MHz) for the titration of rotaxane **2b** with TBAF·3H_2O in DCM-d_2 at 298 K. Easy to see the shifting of aromatic (left) and methylenes (right) signals.

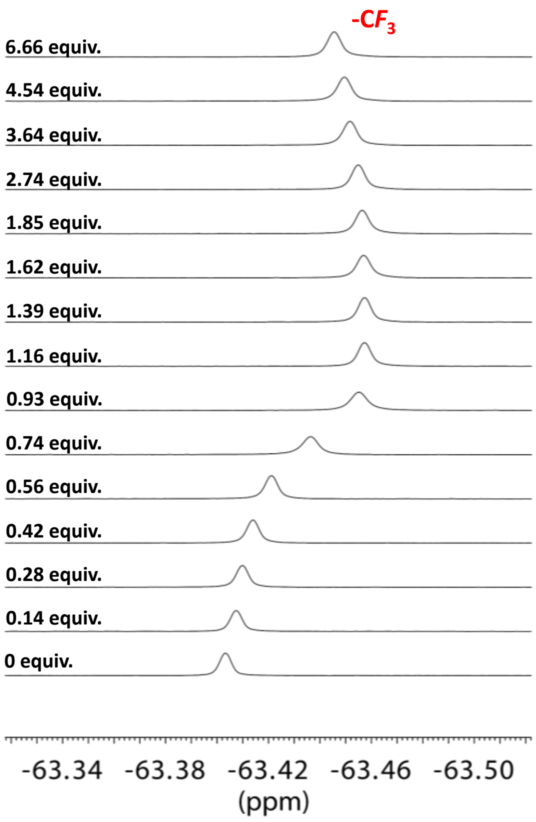

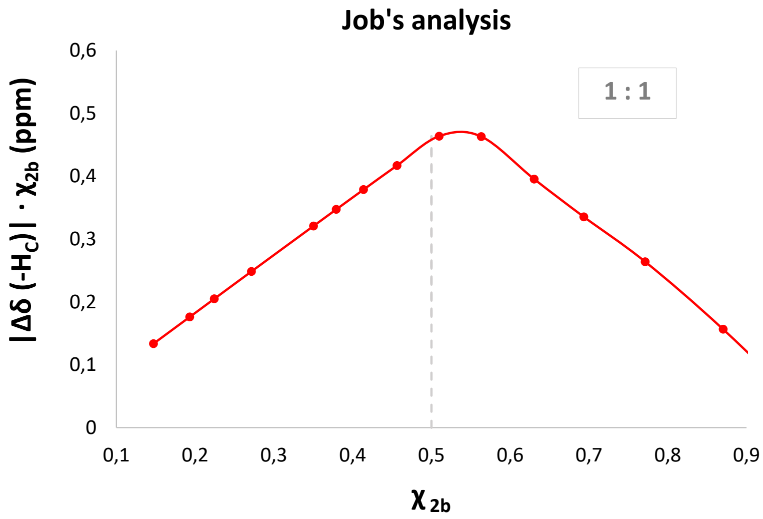

Figure S17. Stacked amplified ^{19}F NMR spectra (400 MHz) for the titration of rotaxane **2b** with TBAF·3H_2O in DCM-d_2 at 298 K (left); Job's plot for Δδ (H_C) signal for titration of rotaxane **2b** with TBAF·3H_2O in DCM-d_2 at 298 K. A maximum close to the 0.5 molar fraction of rotaxane **2b** points towards the 1:1 stoichiometry in a **2b:F**$^{-}$ complex.

Rotaxane 2c

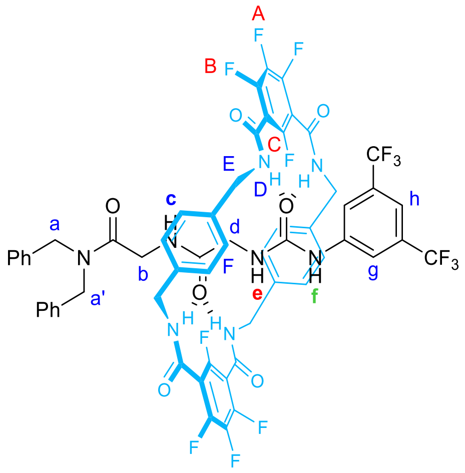

2c

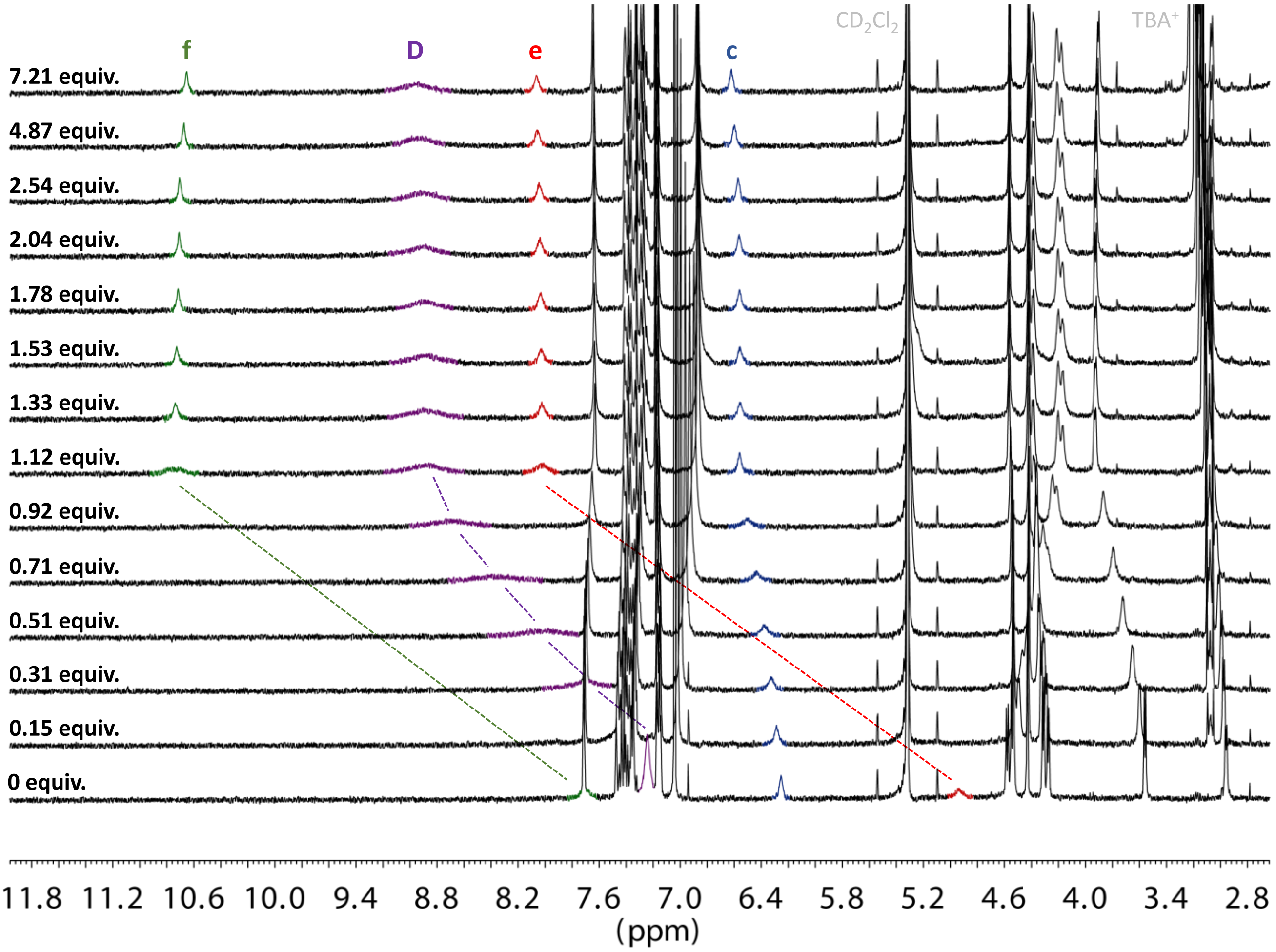

Figure S18. Stacked partial ^{1}H NMR spectra (400 MHz) for the titration of rotaxane **2c** with TBAF·3H_2O in DCM-d_2 at 298 K. Easy to see the shifting of NHs signals.

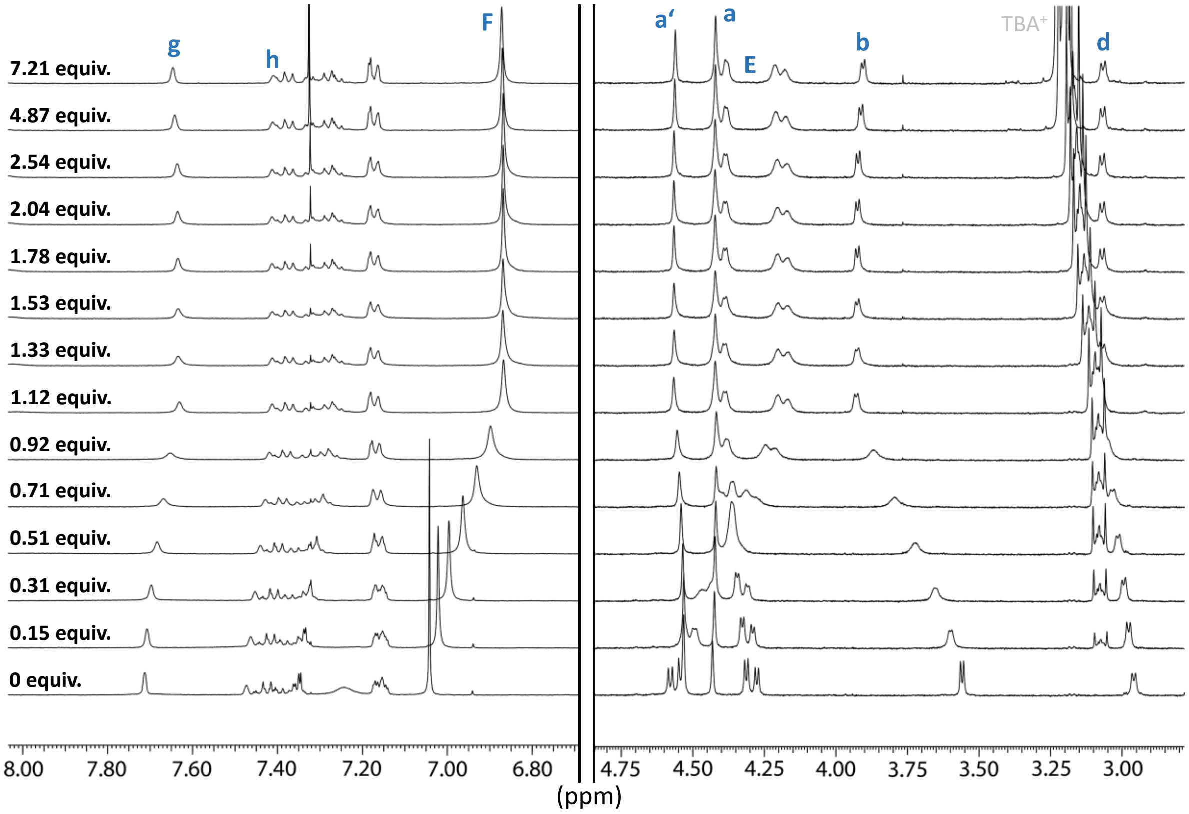

Figure S19. Stacked amplified ^{1}H NMR spectra (400 MHz) for the titration of rotaxane **2c** with TBAF·3H_2O in DCM-d_2 at 298 K. Easy to see the shifting of aromatic (left) and methylenes (right) signals.

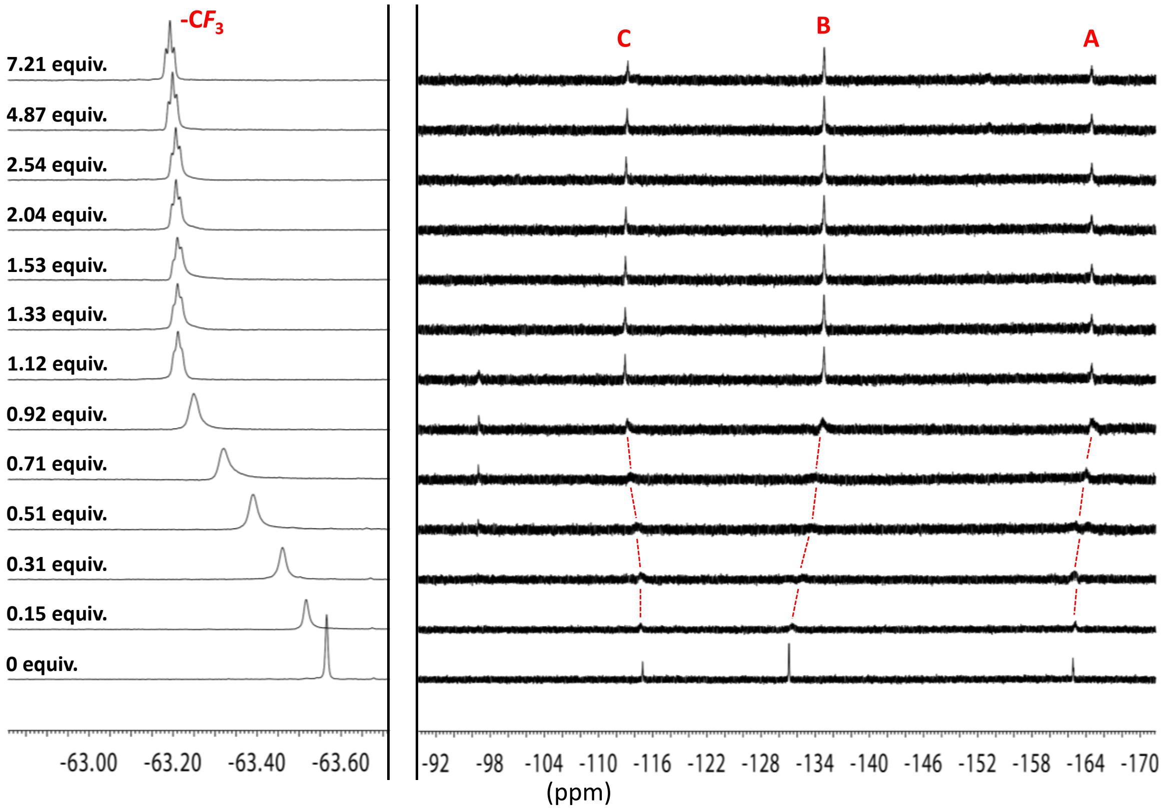

Figure S20. Stacked amplified ^{19}F NMR spectra (400 MHz) for the titration of rotaxane **2c** with TBAF·3H_2O in DCM-d_2 at 298 K. Signals related with -CF_3 (left) and with aromatic fluorines (right).

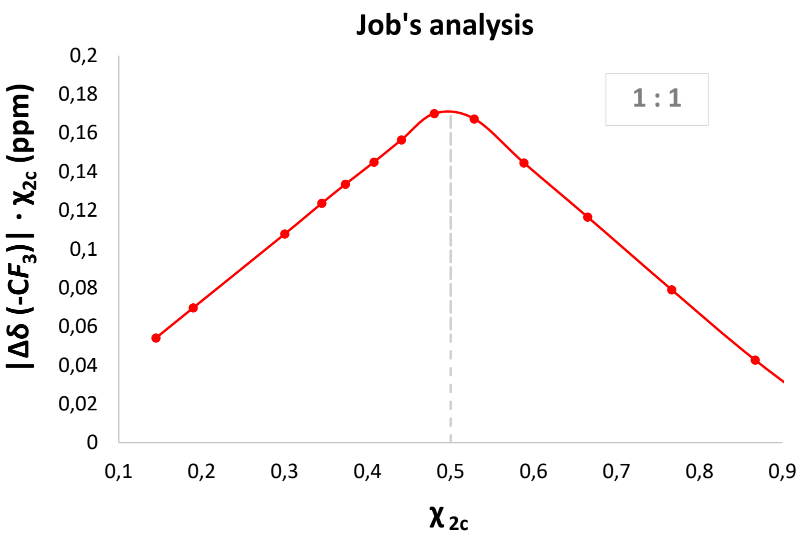

Figure S21. Job's plots for Δδ -C*F*$_3$ signals for titration of rotaxane **2c** with TBAF·3H_2O in DCM-d$_2$ at 298 K. A maximum at the 0.5 molar fraction of rotaxane **2c** points towards the 1:1 stoichiometry in a **2c:F**$^-$ complex.

9. NMR study of the position of the macrocycle in the complex rotaxane 2c:F$^-$

An approximately equimolar mixture of rotaxane **2c** and TBAF·3H_2O was dissolved in DCM-d_2 and the solution was placed in an NMR tube. ^{1}H-^{1}H NOESY and ^{19}F-^{1}H HOESY experiments were conducted to obtain valuable information about the positioning and interaction of the macrocycle with both the thread and the fluoride anion within the complex **2c:F$^-$**.

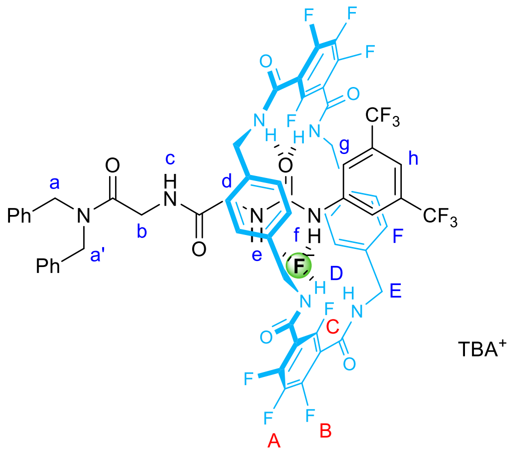

2c:F$^-$

^{1}H-^{1}H NOESY experiment (400 MHz, CD_2Cl_2, 298 K)

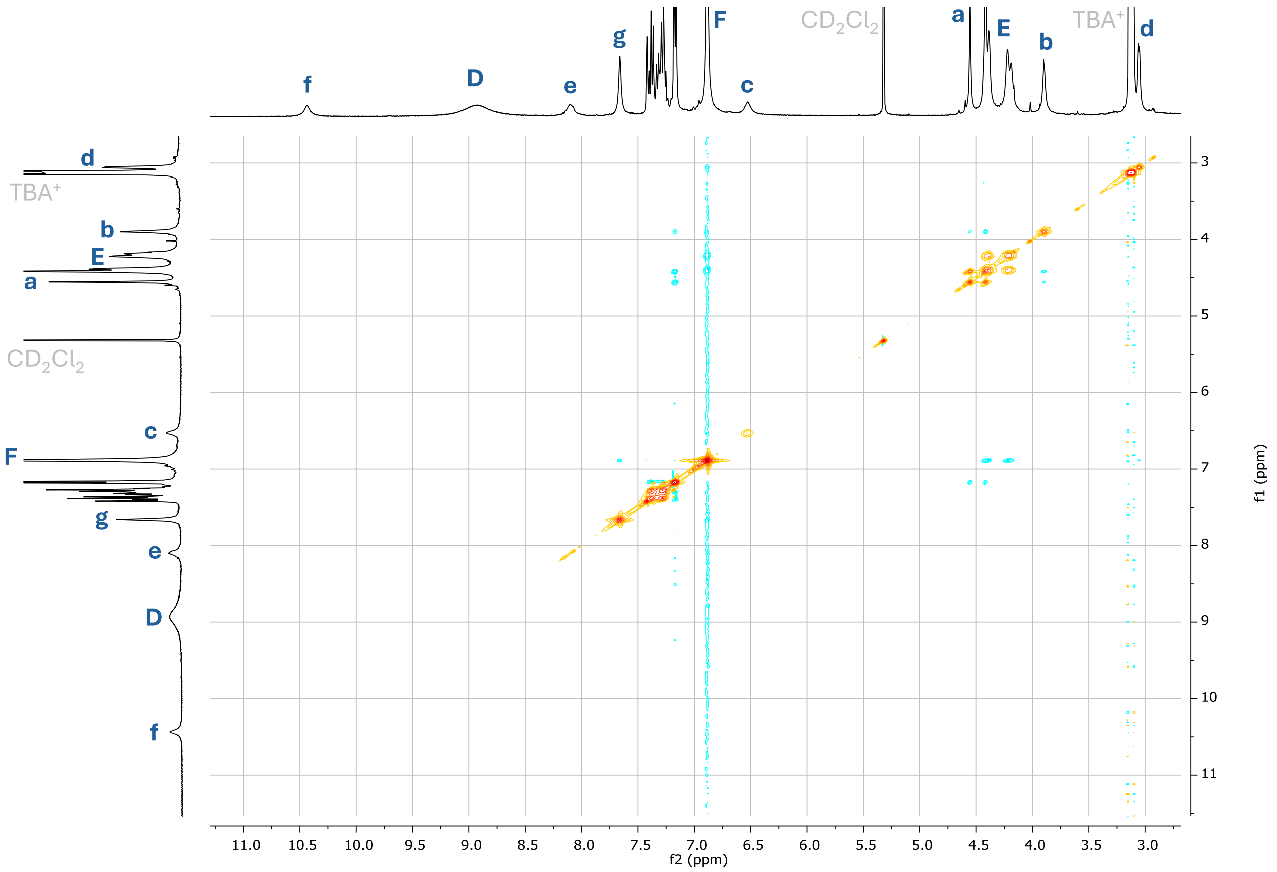

Figure S22. Two-dimensional spectrum of the ^{1}H-^{1}H NOESY experiment for the complex **2c:F$^-$** in DCM-d_2.

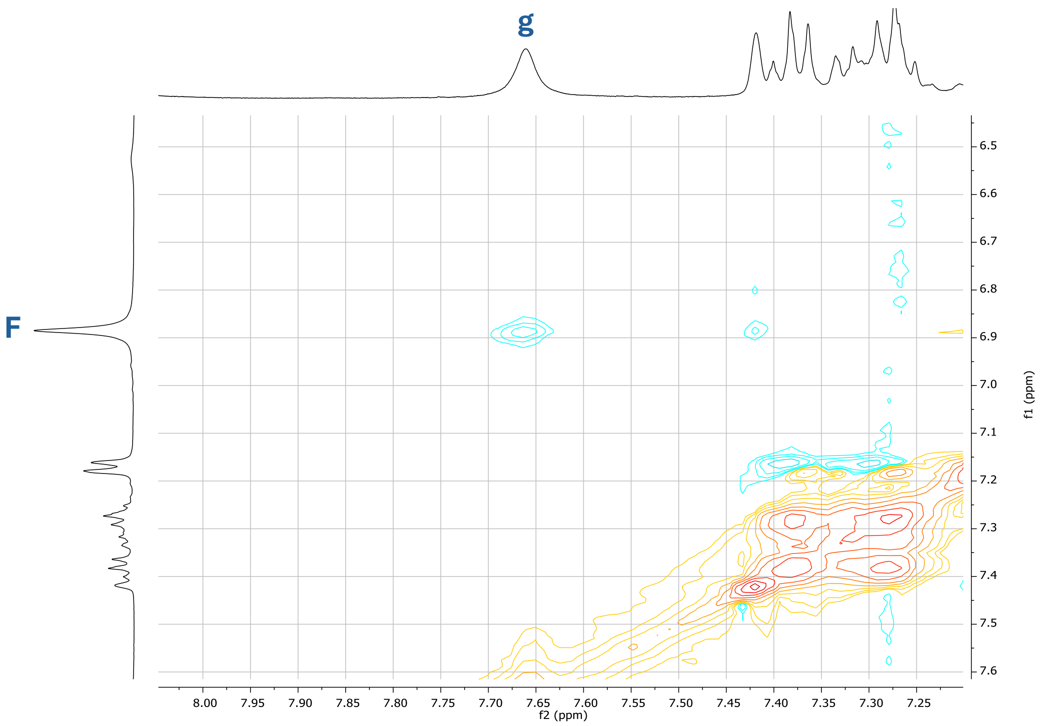

Figure S23. Amplified two-dimensional spectrum of the ^{1}H-^{1}H NOESY experiment for the complex **2c:F$^-$** in DCM-d_2. Easy to see the cross peak between H_F and H_g.

^{19}F-^{1}H HOESY experiment (400 MHz, CD_2Cl_2, 298 K)

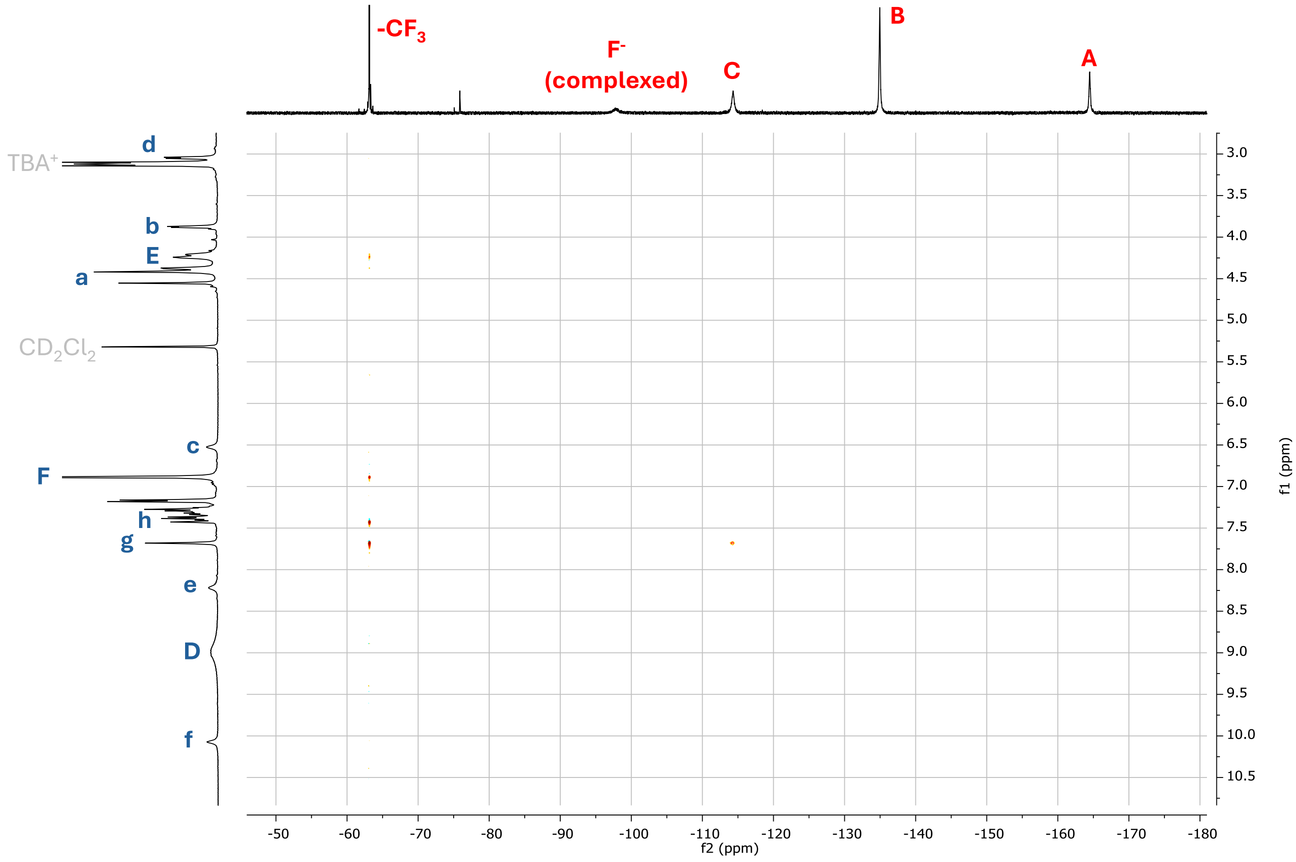

Figure S24. Two-dimensional spectrum of the ^{19}F-^{1}H HOESY experiment for the complex **2c:F$^-$** in DCM-d_2.

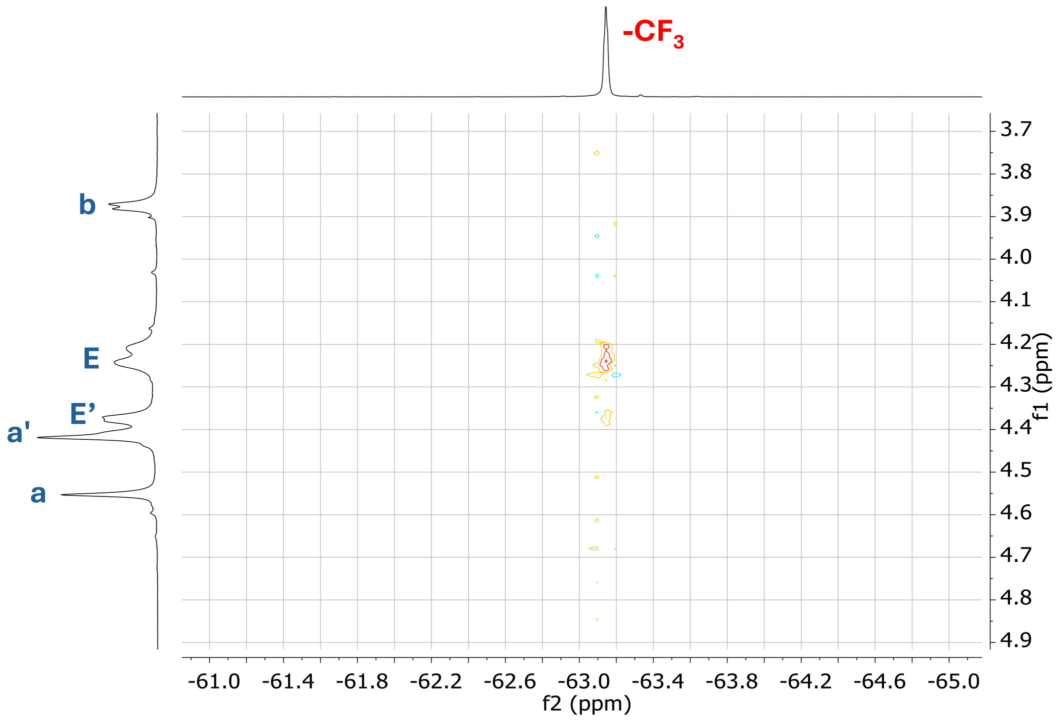

Figure S25. Amplified two-dimensional spectrum of the ^{19}F-^{1}H HOESY experiment for the complex **2c:F**$^{-}$ in DCM-d_2. Easy to see the cross peak between CF_3 and H_E.

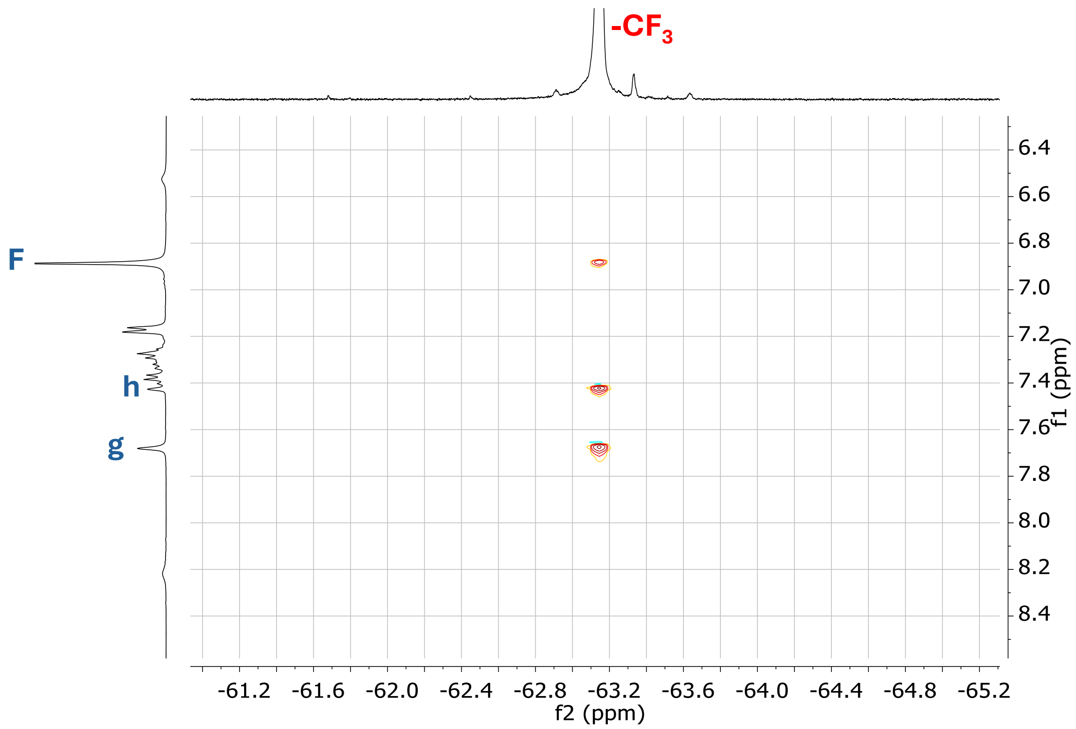

Figure S26. Amplified two-dimensional spectrum of the ^{19}F-^{1}H HOESY experiment for the complex **2c:F**$^{-}$ in DCM-d_2. Easy to see the cross peak between CF_3 and H_F.

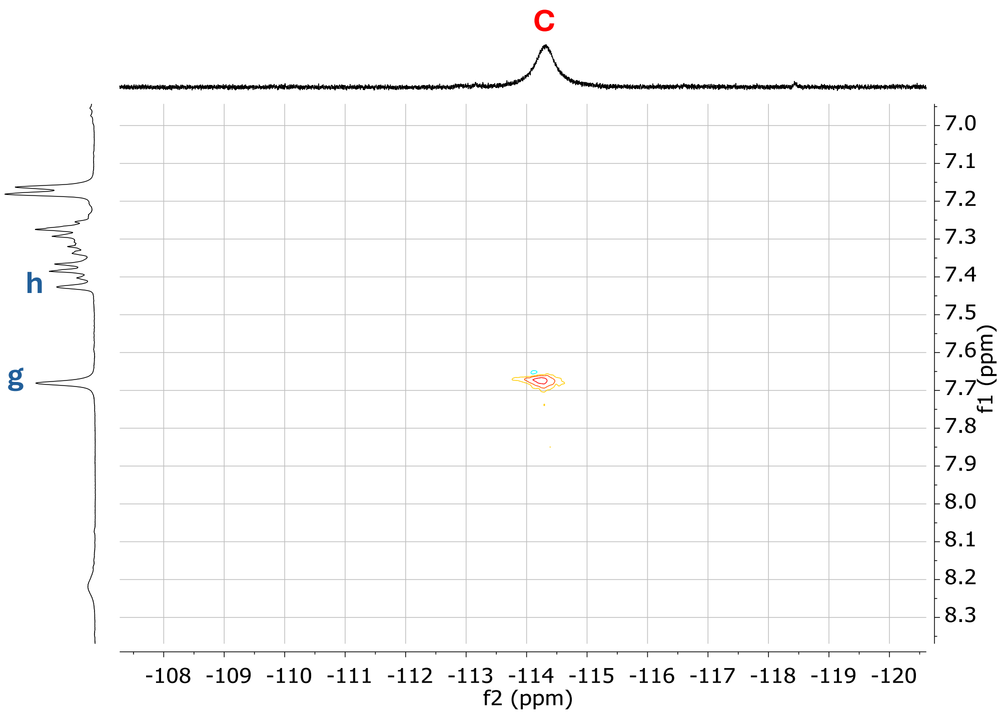

Figure S27. Amplified two-dimensional spectrum of the ^{19}F-^{1}H HOESY experiment for the complex **2c:F**$^{-}$ in DCM-d_2. Easy to see the cross peak between F_C and H_g.

10. Computational studies

Geometries of the molecules were optimized by using the PBEh-3c composite method.[11] The nature of minimum and transition structures of all stationary points on the potential energy surface was confirmed by frequency analysis at the same level of theory. Single-point energy calculations were performed by using the wB97M-V[12] functional and the ma-def2-TZVPP[13] basis set for all the atoms except in the case of the C atom that the def2-TZVP[14] basis set was applied. The computed thermochemical corrections at PBEh-3c level were combined with single-point energy calculations at the SMD(DCM)/wB97M-V/def2-tzvp [C] /ma-def2-TZVPP(all atoms)//PBEh-3c level to yield Gibbs free energies G at 298.15 K ($G_{298.sol}$). Solvent effects were calculated with the SMD solvation model with dichloromethane parameters.[15] To compute the complexation energy of all the complexes, the counterpoise method for three bodies at the M062X/aug-cc-PVDZ theoretical level was used.[16] The program ORCA 5.0 was used in the optimization stage.[17] Single-point energy and counterpoise calculations were performed by using the program Gaussian 16 C. 01.[18]

Rotaxanes 2b and 2c

There are structural differences between the lowest energy conformers of rotaxanes **2b** and **2c**. In the case of the rotaxane **2b**, the macrocycle is notably distorted to enhance the interactions with the thread via hydrogen bond. As is shown in the Figure S28, the NH groups of the urea motif are interacting with one carbonylic oxygen of the macrocycle while the second carbonyl group at the thread is forming a hydrogen bond with one NH group of the amide at the macrocycle. For fluorinated rotaxane **2c**, similar interactions commented for **2b** are present. In addition, the interaction between thread and macrocycle is reinforced by the interaction between the ortho-disubstituted F atom of the isophthalamide ring and one NH of the urea motif.

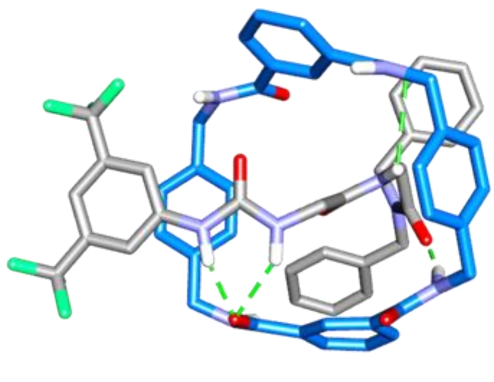

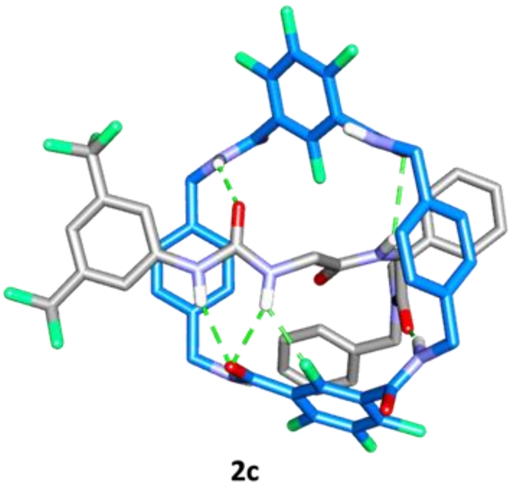

Figure S28. Optimized rotaxanes **2b** and **2c**.

Complexes with fluoride

Computational calculations predict that the complex **2b:F$^-$** involves a cooperative-tridentate binding mode between the rotaxane and the fluoride atom (Figure S29). The carbonyl group of the urea is interacting with both NH groups of one isophthalamide ring, therefore enhancing the acid character of the urea protons. In this case, the fluoride atom is gripped by the urea motif (dF$^-$-H = 1.485 Å and dF$^-$-H = 1.716 Å) and one amide NH of the macrocycle (dF$^-$-H = 1.576 Å).

On other hand, **2c:F$^-$** complex presents a cooperative-bidentate binding mode in which the fluorinated rotaxane holds the fluoride atom involving the most acidic NH of the urea system (dF$^-$-H = 1.358 Å) and one NH of the isophthalamide moiety (dF$^-$-H = 1.498 Å). Besides of the ortho-disubstituted fluor atom placed at the isophthalamide ring is directly interacting with the free NH of the urea fragment enhancing thus the stability of the complex (dF$^-$-H = 2.146 Å).

Simulations also exposed that, in both complexes **2b:F$^-$** and **2c:F$^-$**, the urea NH groups directly interacting with the fluoride present an elongation of the N-H bond respect to they are at the isolated rotaxane. For instance the computed ArN-H bond distance at **2b** is of 1.012 Å while that distance at **2b:F$^-$** is 1.062 Å. In the case of fluorinated rotaxane: dArN-H at **2c** is 1.011 Å and at **2c:F$^-$** is 1.105 Å.

Calculations also predict that the complexation energy of **2c:F$^-$** is higher than that of **2b:F$^-$** (–148.9 vs –142.5 kJ mol^{-1}) supporting that the capability of catalyst **2c** to induce the fluoride phase-transfer is higher than for **2b**.

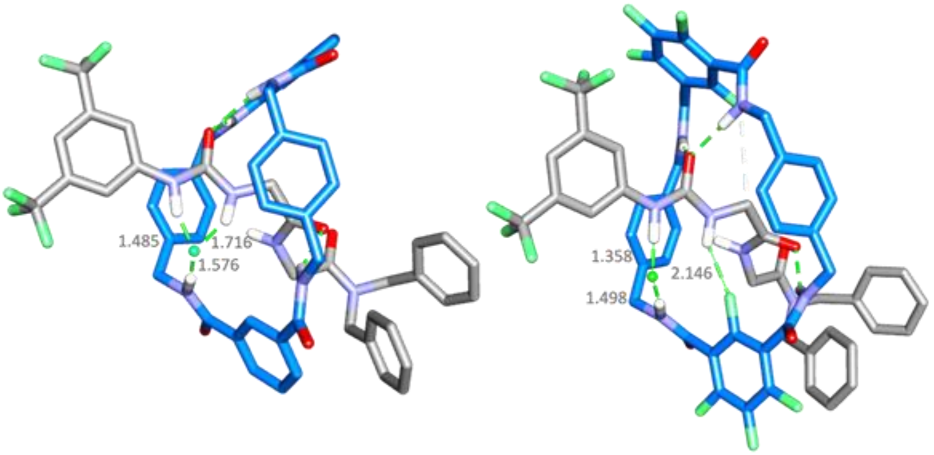

2b:F$^-$
(ΔE_{comp} = –142.5 kJ mol^{-1})

2c:F$^-$
(ΔE_{comp} = –148.9 kJ mol^{-1})

Figure S29. Optimized rotaxane complexes **2b:F$^-$** and **2c:F$^-$**.

In this sense, we also calculated the complexation energy of the complex formed by two molecules of thread **1b** and fluoride atom **(1b)$_2$:F$^-$**, being that of –140.8 kJ mol^{-1}, that is, much lower than that computed for the rotaxane complex **2c:F$^-$** (Figure S30).

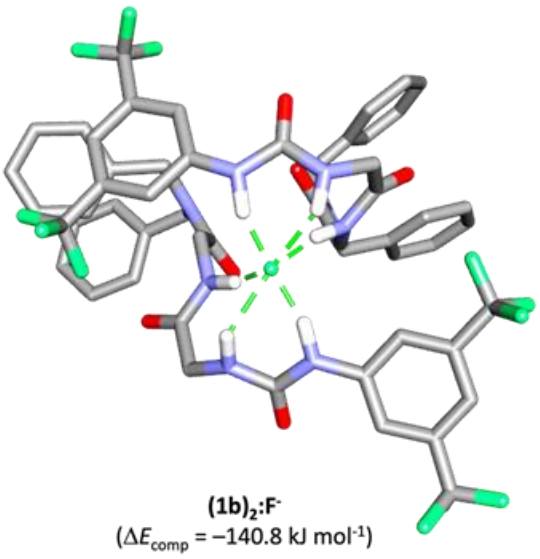

Figure S30. Optimized thread complex (**1b)$_2$:F$^-$**.

Computational data

Table S7. Electronic ($E_{SCF.298.sol}$), Gibbs free ($G_{298.sol}$) energies for the located conformer of the minima systems shown in Figures S28, S29 and S30 (in Hartree) computed at SMD(DCM)/wB97M-V/def2-tzvp [C] /ma-def2-TZVPP(all atoms)//PBEh-3c level. The PBEh-3c level of theory was used to optimize the geometries and calculate the thermal corrections.

Filename	E_{tot} PBEh-3c	G_{298} PBEh-3c	$E_{tot.sol}$ SMD/wB97M-V/ def2-tzvp [C]/ma-def2-TZVPP(all atoms)//PBEh-3c	$G_{298.sol}$ SMD/wB97M-V/ def2-tzvp [C]/ma-def2-TZVPP(all atoms)//PBEh-3c
F^-	-99.5881411	-99.6022996	-99.9947731	-100.0089317
**1b**				
1b_01	-2082.8006843	-2082.3608968	-2087.4244729	-2086.9846854
**(1b)$_2$:F$^-$**				
eje2_F_01	-4265.4413164	-4264.5304690	-4274.9192748	-4274.0084274
eje2_F_02	-4265.4206629	-4264.5138035	-4274.9021427	-4273.9952833
eje2_F_03_freq	-4265.4293599	-4264.5212233	-4274.9036316	-4273.9954951
eje2_F_04_freq	-4265.4208540	-4264.5134558	-4274.9032288	-4273.9958306
**2b**				
rtxH_01	-3835.3338976	-3834.3537444	-3843.8824754	-3842.9023222
rtxH_02	-3835.3249706	-3834.3478998	-3843.8775907	-3842.9005200
rtxH_03	-3835.3253452	-3834.3504566	-3843.8761290	-3842.9012404
**2b:F$^-$**				
rtxH_F_01_a	-3935.0982778	-3934.1229832	-3943.8959220	-3942.9206274
rtxH_F_02_a	-3935.0705517	-3934.0976232	-3943.8817986	-3942.9088701
rtxH_F_03_a	-3935.1048493	-3934.1288200	-3943.9009132	-3942.9248839
rtxH_F_04_freq	-3935.0987425	-3934.1226159	-3943.9002498	-3942.9241231
rtxH_F_05	-3935.0856530	-3934.1091508	-3943.8901204	-3942.9136182
**2c**				
rtxF_01	-4627.6968108	-4626.7945462	-4637.9588544	-4637.0565898
rtxF_02_a	-4627.6901635	-4626.7848217	-4637.9564042	-4637.0510624

rtxF_03_a_freq	-4627.6862336	-4626.7835057	-4637.9555152	-4637.0527873
**2c:F⁻**				
rtxF_F_01_a	-4727.4561124	-4726.5564733	-4737.9730285	-4737.0733894
rtxF_F_02_a	-4727.4394267	-4726.5383808	-4737.9608784	-4737.0598325
rtxF_F_03_a	-4727.4628887	-4726.5620456	-4737.9803800	-4737.0795369
rtxF_F_04_freq	-4727.4627229	-4726.5595063	-4737.9817082	-4737.0784916
rtxF_F_05	-4727.4489763	-4726.5470069	-4737.9705128	-4737.0685435

Cartesian Coordinates

(the lowest energy conformer is shown)

$(1b)_2$:F^- (eje2_F_04_DCM)

F 1.808173 -1.982829 -5.103989
F 1.811503 -4.070526 -4.584566
F 3.617241 -2.916936 -4.421191
F -1.726311 -2.899347 -0.438211
F -0.390479 -3.397335 1.175027
F -0.629805 -4.745007 -0.484202
O 9.486712 1.167520 1.582337
O 6.685037 2.152918 2.262295
O 4.673448 0.479430 -1.574449
N 9.185883 -0.183118 3.374581
N 6.942705 0.609974 0.634282
H 6.637899 0.309496 -0.278369
N 4.178765 1.765803 0.238214
H 3.619972 1.766436 1.091924
N 2.806763 0.051776 -0.290143
H 2.397993 0.330015 0.643566
C 7.217248 -1.115445 4.690446
C 6.566604 -2.239164 5.196016
H 7.023981 -3.217891 5.096897
C -0.538934 -3.449421 -0.146046
C 2.296090 -2.876681 -4.223862
C 5.330535 -2.127709 5.808071
H 4.836905 -3.011991 6.185605
C 4.715970 -0.886654 5.907652
H 3.734838 -0.800614 6.355828
C 5.356553 0.232622 5.404733
H 4.880623 1.202599 5.459544
C 6.605651 0.122980 4.806263
H 7.075644 1.005258 4.400242
C 8.544996 -1.326763 3.992284
H 9.256723 -1.731218 4.716608
H 8.418856 -2.121500 3.252268
C 10.291315 0.437414 4.084031
H 10.910225 0.955126 3.353196
H 10.908835 -0.348286 4.527926
C 9.838756 1.397617 5.153495
C 9.995895 1.097802 6.499871
H 10.466047 0.164797 6.789827
C 9.546820 1.972863 7.480209
H 9.669384 1.720490 8.525643
C 8.931826 3.159816 7.118389
H 8.572934 3.840661 7.879436
C 8.769578 3.467138 5.773298
H 8.280725 4.387529 5.481671
C 9.221852 2.595734 4.797348

H	9.072146	2.831572	3.751212
C	8.847407	0.305290	2.143262
C	7.625429	-0.326171	1.480039
H	7.969522	-1.156206	0.859925
H	6.936770	-0.742025	2.216830
C	6.433723	1.753131	1.142539
C	5.435852	2.463713	0.236994
H	5.288034	3.472237	0.620782
H	5.805553	2.540166	-0.785842
C	3.945224	0.737804	-0.623930
C	2.245953	-1.012831	-0.951615
C	2.605155	-1.438638	-2.233143
H	3.400492	-0.930575	-2.756176
C	1.937492	-2.508007	-2.816757
C	0.924982	-3.183008	-2.154978
H	0.418001	-4.017737	-2.615983
C	0.578801	-2.763325	-0.875073
C	1.216788	-1.692265	-0.283345
H	0.925932	-1.355691	0.703767
H	2.374816	-0.462546	2.860003
C	0.790847	-1.125680	3.958807
O	0.182213	-1.980481	4.575998
N	0.308479	0.130643	3.718301
N	2.055010	-1.275733	3.433469
H	0.854934	0.689893	3.057835
C	-1.081823	0.399304	3.832455
C	2.785944	-2.435837	3.374410
C	-1.808743	0.297080	2.496118
H	-1.241267	1.408150	4.229558
H	-1.531108	-0.296976	4.543512
C	2.589817	-3.537367	4.209927
C	3.800597	-2.501130	2.409859
O	-1.274139	-0.090090	1.482636
N	-3.120883	0.678883	2.530279
H	1.803641	-3.504760	4.947433
C	3.399890	-4.656021	4.078657
C	4.579188	-3.632915	2.281125
H	3.946710	-1.652361	1.754197
H	-3.394439	1.248357	3.314507
C	-3.762551	0.993789	1.285168
C	3.236059	-5.780424	5.052527
C	4.397655	-4.724106	3.122154
C	5.632602	-3.726997	1.222478
C	-3.298572	2.370574	0.787477
H	-4.846236	1.012115	1.431196
H	-3.548563	0.192481	0.581688
F	3.931636	-5.547294	6.181211
F	3.682444	-6.947918	4.571846
F	1.965570	-5.966140	5.417690
H	5.006724	-5.610716	3.016810
F	6.872539	-3.683476	1.750281
F	5.560400	-2.745771	0.321676
F	5.561730	-4.884230	0.553288
O	-2.904293	3.183162	1.602068
N	-3.352088	2.632888	-0.540489
C	-3.938355	1.765950	-1.541717
C	-2.794402	3.876099	-1.045159
C	-2.923912	1.225214	-2.518567
H	-4.703257	2.330126	-2.085632
H	-4.470077	0.948823	-1.055941
H	-2.130314	3.641232	-1.881006
H	-2.178989	4.314324	-0.262163

C	-3.854636	4.850641	-1.484871
C	-3.096003	1.417688	-3.883868
C	-1.801692	0.537326	-2.065813
C	-4.685215	5.454672	-0.543487
C	-4.028894	5.157479	-2.828710
H	-3.964446	1.960090	-4.241918
C	-2.166209	0.926276	-4.790720
C	-0.873242	0.048314	-2.970456
H	-1.636467	0.385178	-1.005245
H	-4.547265	5.227788	0.506845
C	-5.664975	6.349040	-0.942003
C	-5.009786	6.054867	-3.231180
H	-3.385249	4.693020	-3.567266
H	-2.310246	1.085949	-5.851646
C	-1.052497	0.239989	-4.333719
H	-0.003236	-0.480579	-2.608264
H	-6.299091	6.818083	-0.200532
C	-5.830740	6.651314	-2.287954
H	-5.130621	6.286792	-4.281536
H	-0.320666	-0.146912	-5.029958
H	-6.594713	7.352725	-2.597318
F	2.303790	0.872036	2.041295

2b (rtxH_01_DCM)

F	7.069037	5.199593	12.442008
F	7.436514	4.127618	14.271606
F	6.421126	3.158864	12.645888
F	11.100419	0.855566	14.513364
F	12.513339	0.747675	12.892794
F	10.678704	-0.365677	12.795156
O	10.421860	4.340187	2.341464
O	9.721738	4.140781	5.629296
O	9.535658	5.608044	8.823661
N	8.463202	3.267188	2.653002
N	9.732981	6.052081	4.464786
H	10.048879	7.003518	4.377390
N	11.386663	5.124019	7.604281
H	12.118254	4.448934	7.460249
N	11.019741	4.008062	9.547566
H	11.794435	3.441004	9.225627
C	6.191168	4.247168	2.370761
C	5.223455	4.958574	3.071778
H	5.134316	4.832787	4.145428
C	11.211512	0.803987	13.184132
C	7.418515	4.008066	12.942725
C	4.383760	5.842502	2.409662
H	3.642619	6.396174	2.970402
C	4.504170	6.027173	1.041288
H	3.852719	6.720438	0.525802
C	5.471794	5.324745	0.336831
H	5.574824	5.466248	-0.731200
C	6.310696	4.441510	0.998229
H	7.066452	3.905540	0.436318
C	7.077714	3.269160	3.102662
H	7.015462	3.440111	4.180579
H	6.699532	2.256287	2.951084
C	8.954091	2.072381	2.003195
H	9.923119	2.303917	1.563770
H	8.286536	1.816298	1.172929
C	9.080012	0.865086	2.904872
C	9.350006	-0.369690	2.317337

H	9.464665	-0.436609	1.240727
C	9.466811	-1.516273	3.084643
H	9.679913	-2.464013	2.607528
C	9.279009	-1.452371	4.460009
H	9.327551	-2.352544	5.059260
C	9.003108	-0.230029	5.051652
H	8.819915	-0.164387	6.117104
C	8.930476	0.927324	4.283809
H	8.759217	1.875827	4.774378
C	9.290582	4.328047	2.800971
C	8.760527	5.560267	3.529134
H	8.574223	6.339925	2.790271
H	7.813311	5.385329	4.033410
C	10.107283	5.287306	5.502493
C	11.043330	5.958426	6.501097
H	11.960780	6.256976	5.989292
H	10.577154	6.877428	6.861975
C	10.547811	4.955501	8.663239
C	10.421713	3.548179	10.701829
C	9.247500	4.069219	11.245714
H	8.775121	4.907331	10.761416
C	8.723394	3.510793	12.399586
C	9.343364	2.451826	13.045637
H	8.922773	2.030987	13.948695
C	10.529694	1.962490	12.520796
C	11.061680	2.499721	11.364963
H	11.992048	2.119745	10.961352
H	7.010419	6.475349	5.878355
H	7.188878	10.196506	7.991208
H	6.113883	9.141725	9.928364
H	5.539690	6.756408	9.886367
H	13.507531	3.695222	6.191004
H	12.149251	-0.119427	4.758639
H	12.392169	0.663933	2.480361
H	13.038712	2.994772	2.010810
O	7.611905	8.606711	4.482295
O	5.618006	4.464828	6.757853
O	12.873798	2.152488	8.000003
O	14.450826	5.504406	4.598547
N	8.787806	9.946389	5.860398
H	9.115959	10.067634	6.802499
N	6.098562	4.313976	8.970285
H	6.613873	4.771769	9.703469
N	11.721967	0.331111	7.322202
H	11.275592	-0.089364	6.522929
N	13.064939	5.521069	2.797672
H	12.177260	5.131396	2.491863
C	7.873665	8.975656	5.609820
C	7.217149	8.388959	6.816084
C	6.841596	7.053869	6.775704
C	6.276289	6.445294	7.886617
C	6.019904	7.202918	9.024703
C	6.343236	8.549772	9.052776
C	6.955338	9.139343	7.959789
C	5.959993	4.985253	7.803941
C	5.967468	2.878847	9.084500
H	5.290277	2.544881	8.298207
H	5.489645	2.648275	10.037772
C	7.289696	2.159226	8.977701
C	8.176397	2.486199	7.962095
H	7.940517	3.255446	7.241855
C	9.387623	1.830605	7.839989

H	10.034842	2.139052	7.031791
C	9.747492	0.821070	8.721913
C	8.864163	0.496451	9.746442
H	9.124670	-0.271095	10.465715
C	7.654205	1.162228	9.875940
H	6.990033	0.900261	10.690932
C	11.067349	0.090545	8.586139
H	11.750132	0.368418	9.389159
H	10.908728	-0.985179	8.685544
C	12.475349	1.427628	7.095691
C	12.742401	1.761453	5.663559
C	13.200450	3.043476	5.385165
C	13.266015	3.513810	4.084070
C	12.966145	2.652831	3.033927
C	12.599786	1.343736	3.295069
C	12.465440	0.904035	4.600496
C	13.660182	4.940668	3.863289
C	13.359432	6.880181	2.405453
H	13.314461	6.935652	1.315673
H	14.383870	7.106429	2.697005
C	12.404458	7.880863	3.002638
C	12.614678	8.399592	4.277859
H	13.501273	8.110094	4.828328
C	11.695897	9.271475	4.841080
H	11.883651	9.668731	5.832677
C	10.546370	9.646270	4.150124
C	10.342714	9.135185	2.873754
H	9.448651	9.406053	2.327388
C	11.264097	8.266107	2.307653
H	11.083332	7.871718	1.314536
C	9.535832	10.567433	4.787777
H	10.037436	11.441304	5.206547
H	8.837623	10.936650	4.035843

2b:F⁻ (rtxH_F_03_a_DCM)

F	-6.584952	-0.585524	3.555888
F	-7.623975	-2.466760	3.611977
F	-7.507397	-1.356153	1.772529
F	-4.419870	-6.160827	3.941894
F	-2.786676	-6.265365	2.543378
F	-4.810494	-6.564750	1.872787
O	1.146260	1.689253	-4.455863
O	1.760122	1.366656	-1.370524
O	-3.075818	0.388795	0.375429
N	3.251055	0.956713	-4.838023
N	0.638902	-0.182550	-2.555843
H	-0.246693	-0.635167	-2.722006
N	-1.019369	-0.324228	-0.319035
H	-0.355788	-1.095915	-0.165822
N	-2.245463	-1.675554	0.979943
H	-1.352507	-2.240979	0.872921
C	5.340030	0.198209	-3.676343
C	6.647367	-0.200106	-3.924074
H	6.900277	-0.646084	-4.880127
C	-4.041706	-5.839574	2.698219
C	-6.815055	-1.706781	2.863939
C	7.637322	-0.029697	-2.966920
H	8.651452	-0.344971	-3.177555
C	7.326228	0.550291	-1.746924
H	8.092077	0.685638	-0.994624
C	6.022916	0.954329	-1.495711

H	5.769372	1.409588	-0.547628
C	5.034498	0.776869	-2.449940
H	4.027134	1.105586	-2.230756
C	4.279195	-0.053000	-4.724554
H	4.762607	-0.145537	-5.702475
H	3.822765	-1.027929	-4.543603
C	3.627643	2.199418	-5.493250
H	2.719794	2.682719	-5.849589
H	4.232280	1.951572	-6.370195
C	4.395751	3.121448	-4.579559
C	5.765997	3.290988	-4.725026
H	6.285415	2.786031	-5.531245
C	6.484278	4.069593	-3.829735
H	7.554931	4.176233	-3.946031
C	5.831968	4.692283	-2.777390
H	6.390310	5.291976	-2.070080
C	4.460067	4.538058	-2.631239
H	3.942537	5.016416	-1.810022
C	3.745190	3.759566	-3.526533
H	2.679277	3.627337	-3.391862
C	2.006113	0.851079	-4.294637
C	1.729088	-0.399161	-3.456561
H	1.477388	-1.237243	-4.111433
H	2.612455	-0.706167	-2.896831
C	0.733834	0.743424	-1.587724
C	-0.522502	0.950047	-0.763360
H	-0.272451	1.618590	0.063950
H	-1.259505	1.454212	-1.395225
C	-2.184164	-0.466269	0.343487
C	-3.325157	-2.286682	1.563994
C	-4.531455	-1.669182	1.897576
H	-4.665901	-0.618172	1.691880
C	-5.539527	-2.409842	2.506531
C	-5.391756	-3.757393	2.776137
H	-6.183373	-4.320808	3.248335
C	-4.190083	-4.371782	2.437939
C	-3.172377	-3.654057	1.848803
H	-2.231450	-4.125968	1.594072
H	-3.107905	2.520932	-0.487924
H	-2.779109	6.684483	0.341015
H	-3.789889	7.194123	-1.860309
H	-4.459125	5.353775	-3.371329
H	1.979651	-2.432881	-0.367944
H	3.478085	-4.215747	-3.960375
H	5.769610	-3.515860	-3.344973
H	6.149934	-2.227954	-1.268502
O	-1.751712	5.238645	2.032470
O	-4.714597	3.050946	-4.086489
O	1.095582	-4.179533	-3.868343
O	5.325718	-1.620351	1.210731
N	-2.359828	3.067553	1.910916
H	-2.749623	2.318025	1.359168
N	-4.118113	1.464227	-2.602727
H	-3.837013	1.229737	-1.662415
N	0.195581	-3.765142	-1.838201
H	0.252977	-3.359782	-0.879541
N	3.431578	-0.462263	0.769591
H	2.820745	-0.159898	0.026491
C	-2.279014	4.323789	1.423641
C	-2.903206	4.566822	0.080125
C	-3.278243	3.544789	-0.783158
C	-3.839602	3.813272	-2.025967

C	-4.024554	5.138952	-2.404918
C	-3.647841	6.165580	-1.555185
C	-3.085117	5.886207	-0.321021
C	-4.264421	2.748621	-2.996245
C	-4.537171	0.358662	-3.439270
H	-4.452521	0.667023	-4.480226
H	-5.593443	0.125360	-3.265382
C	-3.691560	-0.855558	-3.171582
C	-2.558584	-1.122837	-3.936701
H	-2.307030	-0.469028	-4.763898
C	-1.754576	-2.217053	-3.658067
H	-0.898191	-2.434165	-4.286782
C	-2.044650	-3.058231	-2.584507
C	-3.190893	-2.807711	-1.844894
H	-3.454118	-3.463927	-1.023770
C	-4.006293	-1.723232	-2.135416
H	-4.890148	-1.547943	-1.532594
C	-1.125463	-4.200683	-2.228059
H	-1.557598	-4.759131	-1.394601
H	-1.036438	-4.889599	-3.067961
C	1.216927	-3.809021	-2.704791
C	2.572532	-3.377660	-2.216495
C	2.795885	-2.655962	-1.045440
C	4.079848	-2.228802	-0.717587
C	5.154569	-2.557872	-1.533926
C	4.936670	-3.276083	-2.696781
C	3.655011	-3.672351	-3.042408
C	4.350249	-1.421627	0.512644
C	3.518829	0.403208	1.920011
H	4.238729	1.213183	1.755010
H	3.905008	-0.182522	2.756059
C	2.170542	0.981854	2.258665
C	1.066981	0.155620	2.449118
H	1.156083	-0.915946	2.313243
C	-0.166849	0.700972	2.768850
H	-1.014309	0.041972	2.918622
C	-0.336601	2.076314	2.886258
C	0.766167	2.901021	2.691809
H	0.647435	3.975310	2.759687
C	2.002672	2.355833	2.384856
H	2.849096	3.012624	2.222685
C	-1.702078	2.665474	3.136054
H	-2.339491	1.931883	3.633397
H	-1.635971	3.533612	3.790103
F	0.031609	-2.629157	0.499157

2c (rtxF_01_DCM)

F	8.035027	5.882714	13.388516
F	8.314521	4.517116	15.028222
F	6.894103	4.062812	13.480356
F	11.387923	0.838924	14.647162
F	12.608810	0.673519	12.881479
F	10.680821	-0.277972	12.953305
O	10.617075	4.143741	2.484807
O	9.633651	3.990611	5.538947
O	9.404613	5.646296	9.093479
N	8.605461	3.147330	2.354881
N	9.665123	5.912329	4.392704
H	9.863779	6.899711	4.384821
N	11.059980	5.030530	7.664789
H	11.788519	4.360140	7.482624

N	10.876446	3.972912	9.655937
H	11.560016	3.349627	9.247164
C	6.488953	4.277831	1.714854
C	5.407890	4.964731	2.253575
H	5.063826	4.728657	3.254209
C	11.353883	0.826393	13.313543
C	8.106673	4.589884	13.711526
C	4.774745	5.966368	1.533032
H	3.942672	6.499803	1.972624
C	5.218837	6.292878	0.261284
H	4.732329	7.079050	-0.300434
C	6.296164	5.610269	-0.285296
H	6.648134	5.859060	-1.277926
C	6.927924	4.609334	0.436886
H	7.774661	4.091202	0.002888
C	7.161199	3.190154	2.517585
H	6.887857	3.279085	3.572802
H	6.778313	2.216285	2.206090
C	9.178247	1.934015	1.809522
H	10.215943	2.131739	1.546079
H	8.662337	1.688292	0.876037
C	9.092693	0.747246	2.742939
C	9.286362	-0.530108	2.222478
H	9.501299	-0.652405	1.166963
C	9.194410	-1.649504	3.031948
H	9.347118	-2.633198	2.608103
C	8.886305	-1.510426	4.378504
H	8.781046	-2.385527	5.006521
C	8.698762	-0.242897	4.907172
H	8.437882	-0.117968	5.951322
C	8.819771	0.882304	4.098794
H	8.693480	1.864386	4.535204
C	9.417741	4.167010	2.713144
C	8.790712	5.385889	3.383549
H	8.634846	6.155330	2.625882
H	7.812853	5.172479	3.811876
C	9.947606	5.164104	5.471880
C	10.691279	5.886366	6.587667
H	11.585557	6.352547	6.169610
H	10.070141	6.701052	6.962245
C	10.356220	4.933224	8.818097
C	10.458347	3.616062	10.923420
C	9.512656	4.316432	11.671722
H	9.073306	5.215291	11.272146
C	9.166352	3.863451	12.935763
C	9.747053	2.732976	13.487738
H	9.474888	2.397320	14.478121
C	10.714099	2.061838	12.753940
C	11.065372	2.494152	11.490097
H	11.822266	1.966416	10.922292
F	7.296436	6.019928	6.564008
F	6.253209	10.467000	7.593356
F	4.516326	9.626336	9.472249
F	4.189776	6.985843	9.902803
F	13.257336	3.935598	5.886856
F	12.092410	-0.401599	4.384141
F	12.603962	0.353683	1.913022
F	13.484371	2.841202	1.394582
O	7.502092	8.434706	4.763574
O	4.586026	4.411999	7.987458
O	12.642902	2.096067	7.631848
O	15.055085	5.112446	3.718273

N	8.746111	9.522497	6.299177
H	8.866529	9.724166	7.276613
N	6.356771	4.466577	9.421621
H	7.176626	5.003871	9.664705
N	11.452659	0.339247	6.896205
H	11.024900	-0.113755	6.106632
N	13.027667	5.581418	2.789345
H	12.087391	5.210843	2.657650
C	7.720192	8.732857	5.916152
C	6.837445	8.259902	7.039096
C	6.624585	6.911684	7.285298
C	5.760943	6.451763	8.258319
C	5.047172	7.389954	8.979642
C	5.211814	8.745262	8.766748
C	6.108827	9.161031	7.800082
C	5.505805	4.986027	8.519768
C	6.301353	3.099100	9.878299
H	5.372259	2.664529	9.507430
H	6.241283	3.091757	10.969028
C	7.484866	2.283525	9.425972
C	8.100764	2.534952	8.209515
H	7.749514	3.330593	7.566646
C	9.204033	1.800670	7.810066
H	9.667257	2.060676	6.871266
C	9.712796	0.787490	8.606742
C	9.084987	0.519769	9.819765
H	9.472457	-0.254185	10.471914
C	7.989683	1.263234	10.225970
H	7.538546	1.059191	11.190083
C	10.937992	-0.000893	8.199711
H	11.732487	0.132292	8.935680
H	10.706948	-1.068006	8.189734
C	12.244900	1.409350	6.703503
C	12.609590	1.739036	5.281500
C	13.096187	3.006479	4.949473
C	13.399690	3.408501	3.664699
C	13.219685	2.500476	2.644393
C	12.768089	1.222545	2.904508
C	12.485504	0.857102	4.204774
C	13.931625	4.798421	3.397773
C	13.287243	6.948436	2.405947
H	13.161327	7.055929	1.325273
H	14.327786	7.176693	2.633079
C	12.356990	7.884597	3.131482
C	12.588621	8.213382	4.464442
H	13.485273	7.850937	4.954543
C	11.683771	8.991111	5.167617
H	11.884537	9.234753	6.204826
C	10.519239	9.455495	4.560532
C	10.299019	9.146612	3.223652
H	9.397473	9.494861	2.735916
C	11.211617	8.374364	2.517256
H	11.016268	8.134752	1.478617
C	9.522335	10.279615	5.334374
H	10.039979	11.062837	5.889258
H	8.842730	10.780168	4.641860

2c:F$^-$ (rtxF_F_03_a_DCM)

F	-6.227255	0.198945	3.535645
F	-7.677297	-1.375522	3.315560
F	-7.251652	-0.065669	1.663612

F	-5.716588	-5.718133	2.828741
F	-3.658840	-5.900747	2.227150
F	-5.208752	-5.792834	0.740766
O	1.609670	2.043751	-4.607672
O	2.111127	1.601259	-1.323939
O	-2.748238	0.811281	0.360654
N	3.436419	0.791939	-5.086160
N	0.760676	0.346731	-2.611175
H	-0.158120	-0.048287	-2.734527
N	-0.744755	-0.015176	-0.293540
H	-0.140072	-0.819031	-0.227613
N	-2.242932	-1.416936	0.654608
H	-1.497085	-2.193475	0.403973
C	5.244450	-0.368376	-3.768371
C	6.225634	-1.355610	-3.742033
H	6.247381	-2.110297	-4.520833
C	-4.816515	-5.305722	1.927317
C	-6.691807	-0.725368	2.686266
C	7.170485	-1.395734	-2.729678
H	7.921140	-2.175165	-2.724035
C	7.149304	-0.439340	-1.724452
H	7.883888	-0.465008	-0.930918
C	6.172257	0.542276	-1.740775
H	6.143352	1.290824	-0.960145
C	5.221515	0.577679	-2.752092
H	4.468230	1.353564	-2.732960
C	4.217976	-0.407673	-4.881604
H	4.731248	-0.626212	-5.822408
H	3.557029	-1.264356	-4.723338
C	3.986123	1.814673	-5.961002
H	3.162482	2.430591	-6.317904
H	4.420410	1.316776	-6.831932
C	5.026964	2.669742	-5.285314
C	6.381091	2.436340	-5.486699
H	6.694021	1.651474	-6.165577
C	7.338033	3.180625	-4.812573
H	8.389086	2.978361	-4.972095
C	6.945323	4.171802	-3.927242
H	7.688631	4.751711	-3.395441
C	5.593660	4.417971	-3.725688
H	5.280196	5.191971	-3.037139
C	4.639859	3.673744	-4.401051
H	3.586255	3.858398	-4.231828
C	2.262409	1.038216	-4.435711
C	1.804010	-0.065467	-3.491927
H	1.441820	-0.909882	-4.078012
H	2.651178	-0.436904	-2.912823
C	1.015423	1.103849	-1.536090
C	-0.156371	1.269829	-0.582931
H	0.204869	1.779118	0.310816
H	-0.902705	1.920540	-1.044346
C	-1.972877	-0.149582	0.262234
C	-3.423465	-1.866496	1.204108
C	-4.474301	-1.061957	1.654370
H	-4.401501	0.010064	1.568794
C	-5.602923	-1.642700	2.216527
C	-5.734810	-3.014980	2.333272
H	-6.620611	-3.453219	2.767524
C	-4.691113	-3.814098	1.883179
C	-3.553069	-3.257370	1.333189
H	-2.729062	-3.876948	0.998518
F	-2.965636	3.693104	-0.446134

F	-6.135475	3.579347	2.990461
F	-8.036747	2.866655	1.223793
F	-7.363980	2.381655	-1.335896
F	1.836727	-1.597510	-0.528084
F	3.938550	-4.985373	-2.990857
F	6.179824	-4.602055	-1.595362
F	6.272241	-2.752850	0.339532
O	-3.508620	4.967637	3.018876
O	-5.325462	3.257763	-3.311518
O	1.846047	-3.383360	-3.947407
O	4.460029	-1.480901	2.183269
N	-2.502659	3.004572	2.462582
H	-2.548474	2.196818	1.850643
N	-3.895935	1.701718	-2.493071
H	-3.476183	1.329095	-1.650741
N	0.428098	-3.654706	-2.194820
H	0.175641	-3.494620	-1.176290
N	3.754668	0.131776	0.754806
H	3.436706	0.358463	-0.176561
C	-3.443419	3.960252	2.349792
C	-4.489951	3.618441	1.318983
C	-4.203486	3.437352	-0.022138
C	-5.137835	3.005406	-0.946521
C	-6.434331	2.819067	-0.499088
C	-6.782540	3.041440	0.820346
C	-5.802695	3.426847	1.717272
C	-4.794176	2.696299	-2.380302
C	-3.486725	1.174831	-3.773681
H	-2.792393	1.858259	-4.272869
H	-4.367518	1.109681	-4.418201
C	-2.841053	-0.178039	-3.627284

C	-1.814935	-0.556658	-4.490039
H	-1.489522	0.124510	-5.268067
C	-1.175665	-1.779837	-4.344236
H	-0.377891	-2.057543	-5.022507
C	-1.519520	-2.642921	-3.307666
C	-2.576514	-2.285469	-2.481467
H	-2.881332	-2.950994	-1.684398
C	-3.242562	-1.082200	-2.649352
H	-4.066134	-0.845412	-1.986572
C	-0.728774	-3.896445	-3.035971
H	-1.358862	-4.618046	-2.514034
H	-0.406936	-4.358436	-3.968546
C	1.619016	-3.438159	-2.743704
C	2.799394	-3.294607	-1.808728
C	2.899759	-2.356058	-0.795766
C	4.044538	-2.118538	-0.052651
C	5.143096	-2.912924	-0.330383
C	5.101210	-3.868956	-1.327189
C	3.939885	-4.048840	-2.054313
C	4.101128	-1.126721	1.080916
C	3.744090	1.215537	1.706339
H	4.281761	2.061737	1.272356
H	4.308246	0.894548	2.582020
C	2.364123	1.662361	2.120554
C	1.443955	0.762132	2.647880
H	1.702958	-0.285929	2.736161
C	0.202014	1.199663	3.083199
H	-0.496178	0.483025	3.500586
C	-0.155553	2.542468	3.001119
C	0.755749	3.434485	2.447805
H	0.489743	4.480614	2.353887

C	1.997417	2.998384	2.012844	H	-1.815781	2.391571	4.337397
H	2.690494	3.709035	1.579180	H	-1.419047	4.035752	3.880615
C	-1.493946	3.018393	3.498874	F	-0.583728	-3.138721	0.064502

11. ^{1}H, ^{13}C and ^{19}F NMR Spectra of synthesized compounds

1a (^{1}H NMR, 400 MHz, $CDCl_3$, 298 K)

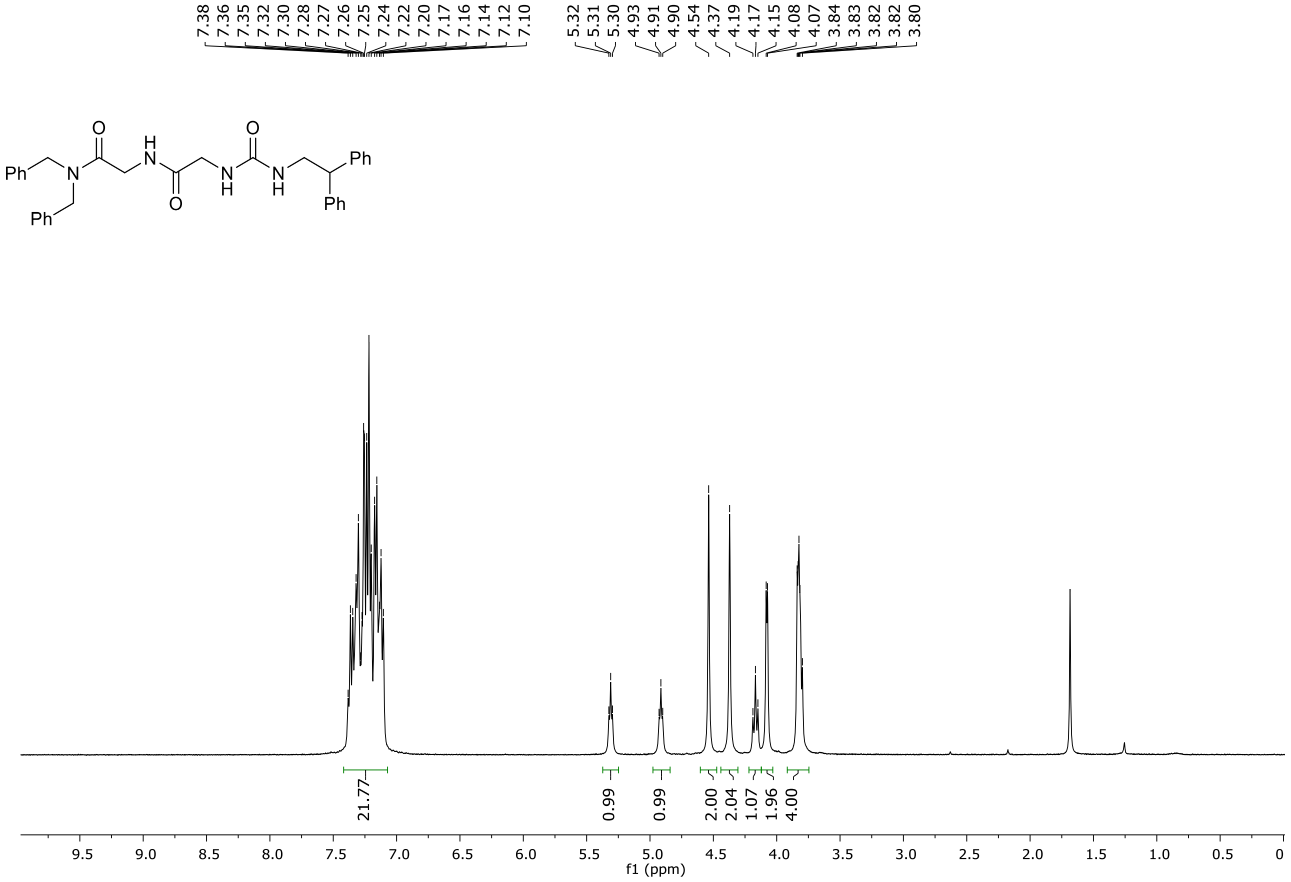

1a (COSY ^{1}H-^{1}H NMR, 400 MHz, $CDCl_3$, 298 K)

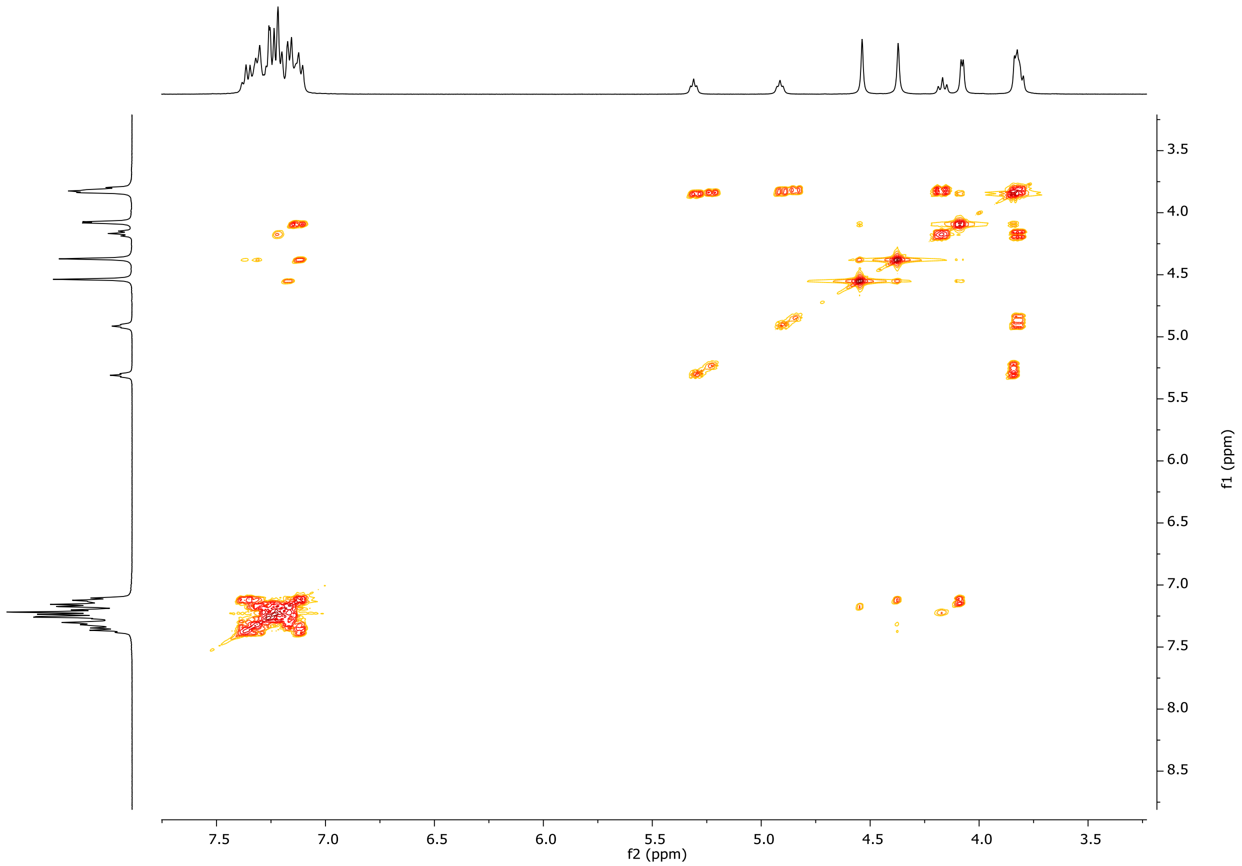

1a (^{1}H NMR, 400 MHz, DMSO-d$_6$, 298 K)

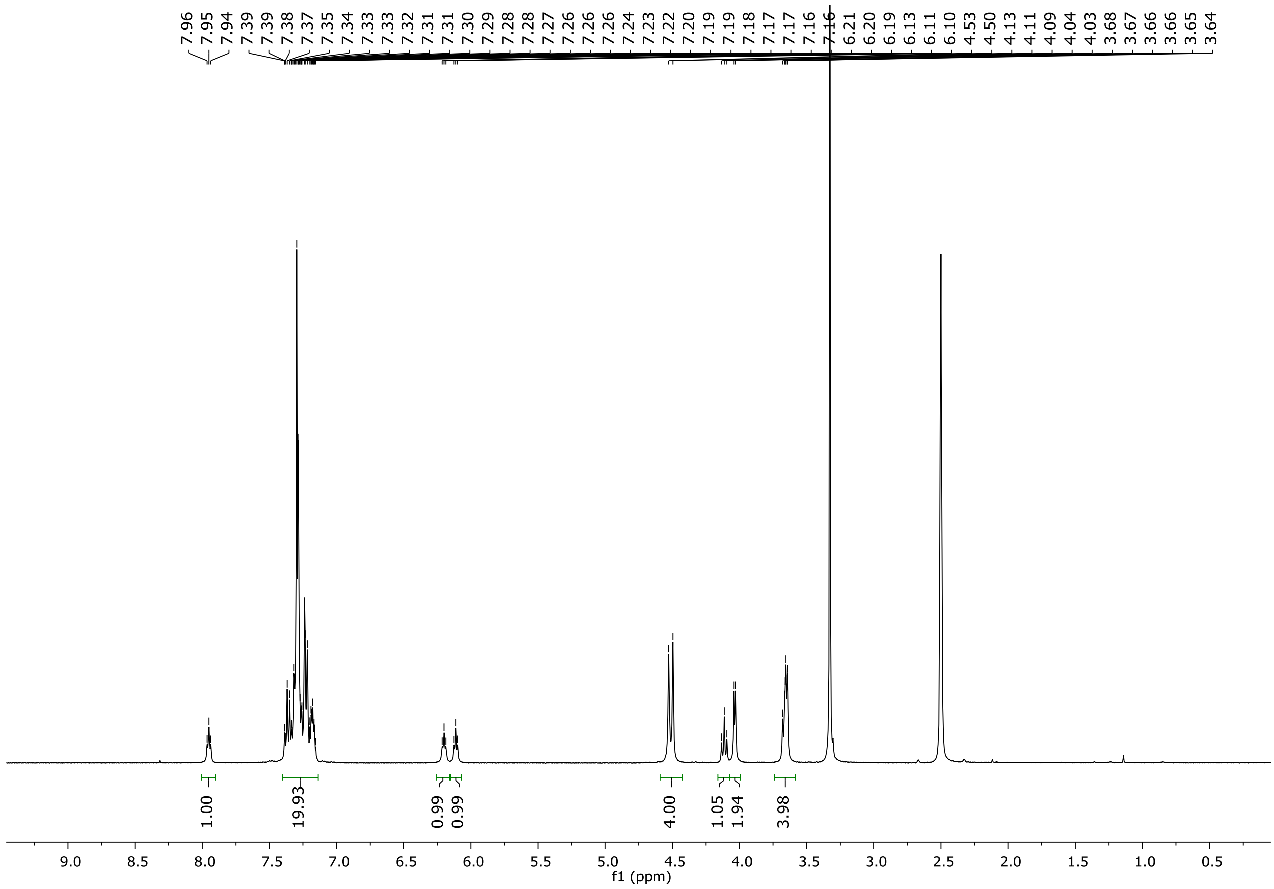

1a (COSY ^{1}H-^{1}H NMR, 400 MHz, DMSO-d$_6$, 298 K)

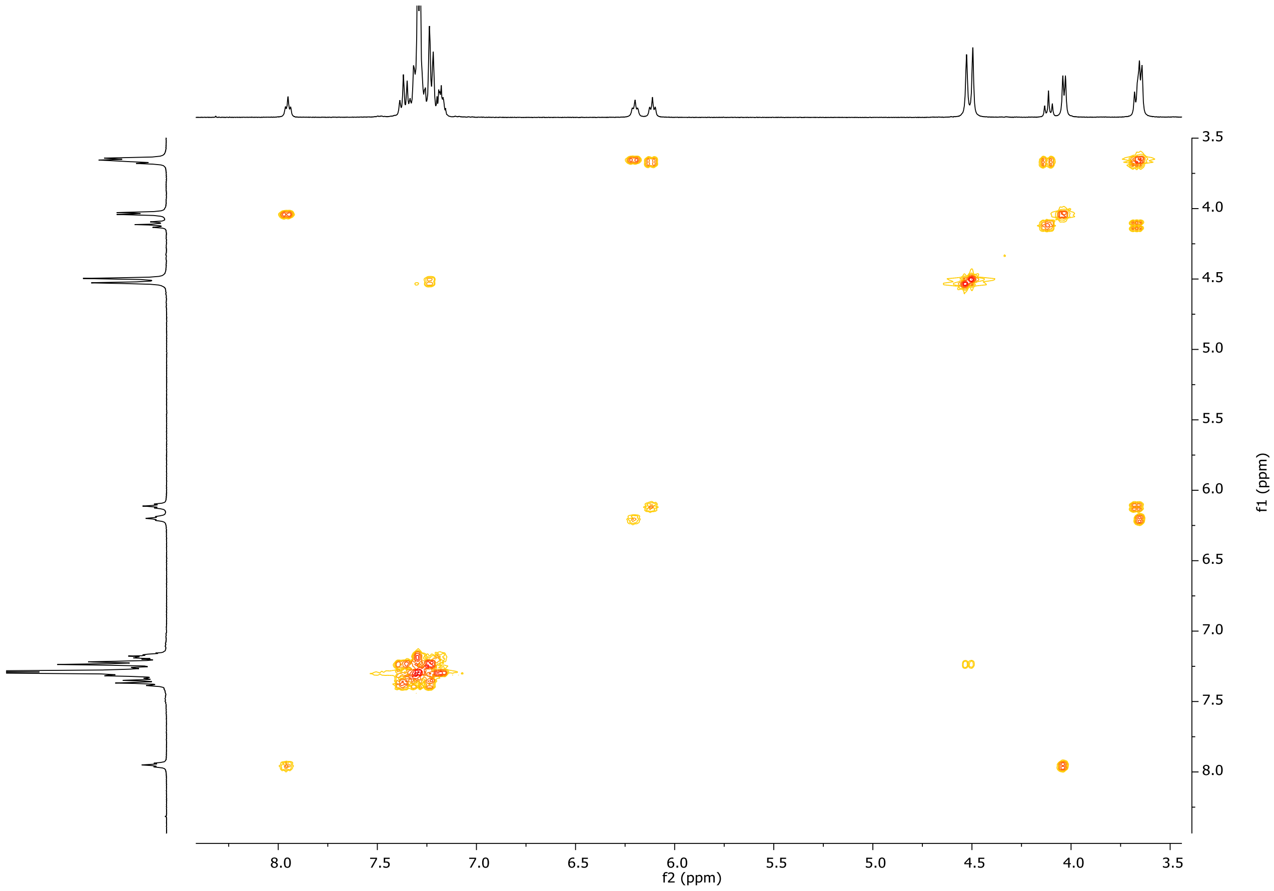

1a (^{13}C NMR, 100 MHz, $CDCl_3$, 298 K)

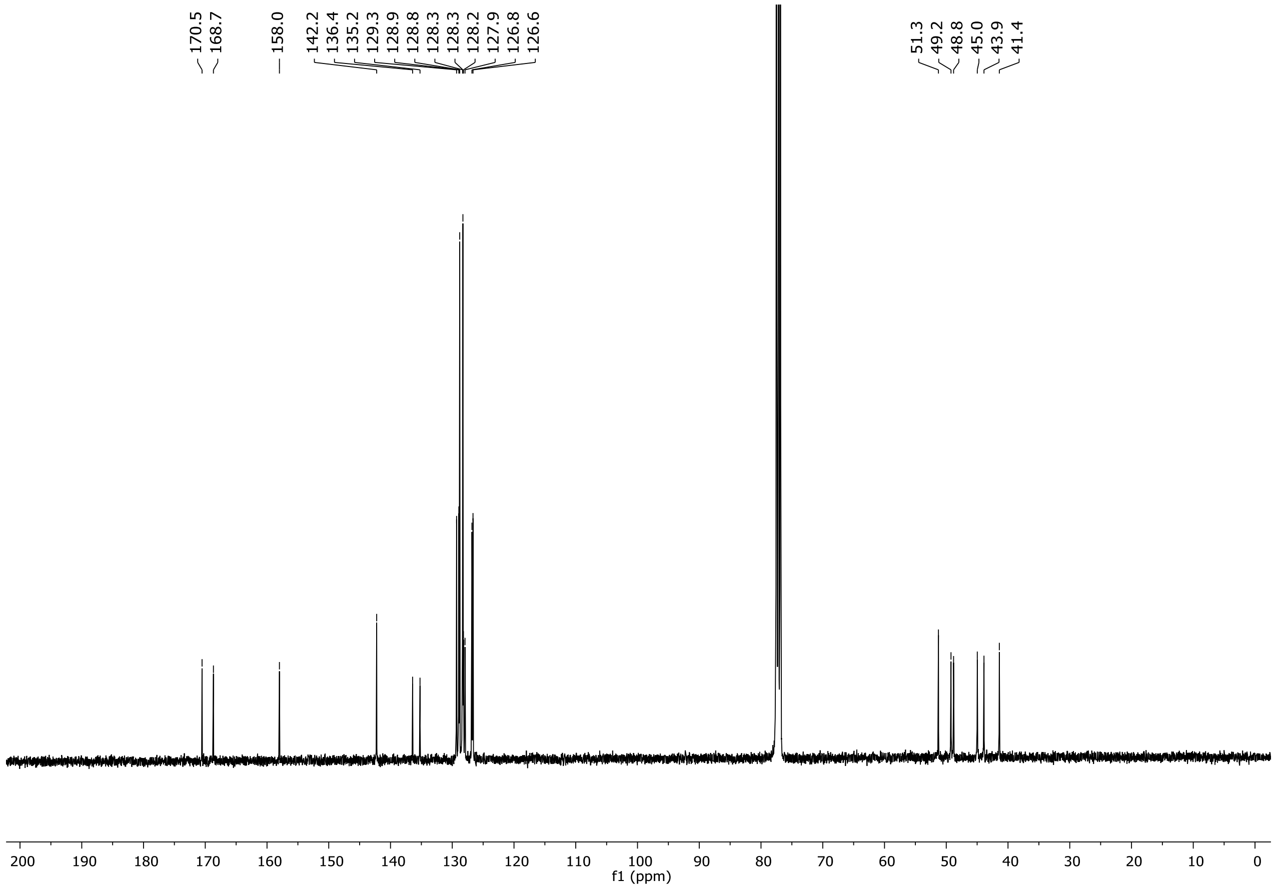

1a (DEPT ^{13}C NMR, 100 MHz, $CDCl_3$, 298 K)

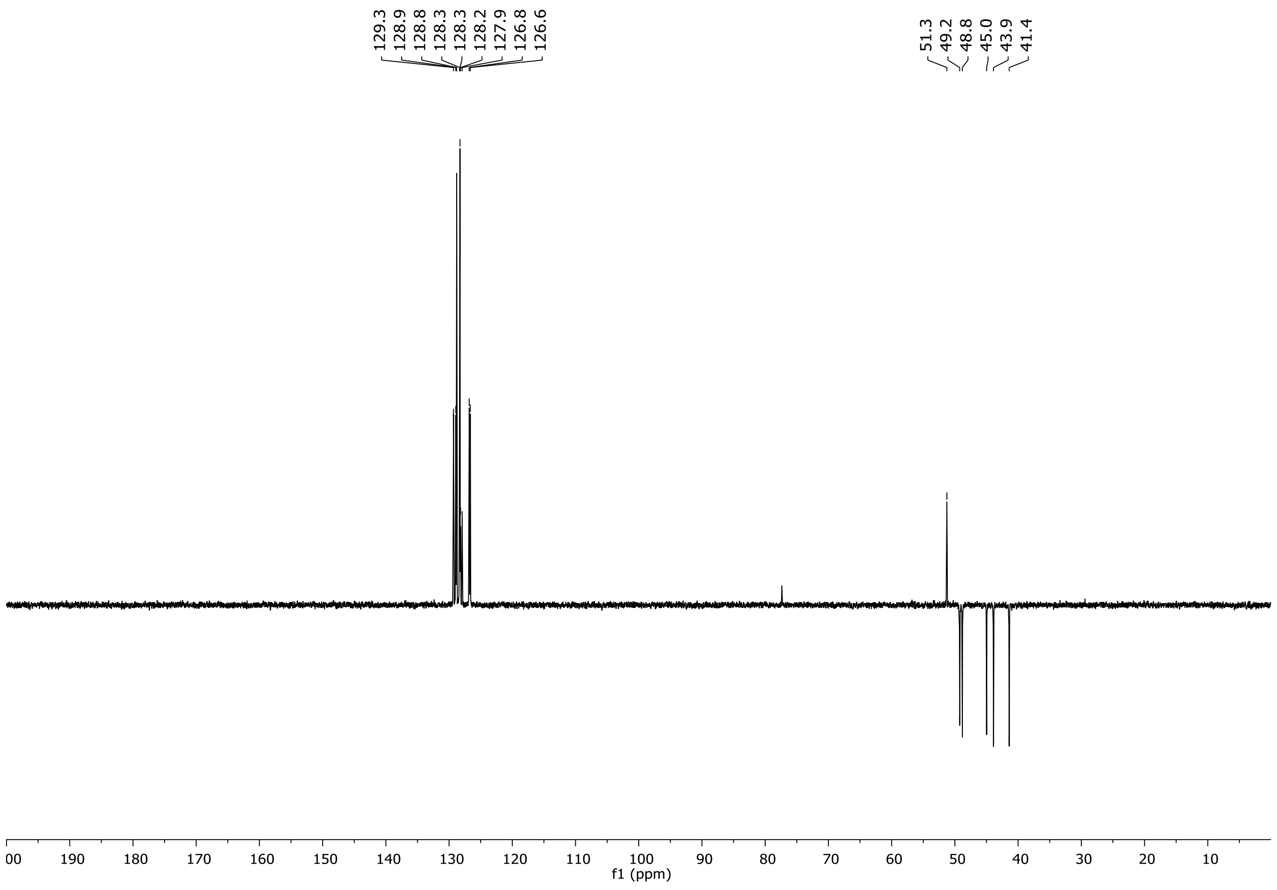

1b (^{1}H NMR, 400 MHz, $CDCl_3$, 298 K)

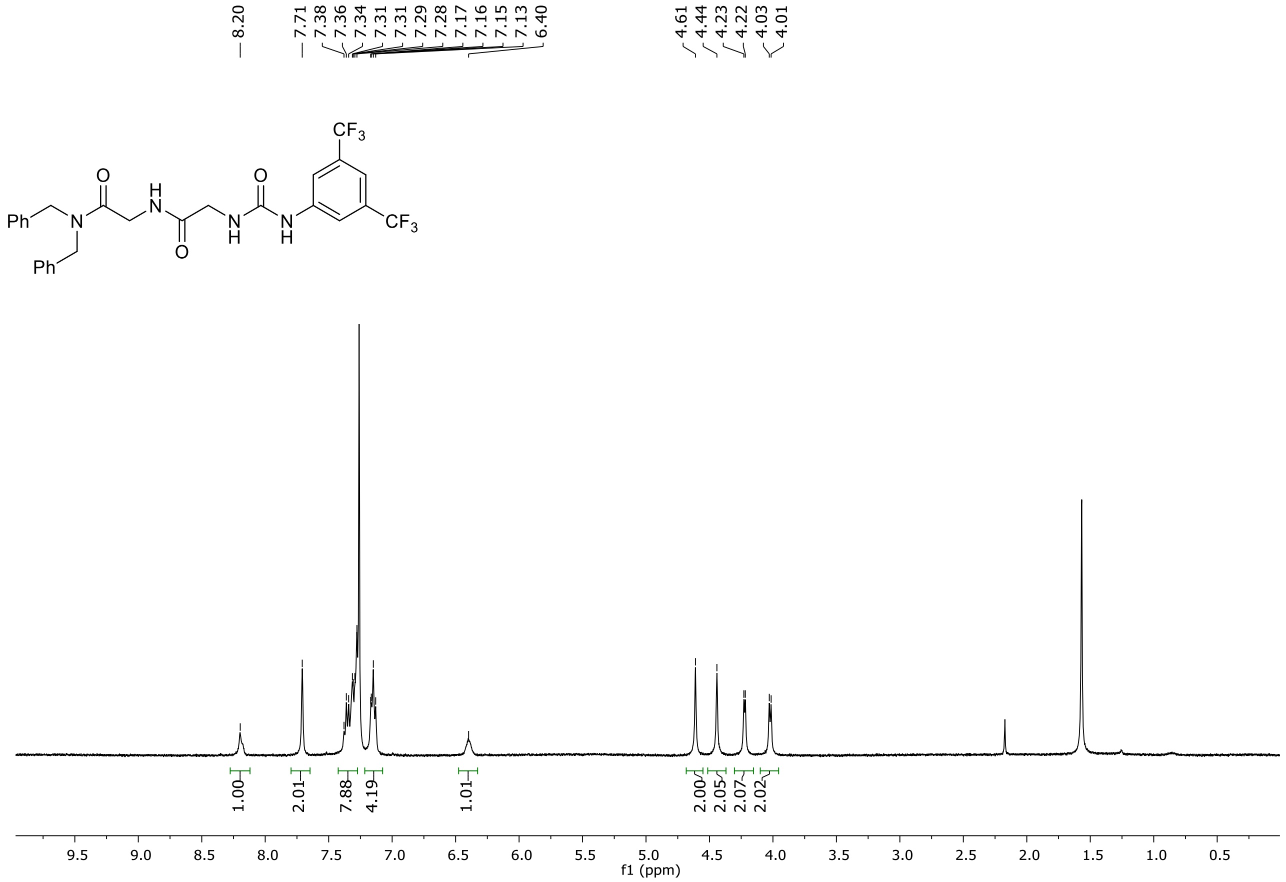

1b (COSY ^{1}H-^{1}H NMR, 400 MHz, $CDCl_3$, 298 K)

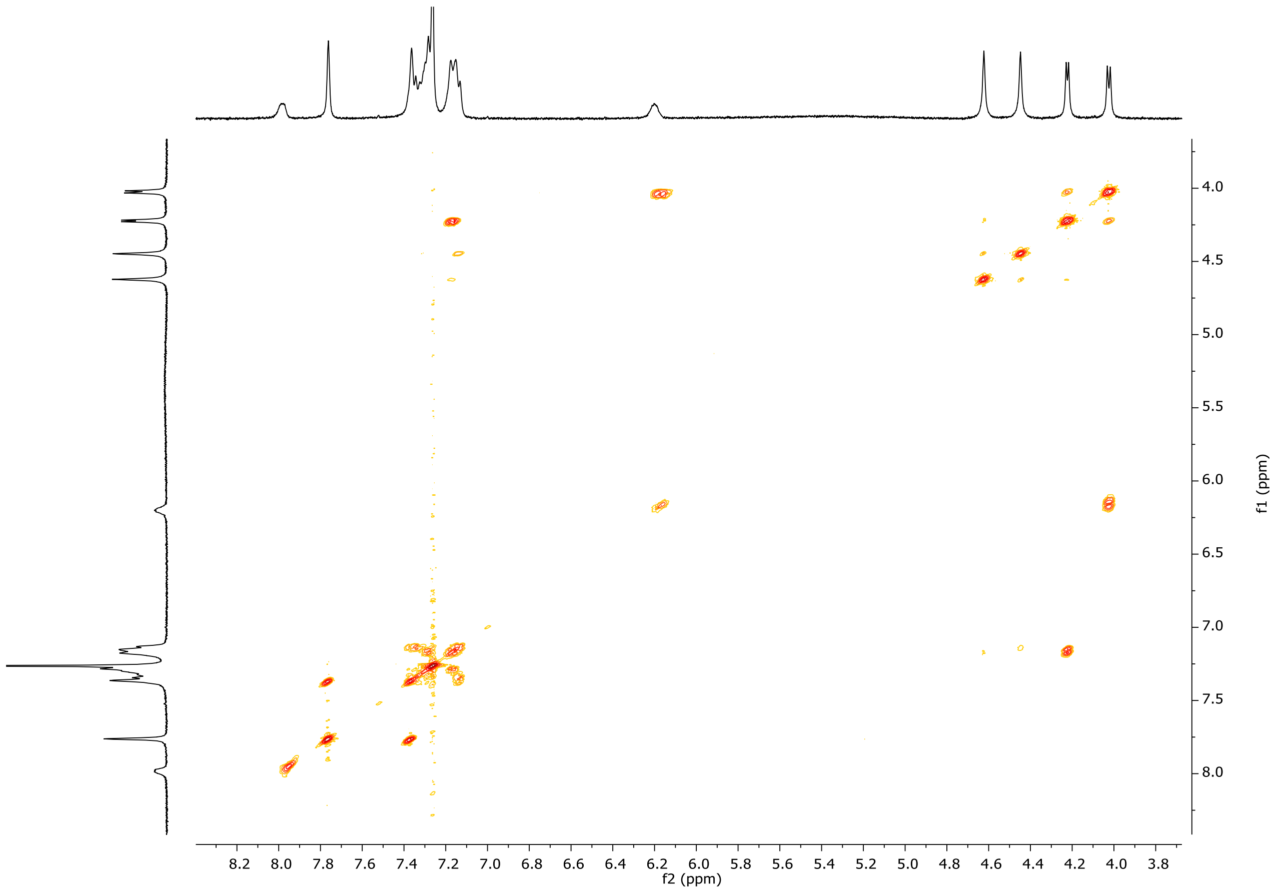

1b (^{1}H NMR, 400 MHz, DMSO-d_6, 298 K)

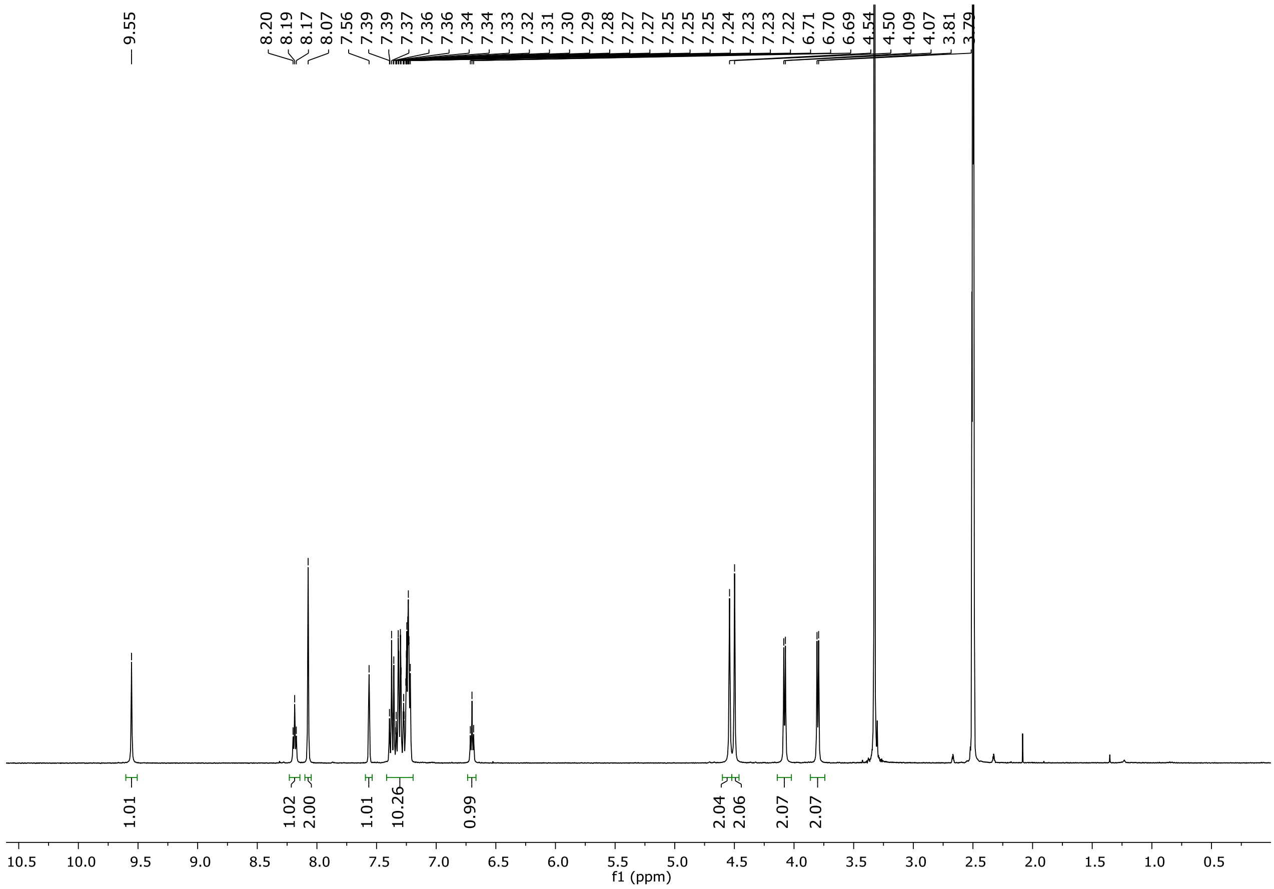

1b (COSY ^{1}H-^{1}H NMR, 400 MHz, DMSO-d_6, 298 K)

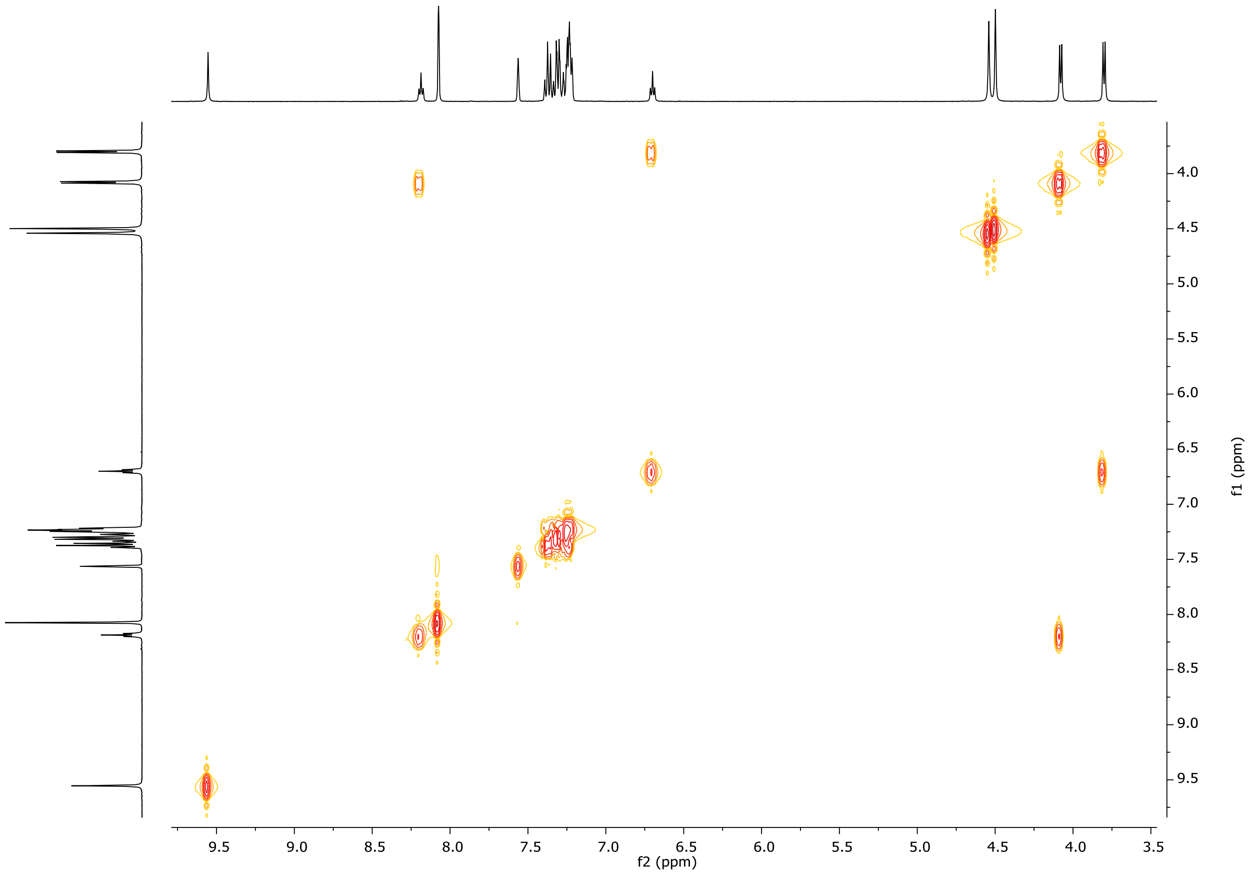

1b (^{13}C NMR, 100 MHz, DMSO-d_6, 298 K)

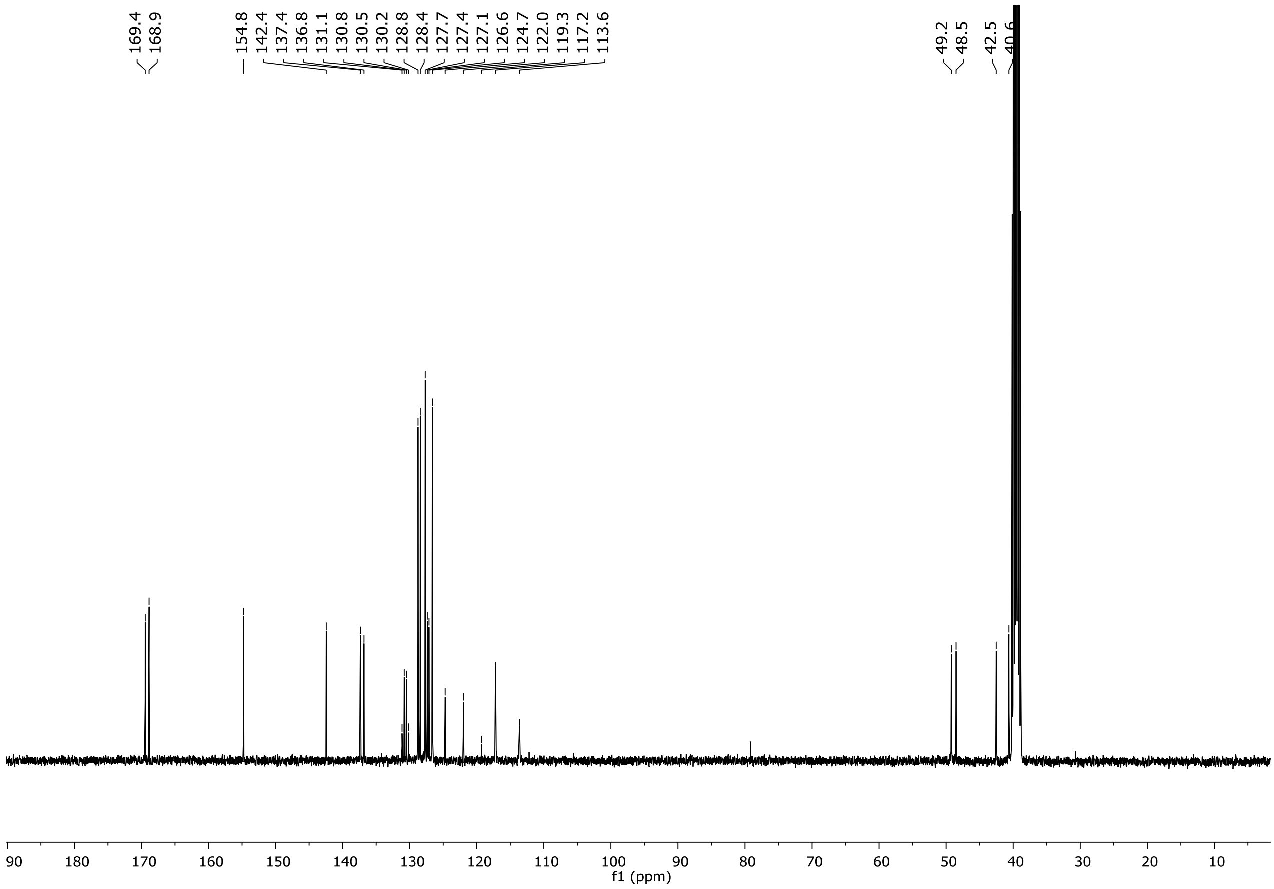

1b (DEPT ^{13}C NMR, 100 MHz, DMSO-d_6, 298 K)

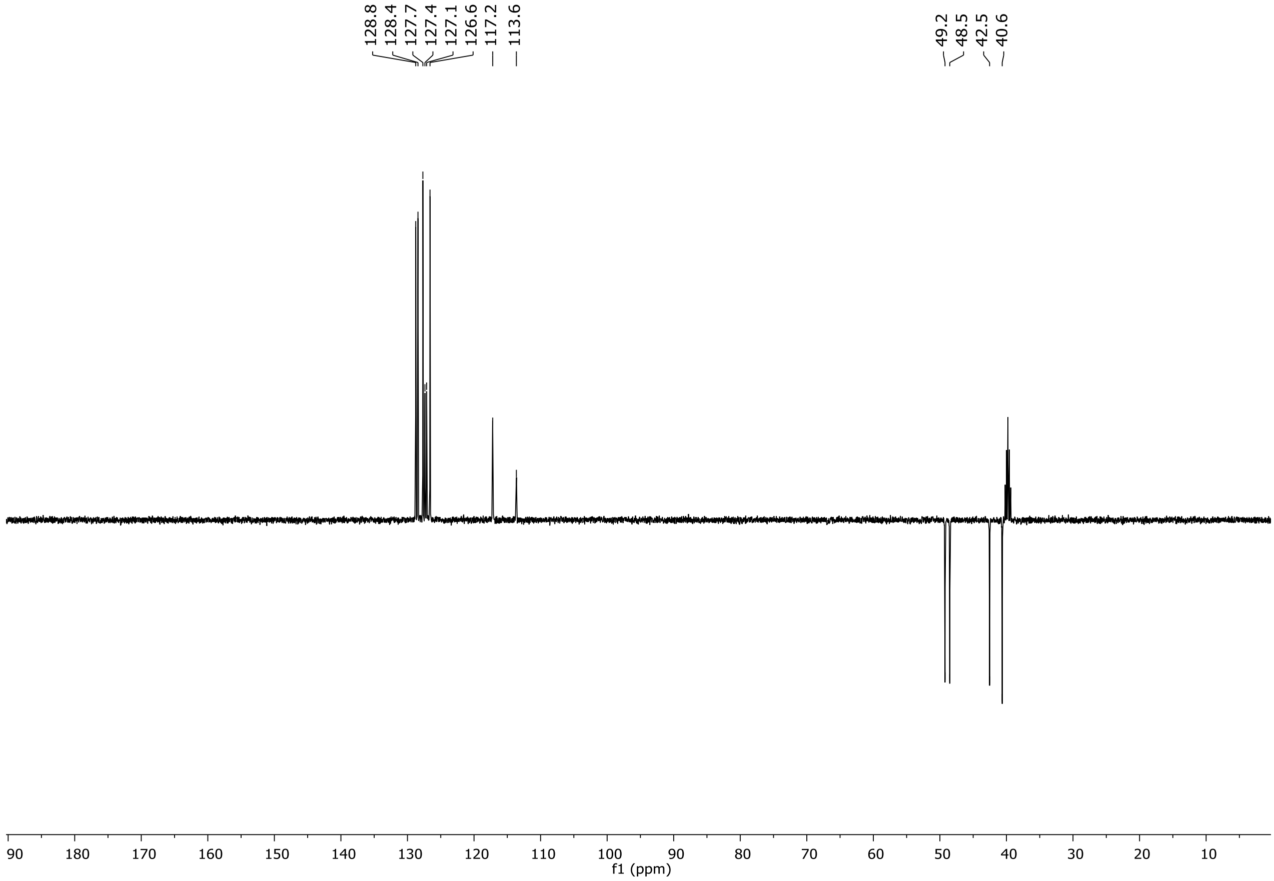

1b (^{19}F NMR, 376 MHz, DMSO-d_6, 298 K)

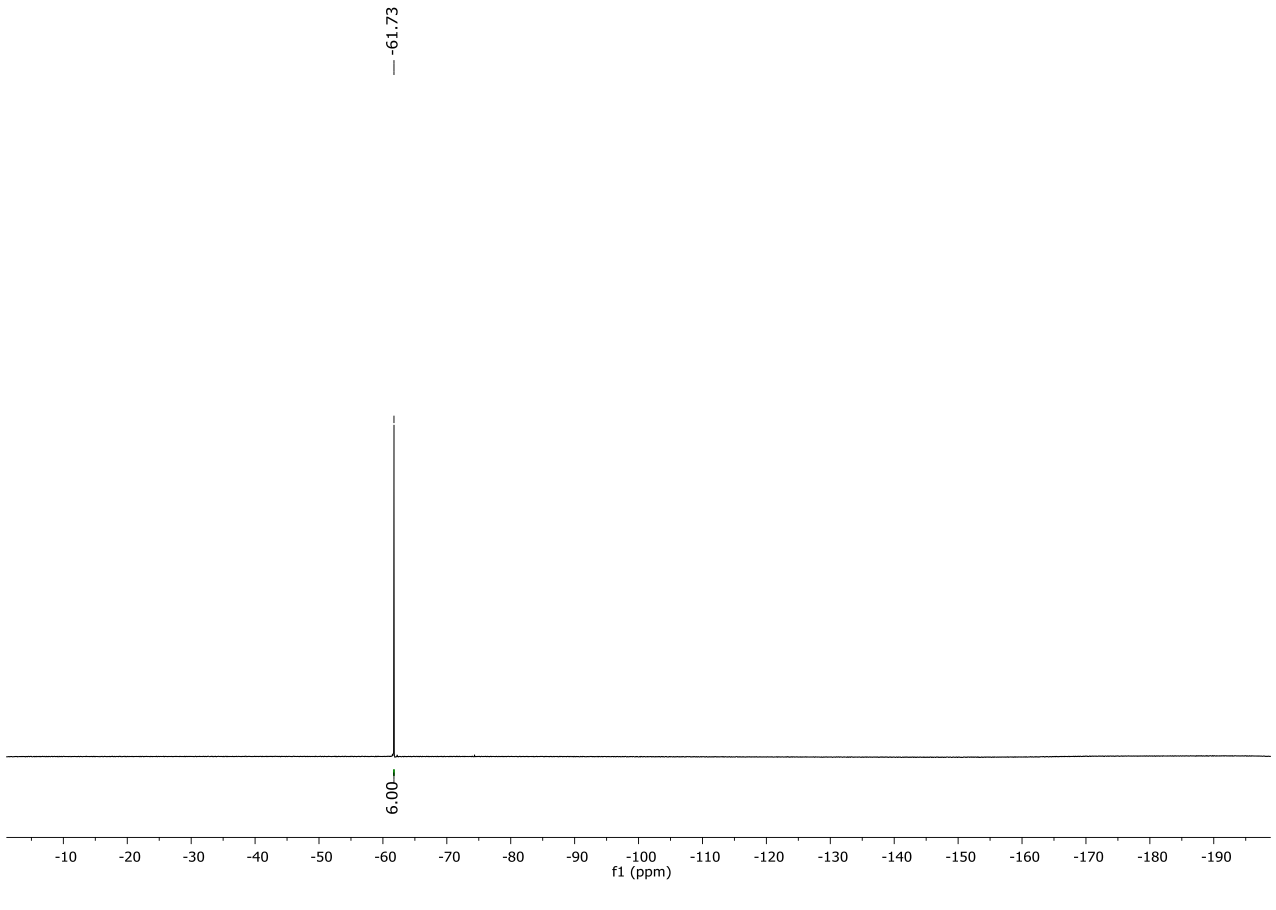

2a (^{1}H NMR, 400 MHz, $CDCl_3$, 298 K)

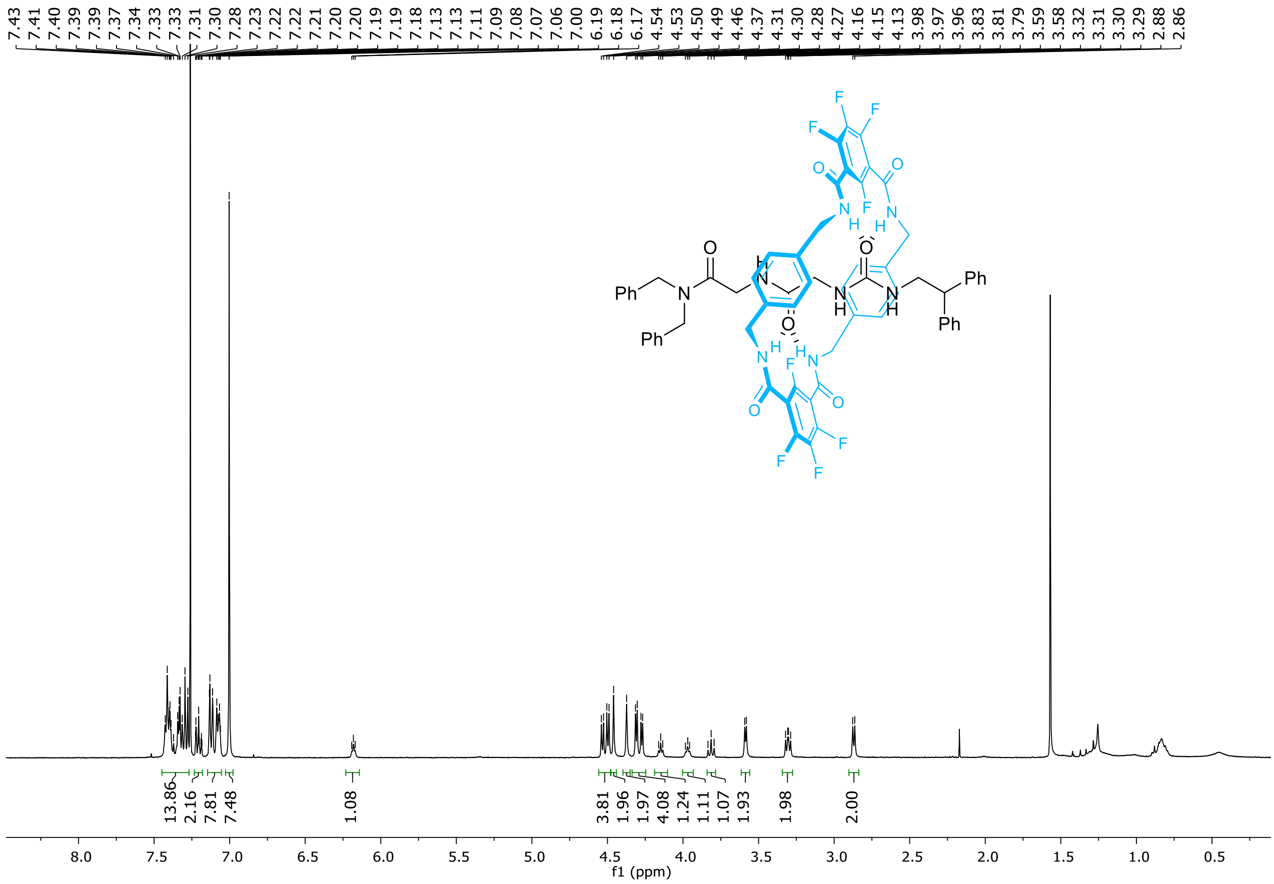

2a (COSY ^{1}H-^{1}H NMR, 400 MHz, $CDCl_3$, 298 K)

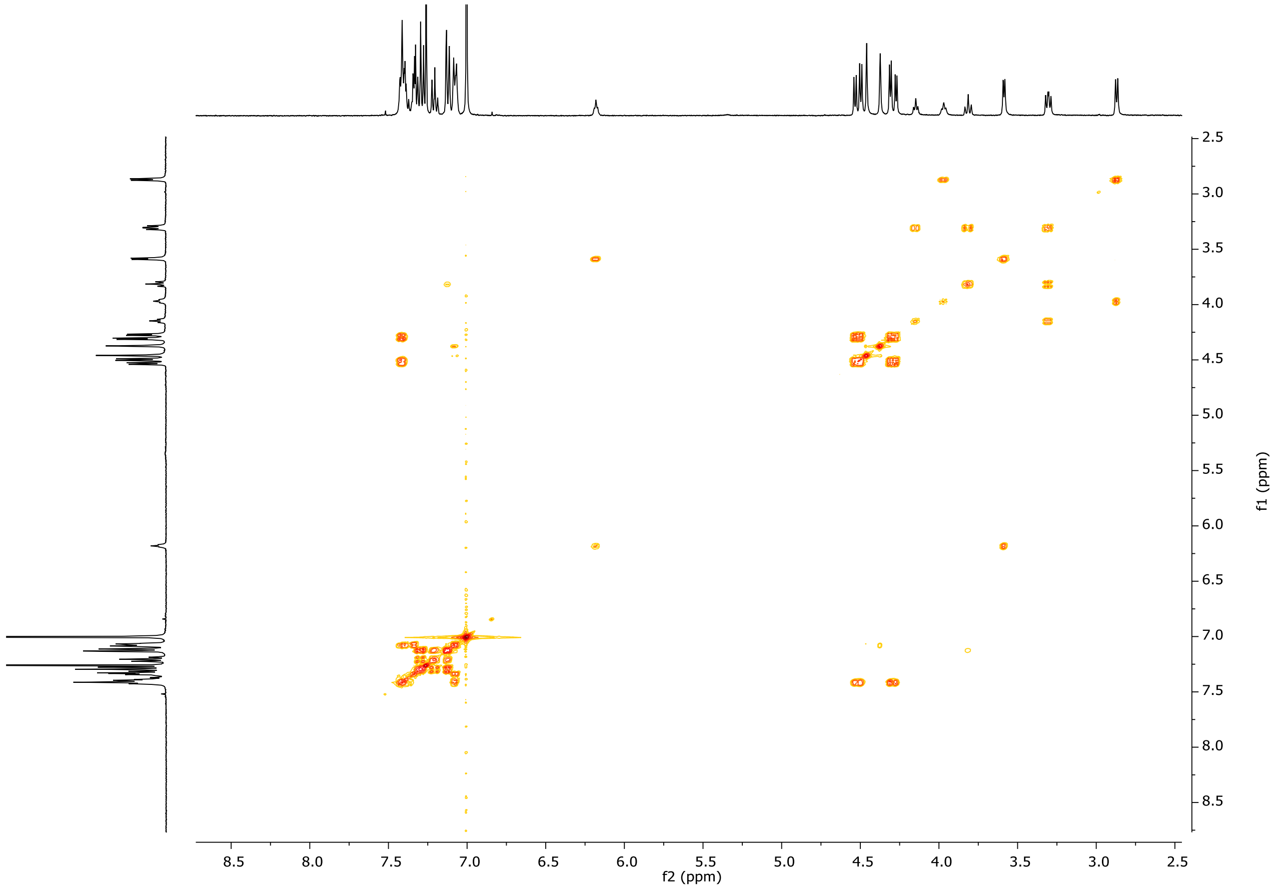

2a (NOESY ^1H-^1H NMR, 400 MHz, $CDCl_3$, 298 K)

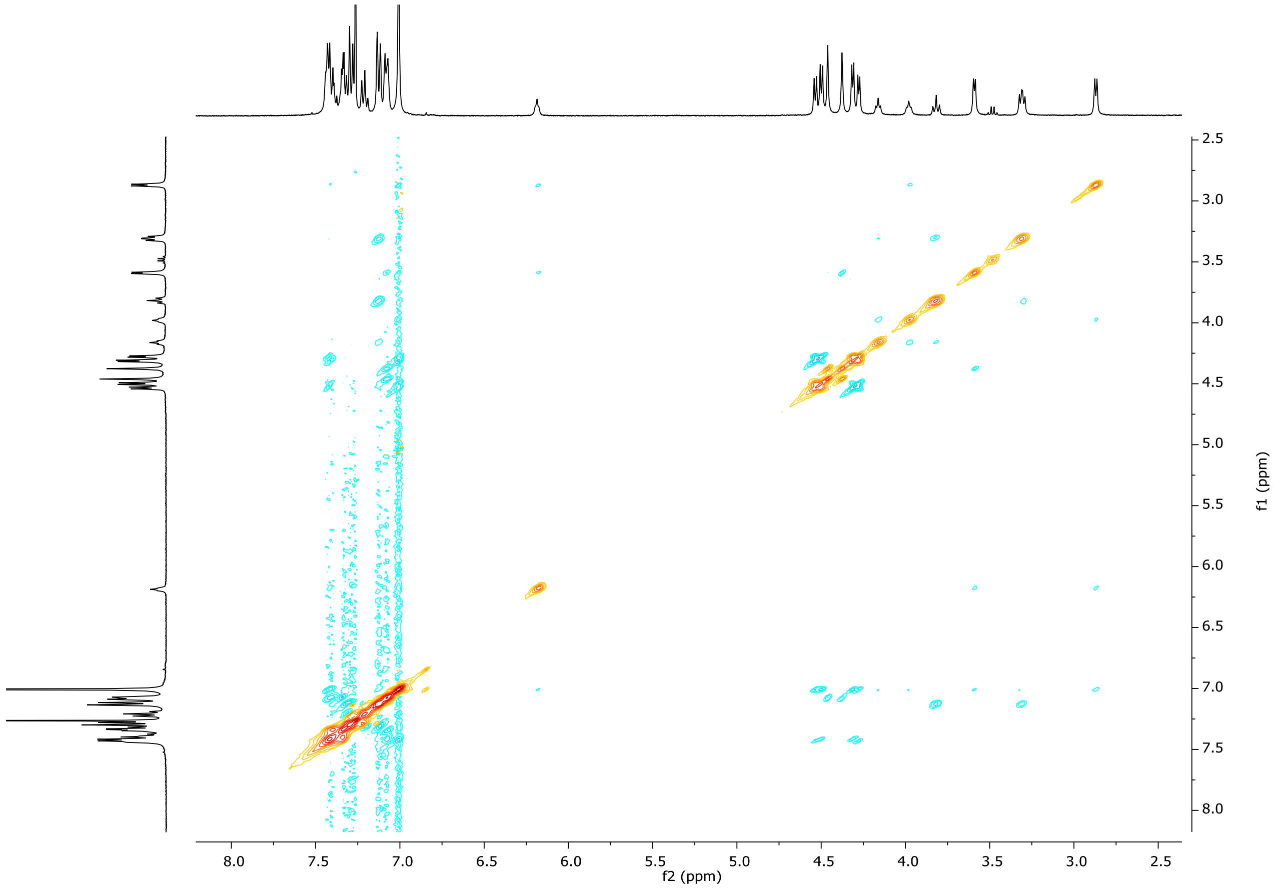

2a (^1H NMR, 400 MHz, DMSO-d_6, 298 K)

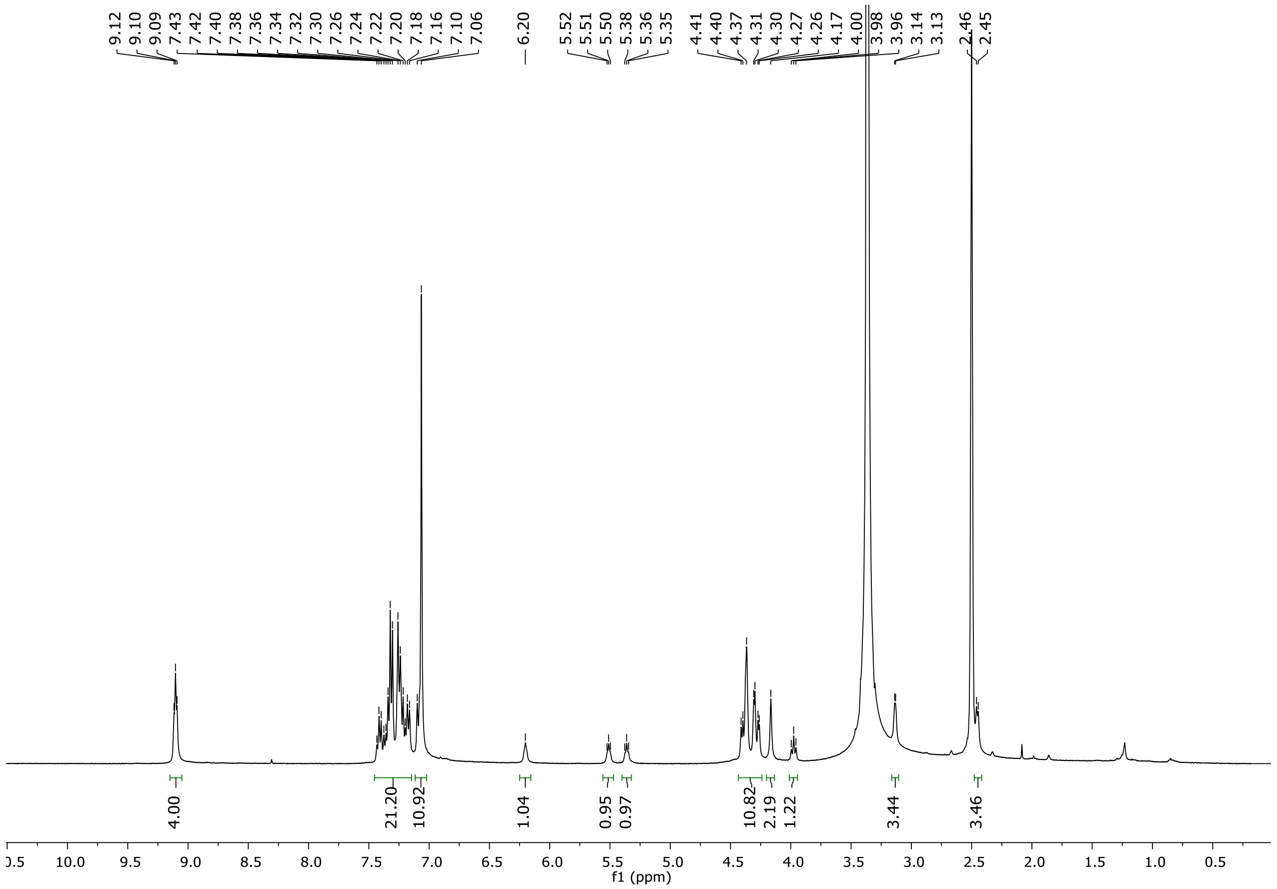

2a (COSY ^{1}H-^{1}H NMR, 400 MHz, DMSO-d$_6$, 298 K)

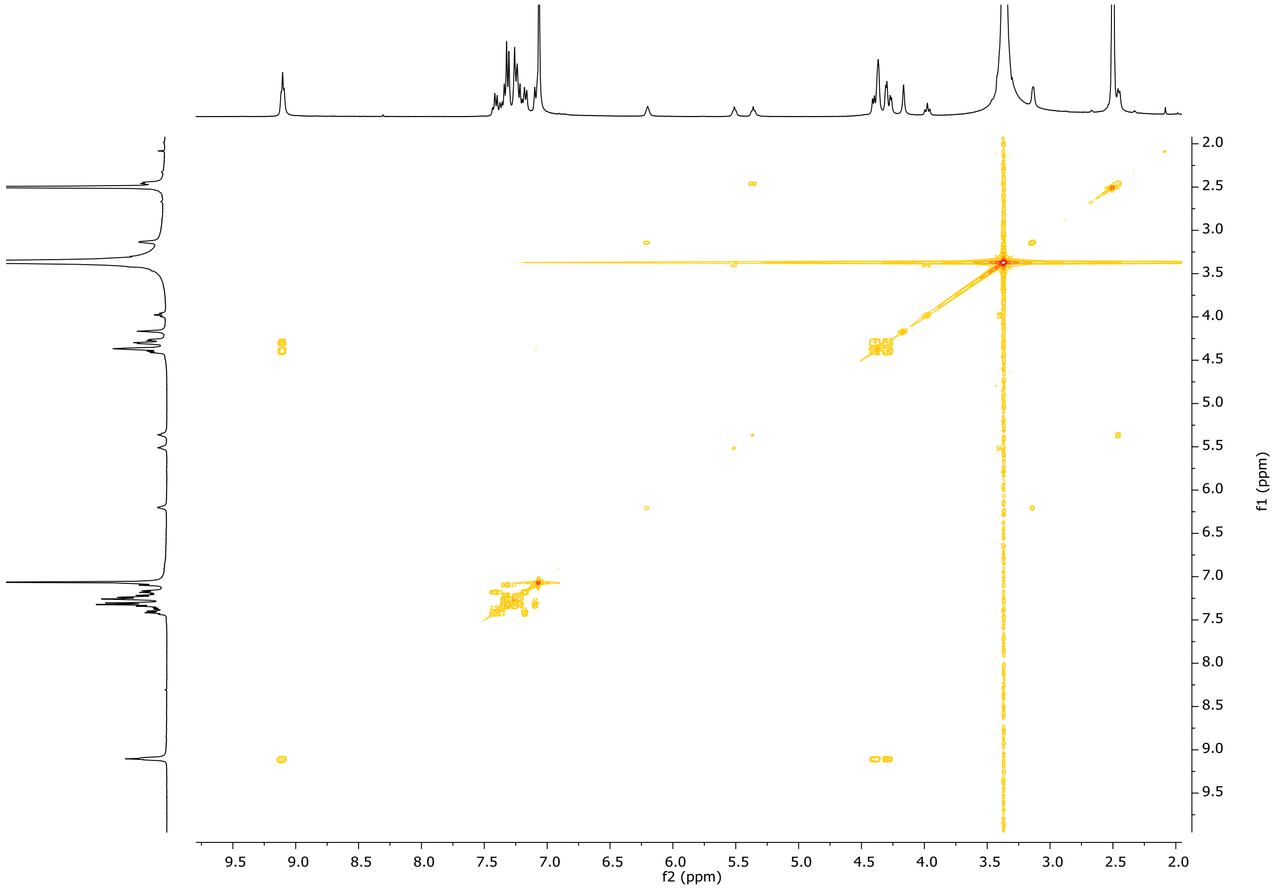

2a (^{13}C NMR, 100 MHz, $CDCl_3$, 298 K)

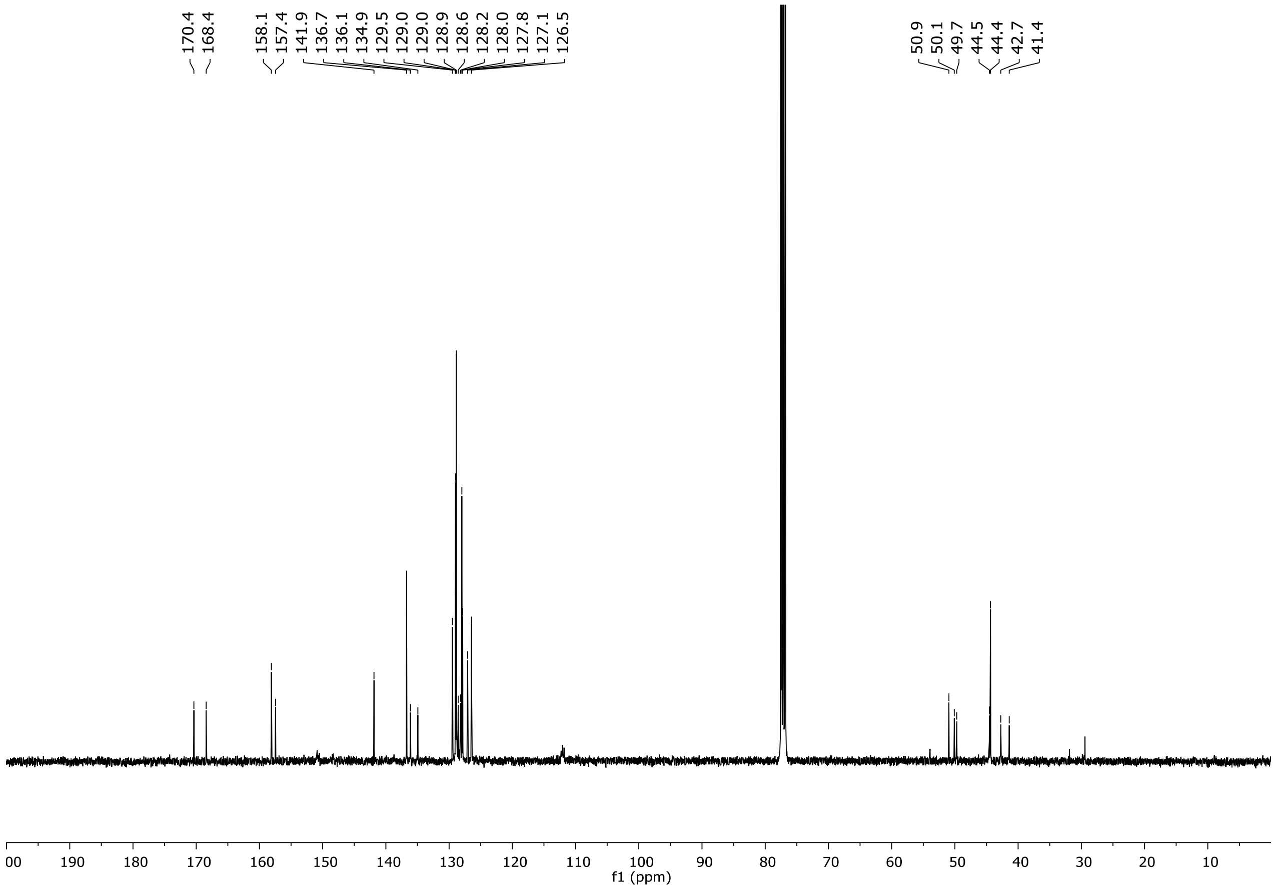

2a (DEPT ^{13}C NMR, 100 MHz, $CDCl_3$, 298 K)

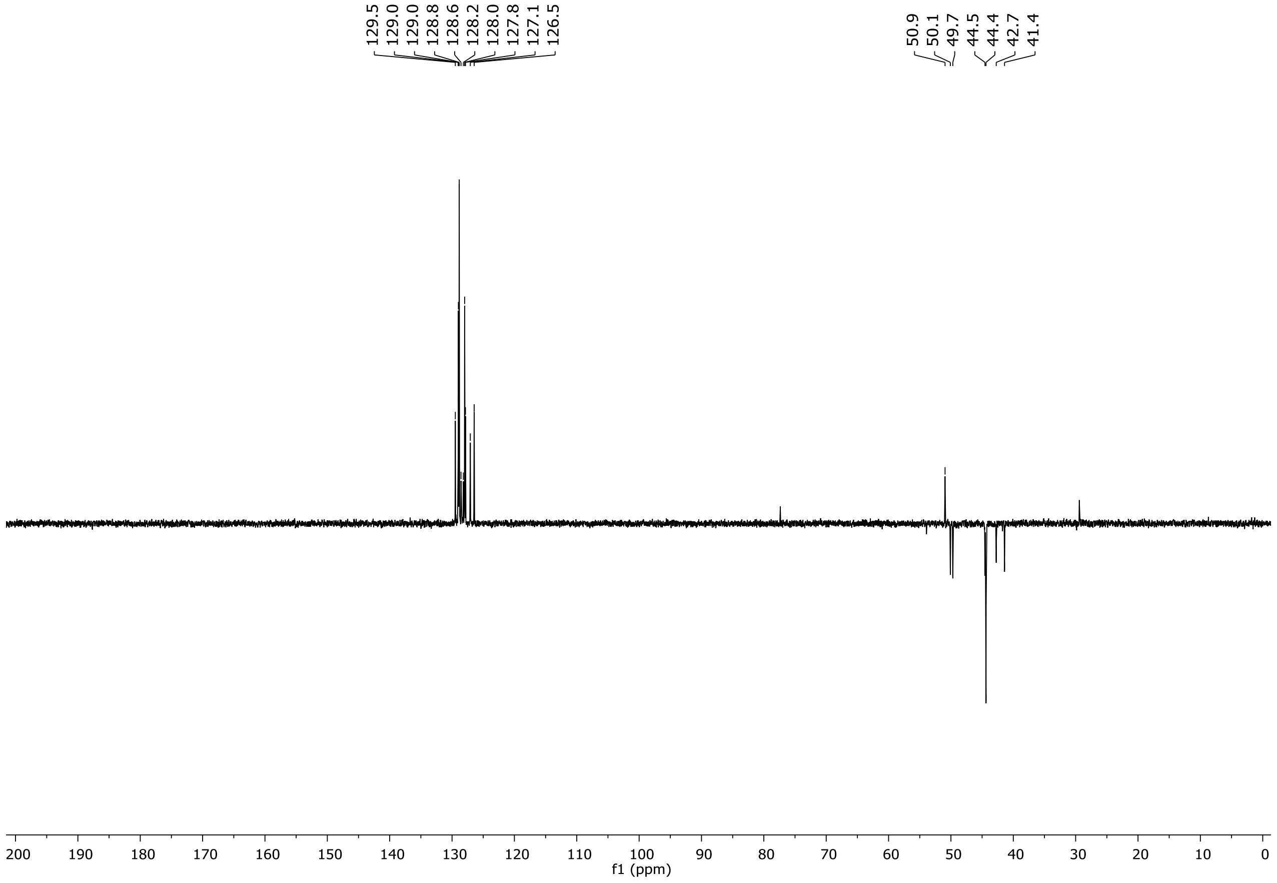

2a (^{19}F NMR, 376 MHz, $CDCl_3$, 298 K)

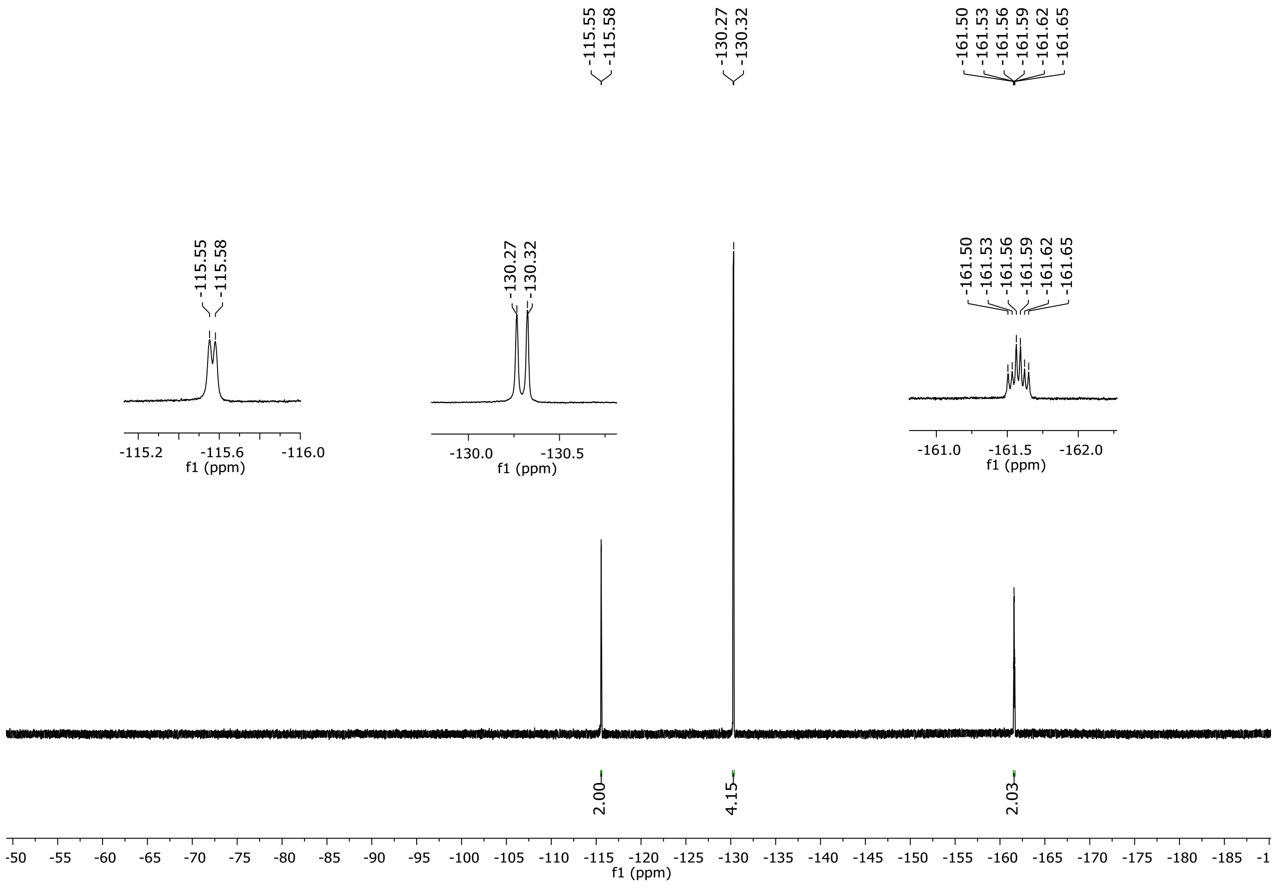

2b (^{1}H NMR, 400 MHz, CDCl$_3$, 323 K)

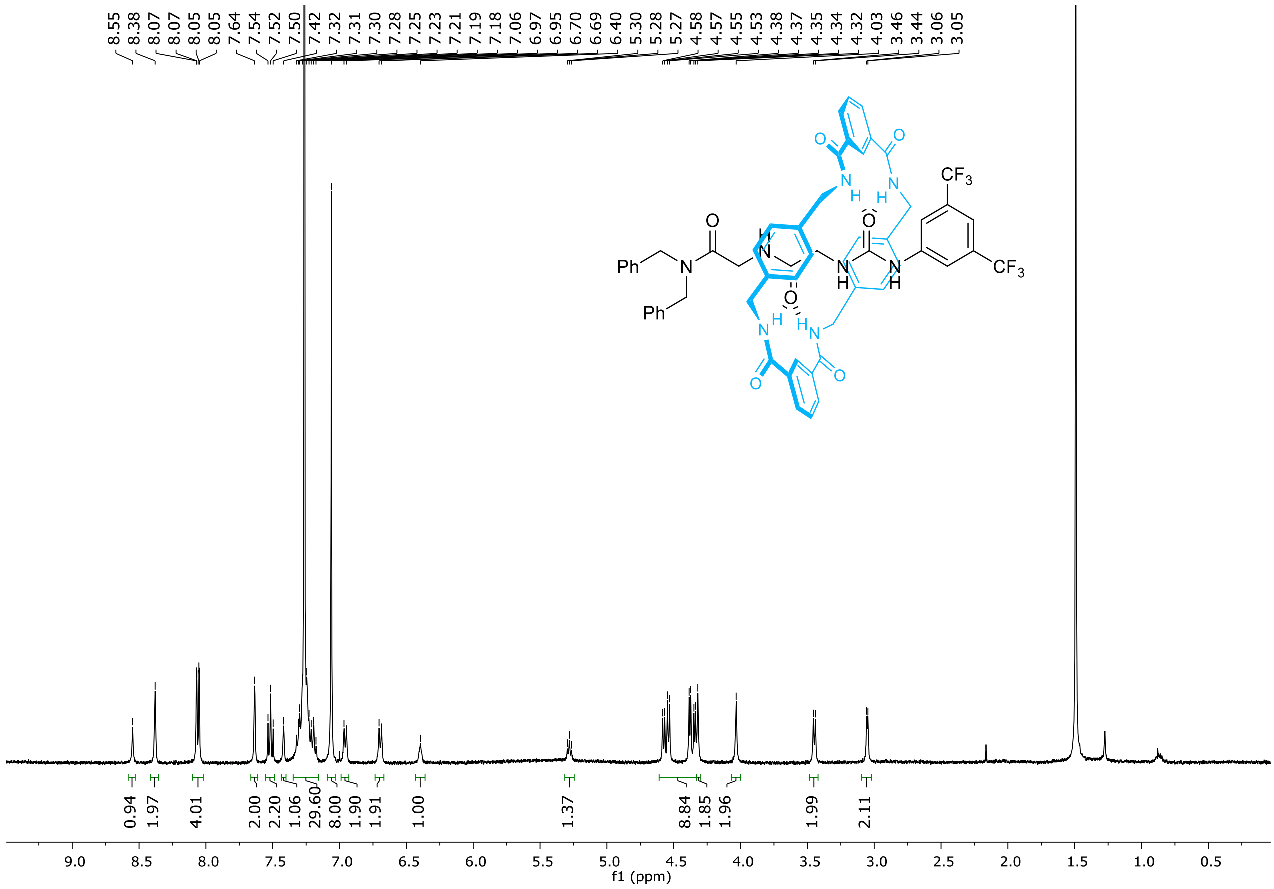

2b (COSY ^{1}H-^{1}H NMR, 400 MHz, CDCl$_3$, 323 K)

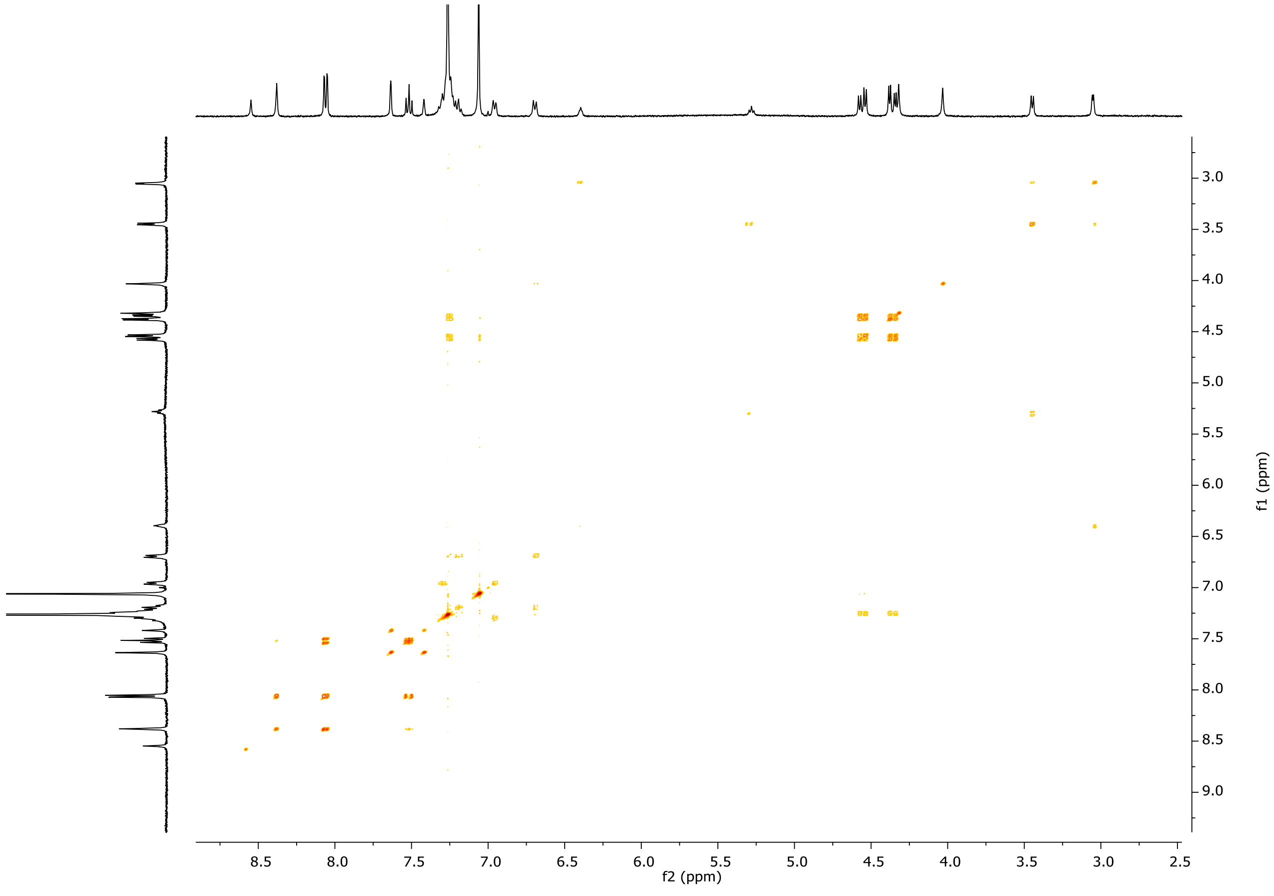

2b (^{1}H NMR, 400 MHz, DMSO-d$_6$, 298 K)

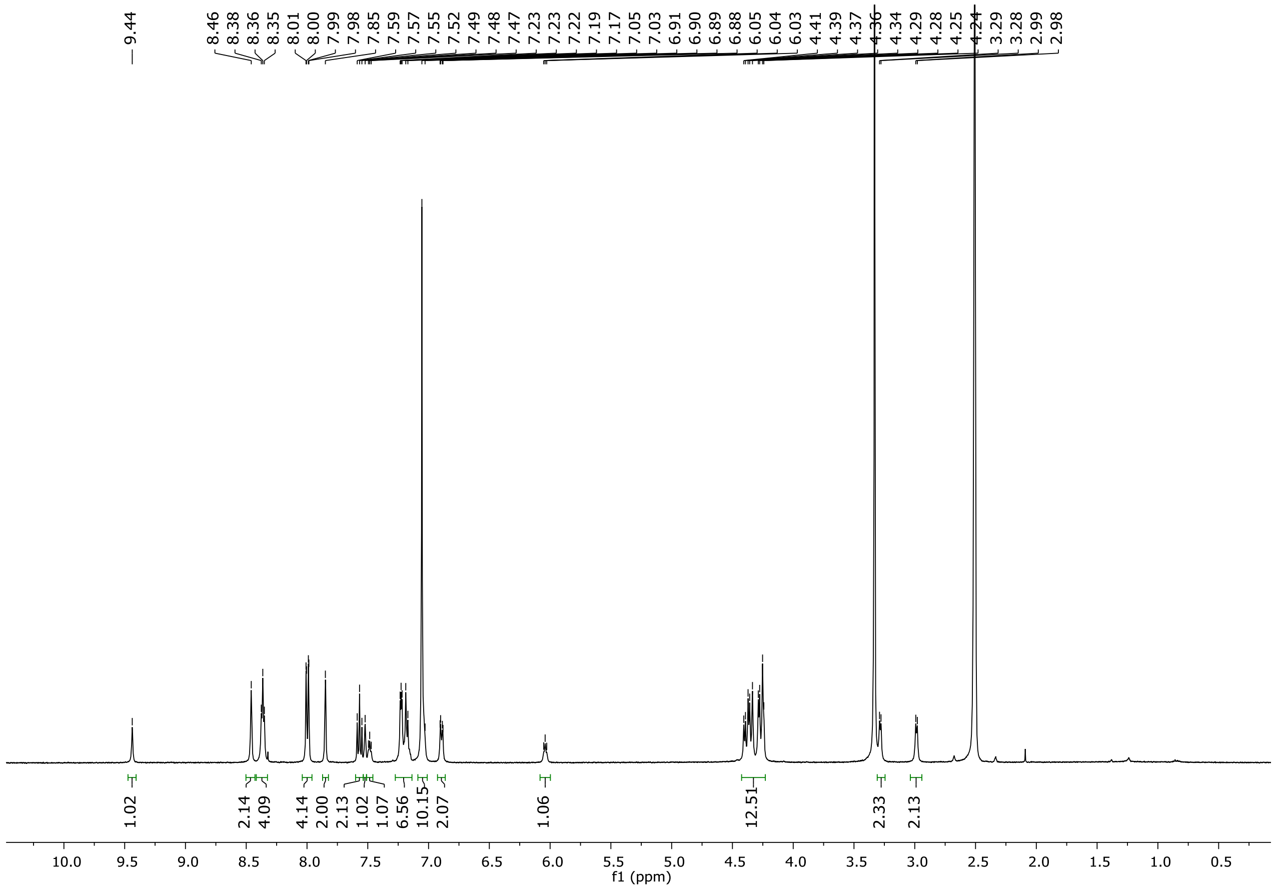

2b (COSY ^{1}H-^{1}H NMR, 400 MHz, DMSO-d$_6$, 298 K)

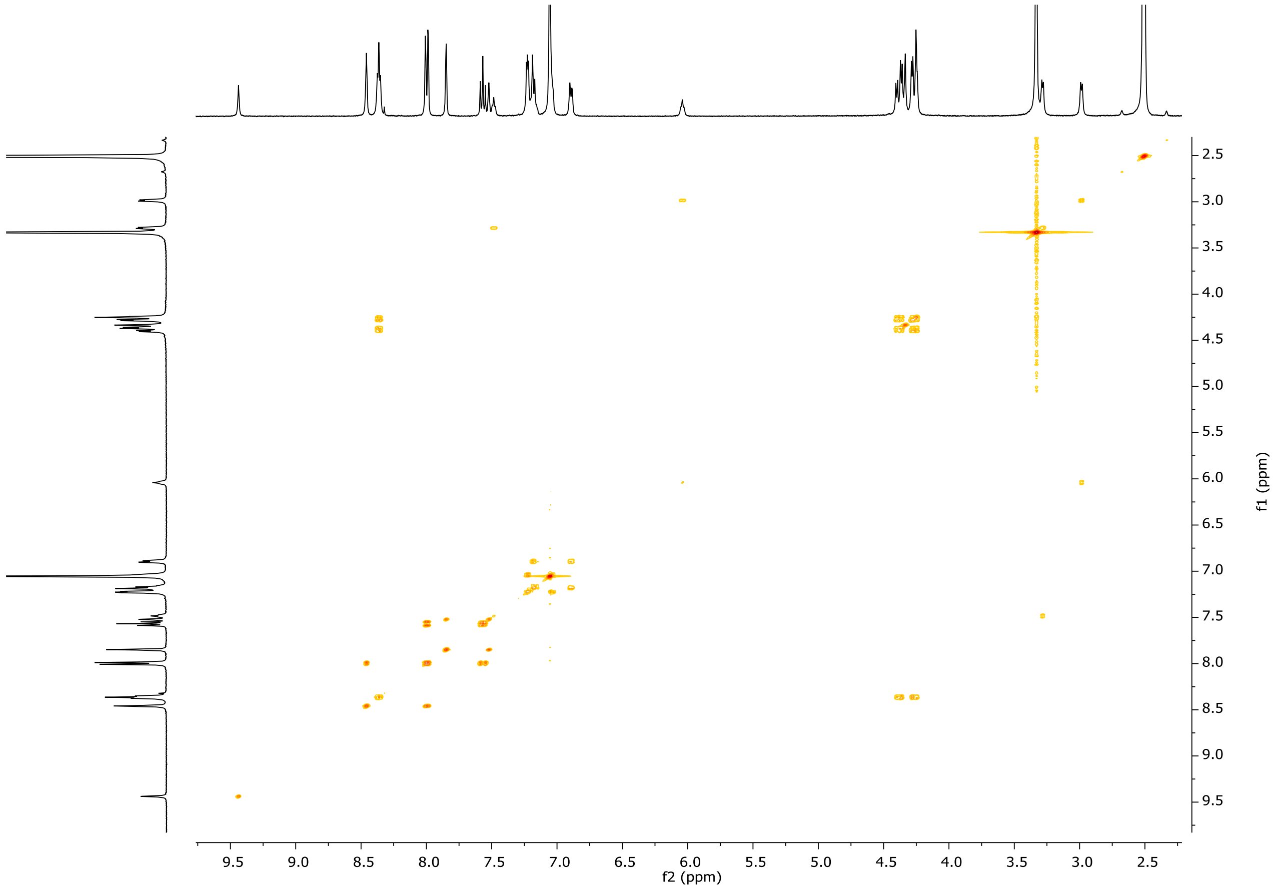

2b (^{13}C NMR, 100 MHz, DMSO-d$_6$, 298 K)

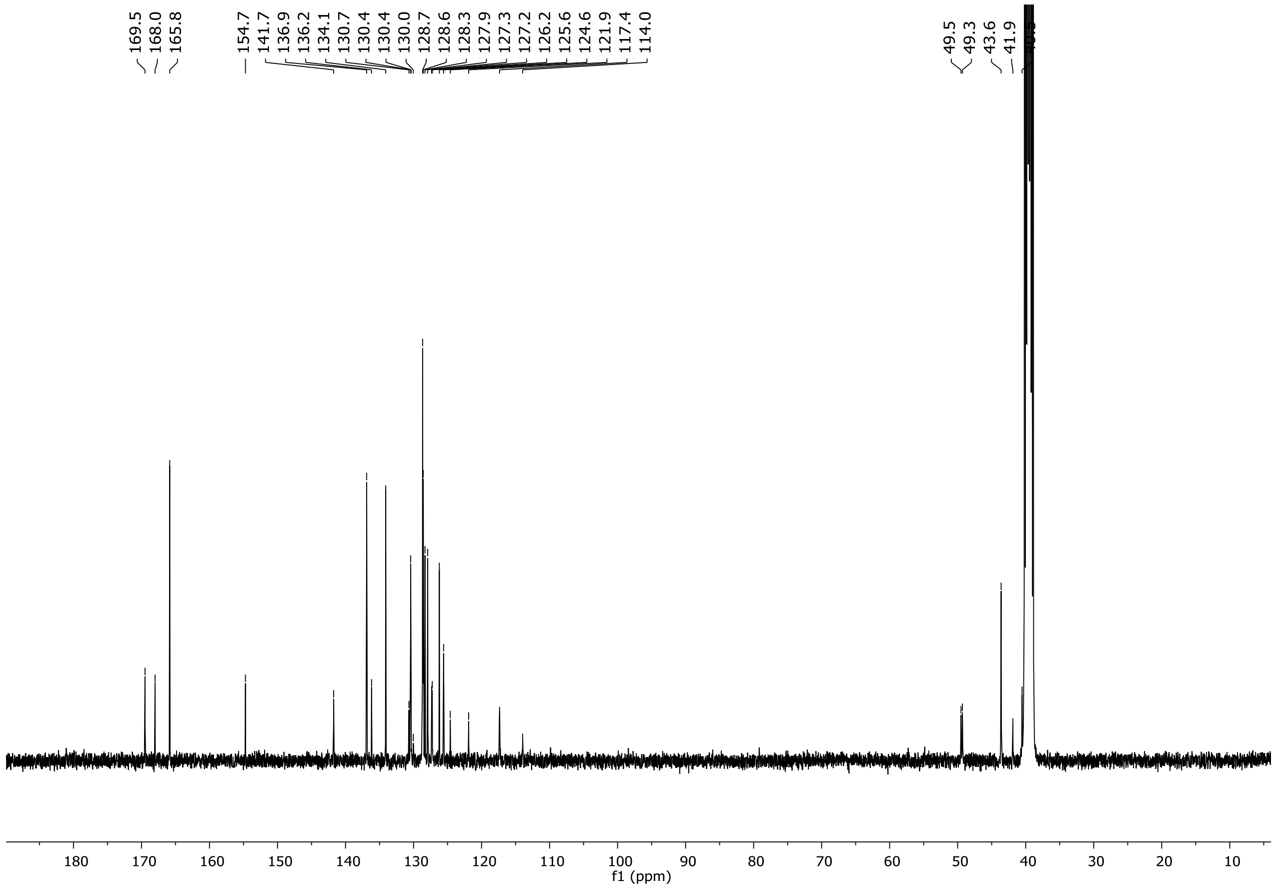

2b (DEPT ^{13}C NMR, 100 MHz, DMSO-d$_6$, 298 K)

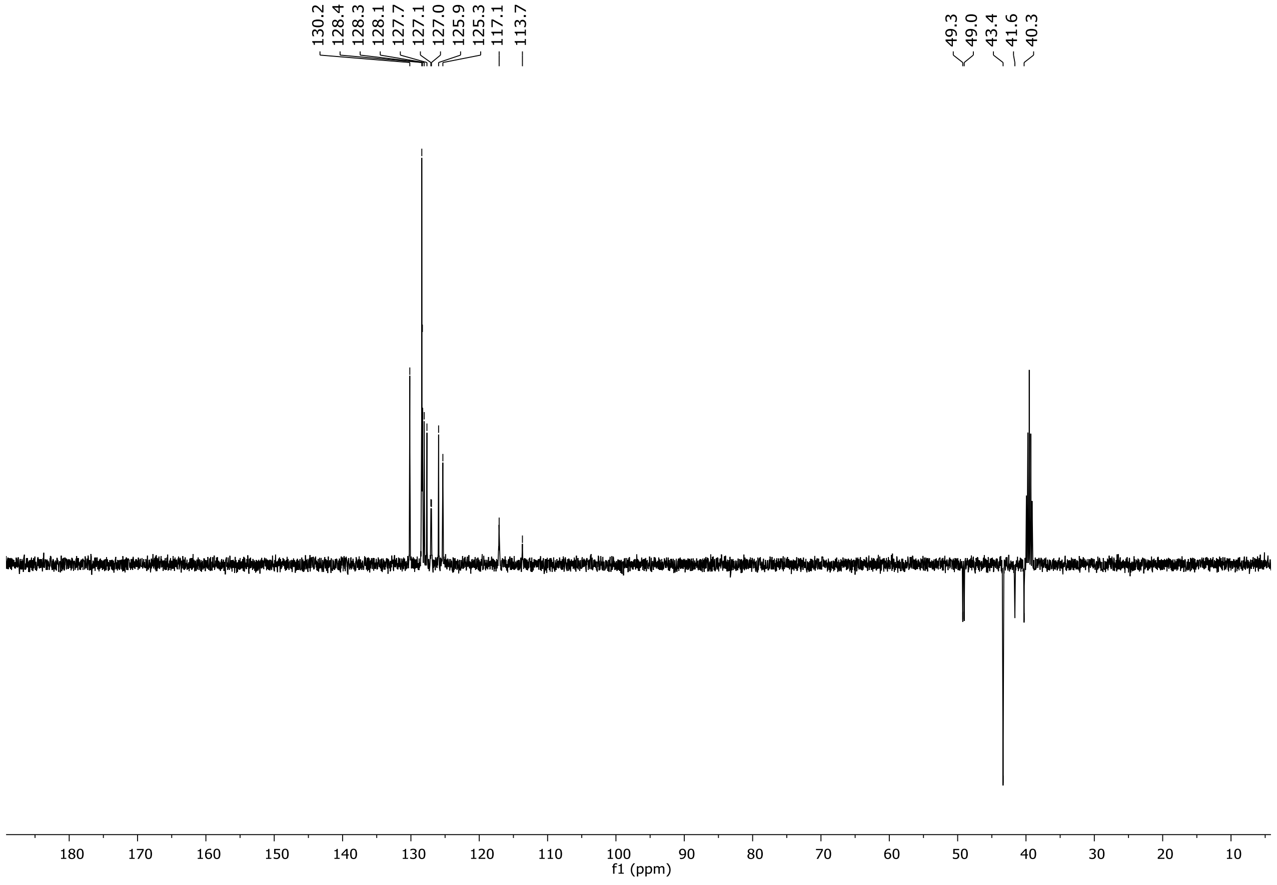

2b (^{19}F NMR, 376 MHz, $CDCl_3$, 298 K)

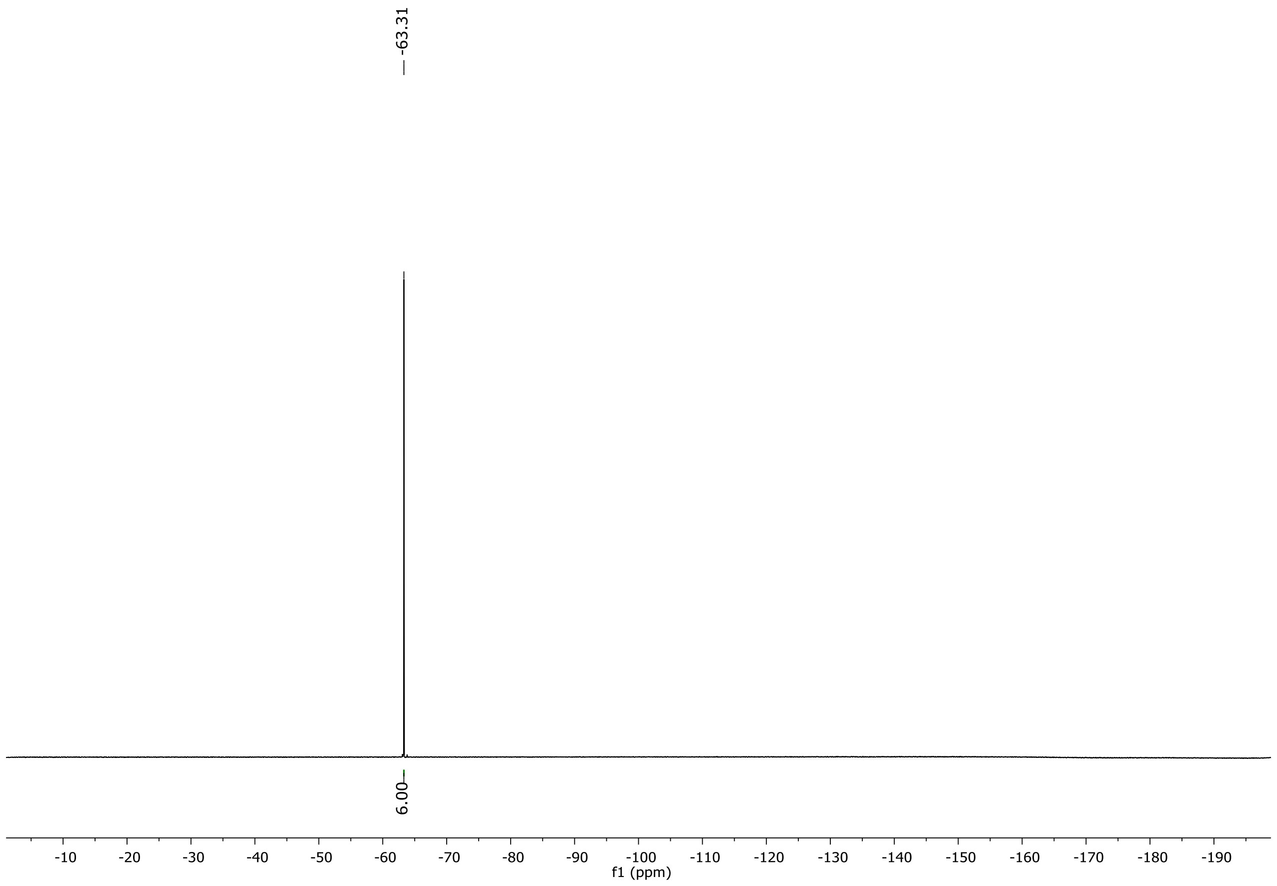

2c (^{1}H NMR, 600 MHz, $CDCl_3$, 298 K)

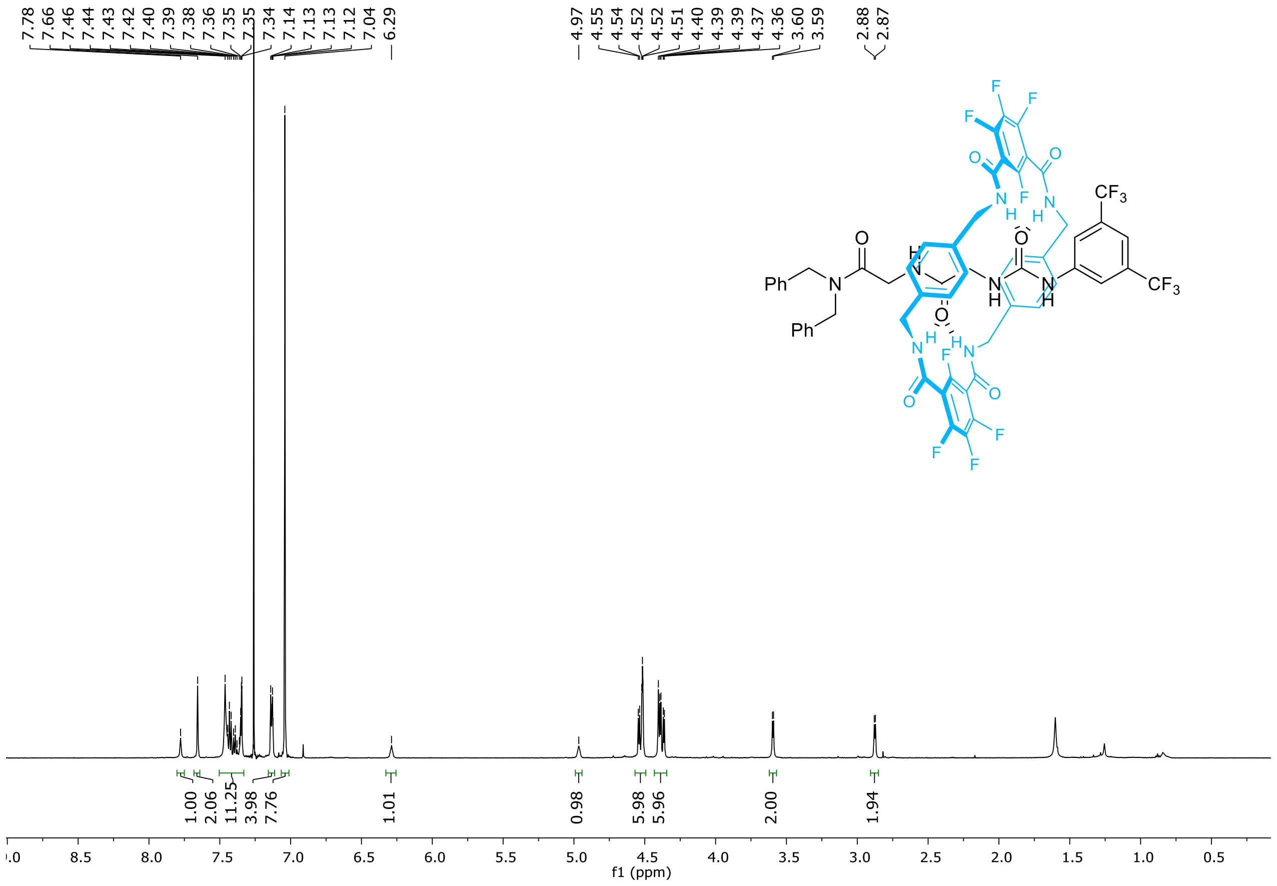

2c (COSY ^{1}H-^{1}H NMR, 600 MHz, $CDCl_3$, 298 K)

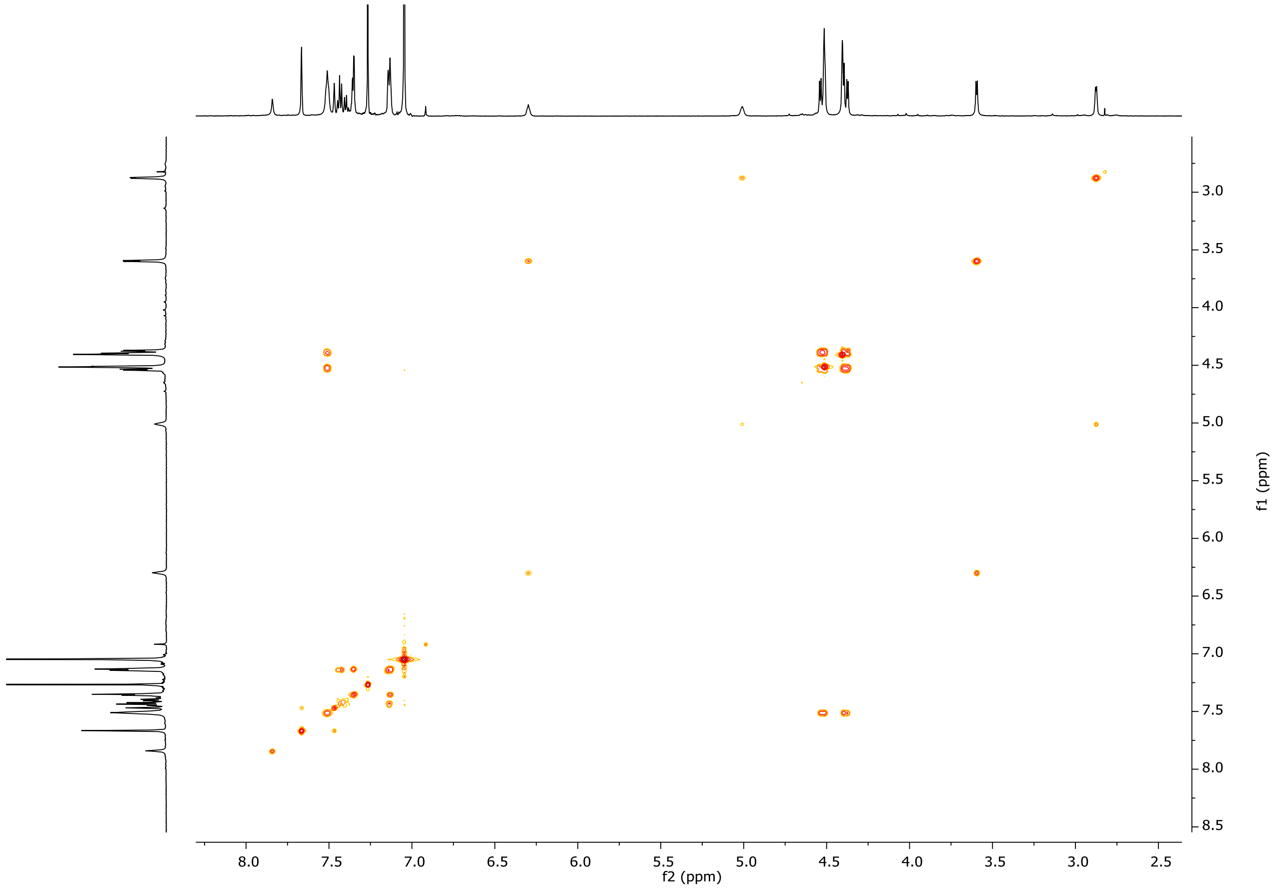

2c (NOESY ^{1}H-^{1}H NMR, 400 MHz, $CDCl_3$, 298 K)

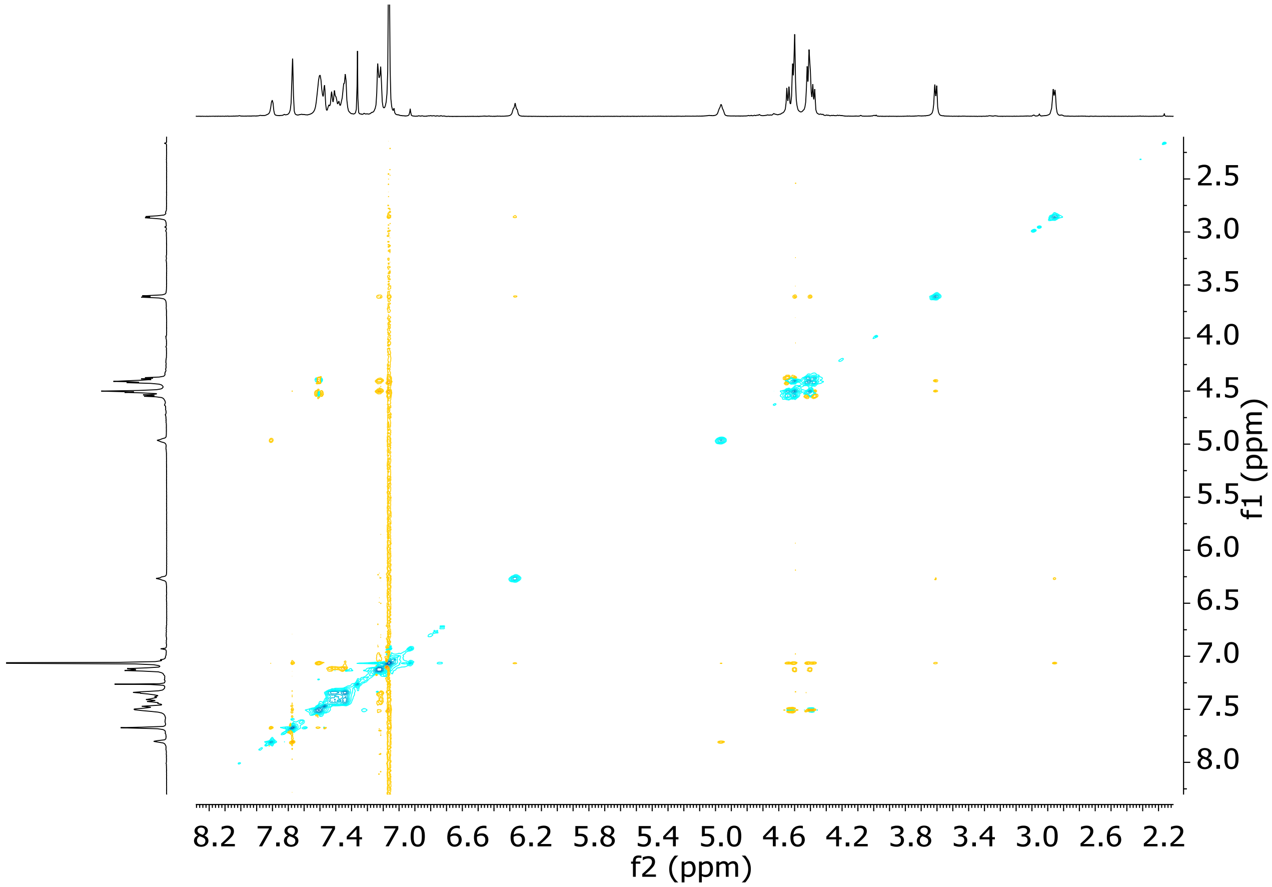

2c (^{1}H NMR, 400 MHz, DMSO-d_6, 298 K)

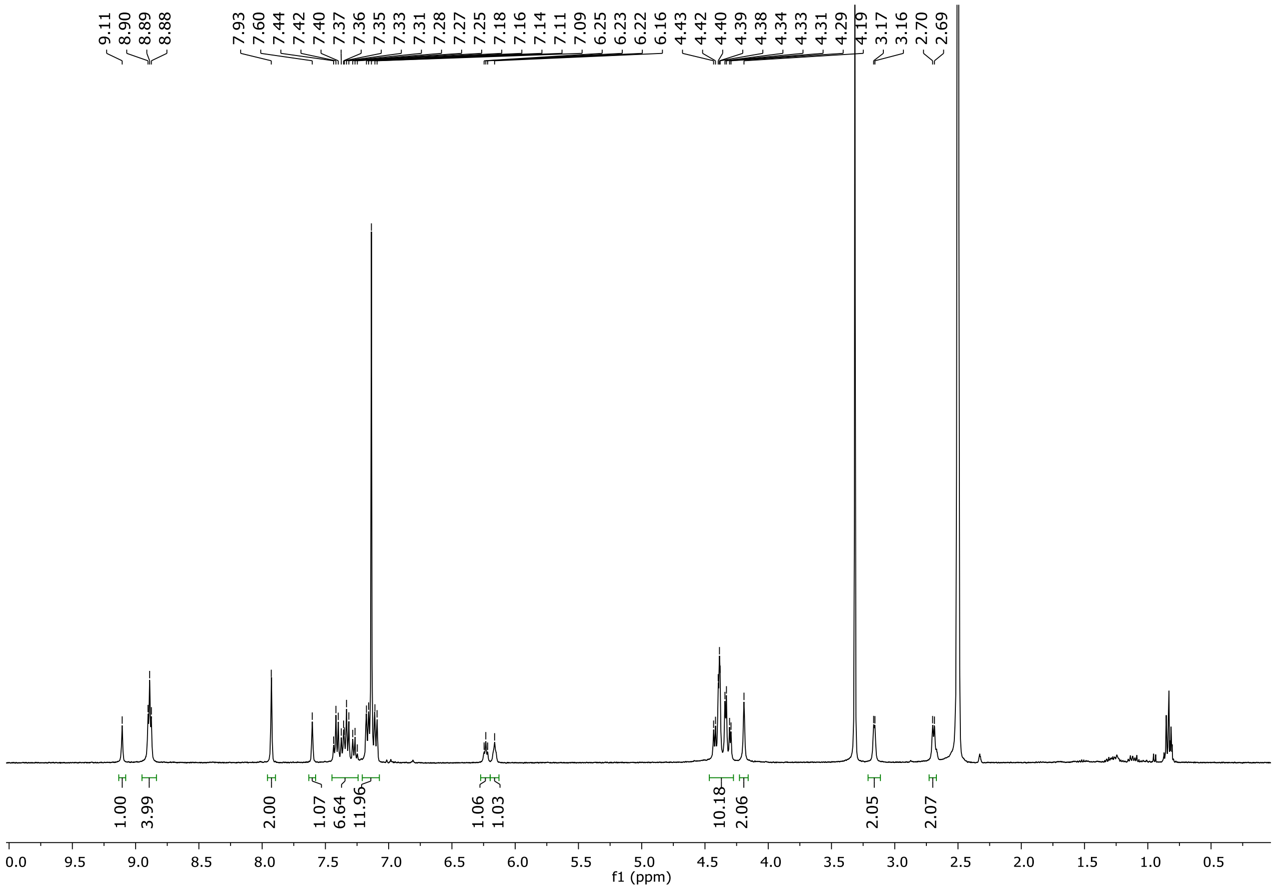

2c (COSY ^1H-^1H NMR, 400 MHz, DMSO-d_6, 298 K)

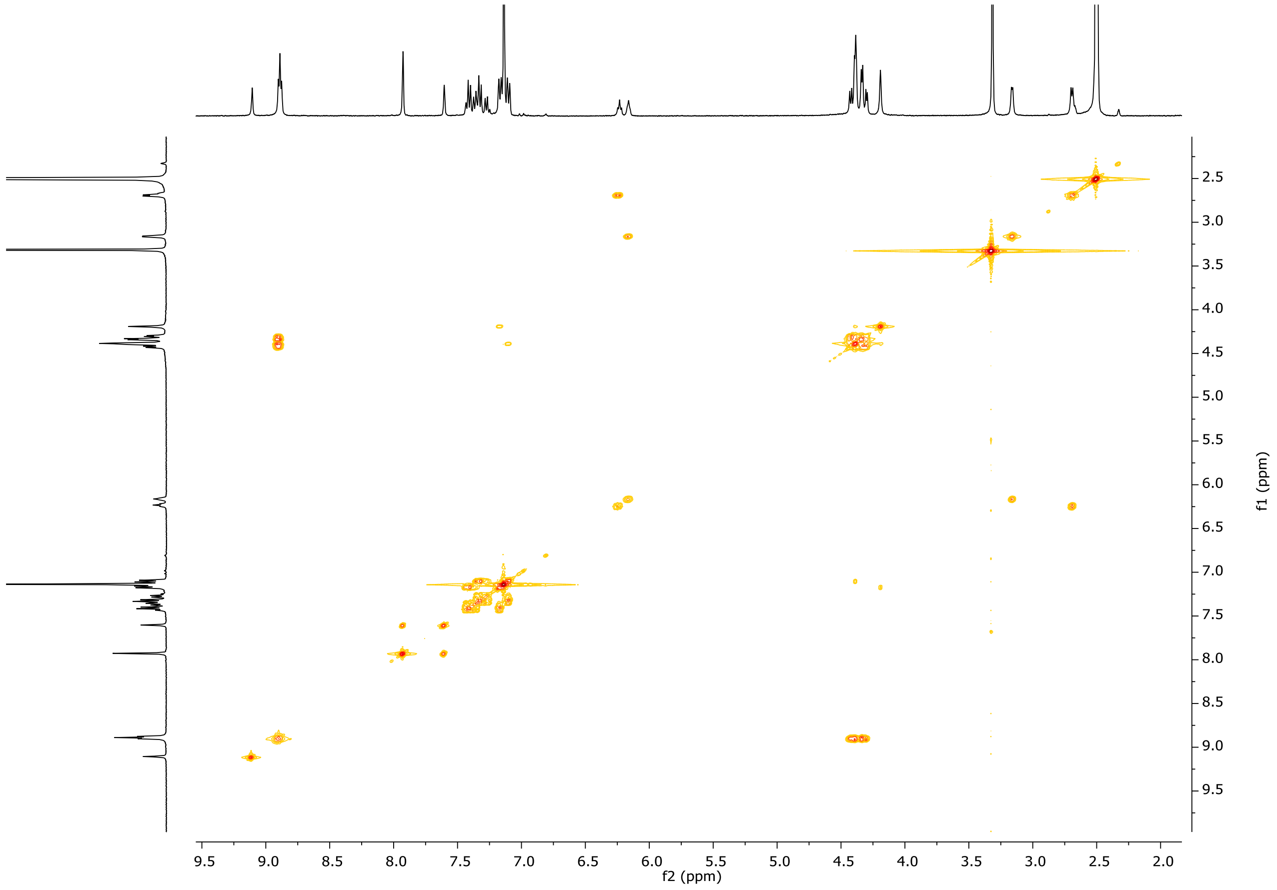

2c (^{13}C NMR, 151 MHz, $CDCl_3$, 298 K)

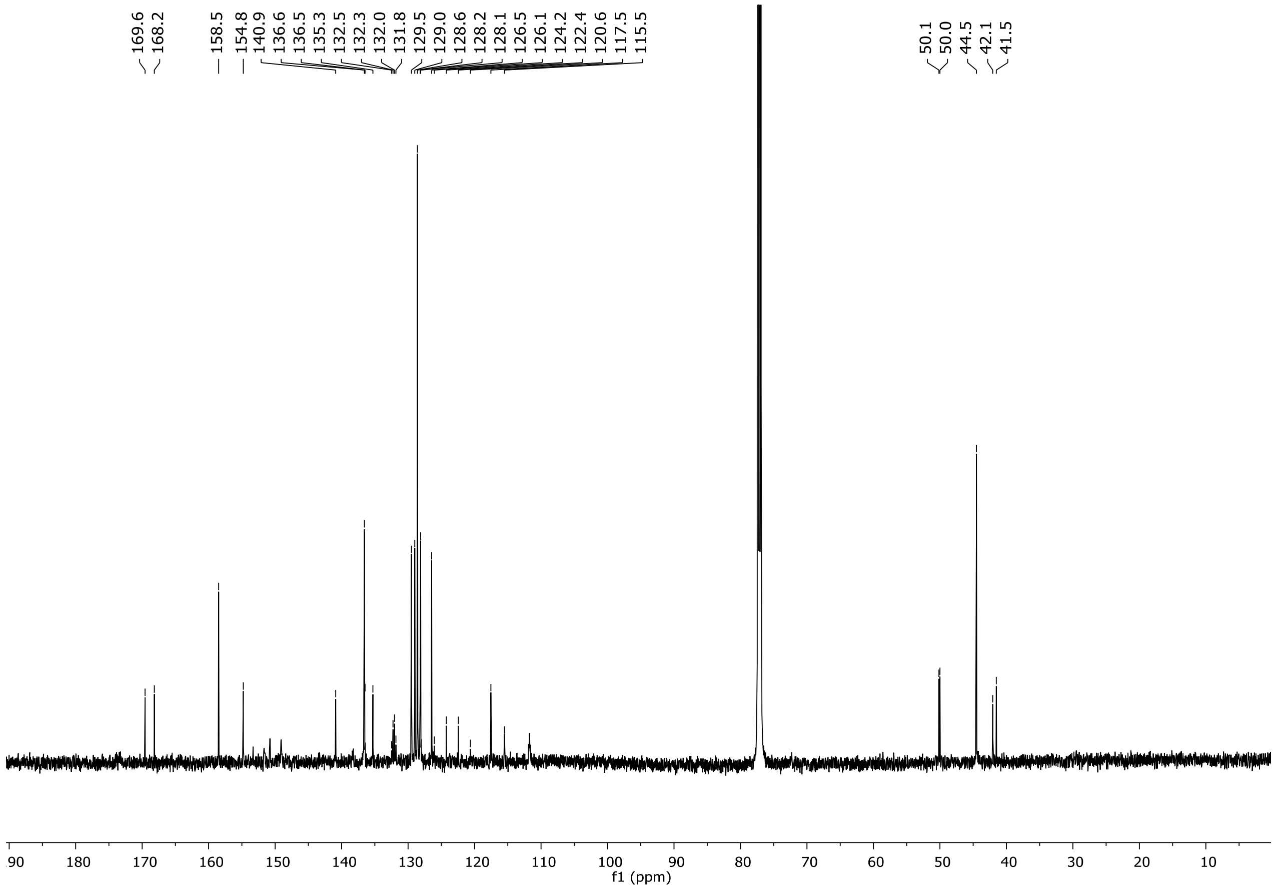

2c (DEPT ^{13}C NMR, 151 MHz, $CDCl_3$, 298 K)

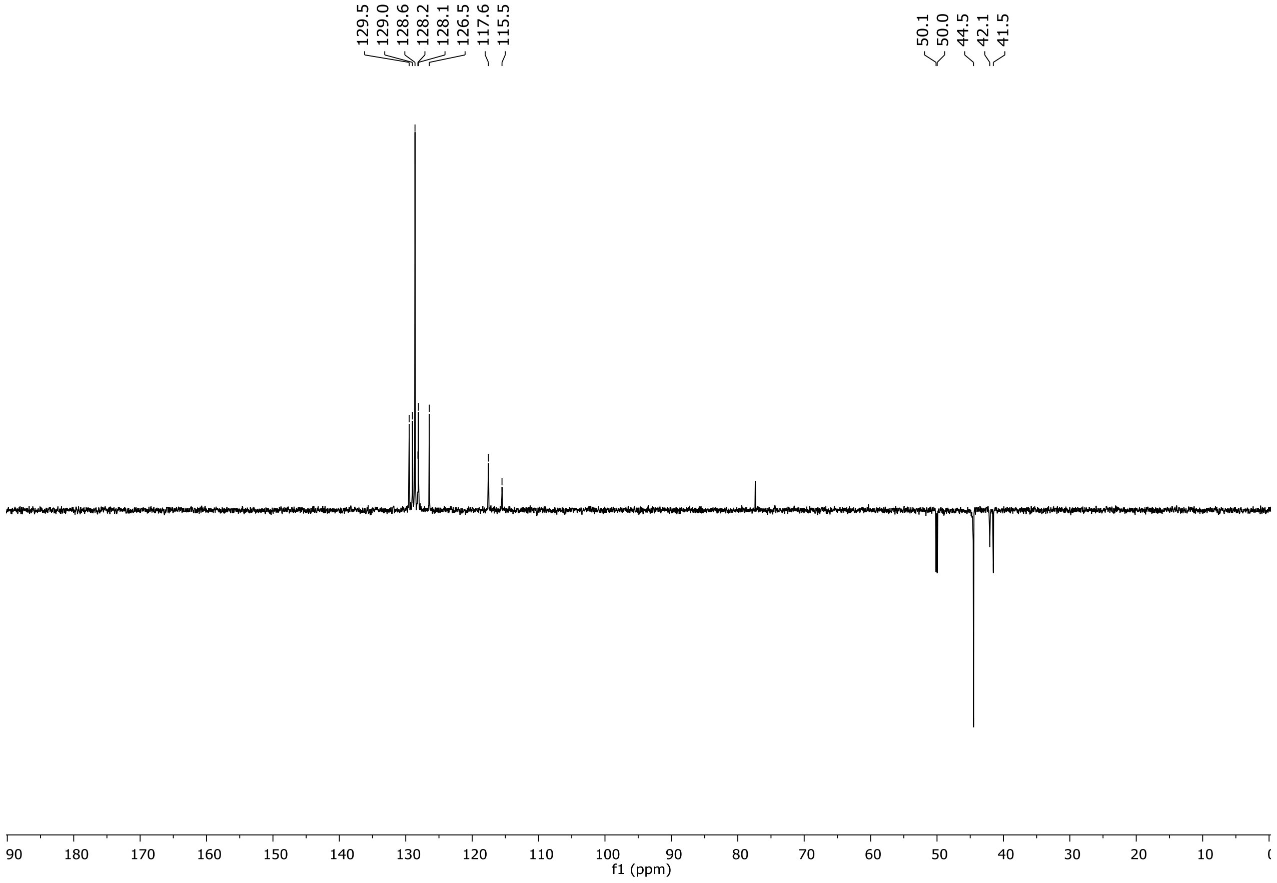

2c (^{19}F NMR, 376 MHz, $CDCl_3$, 298 K)

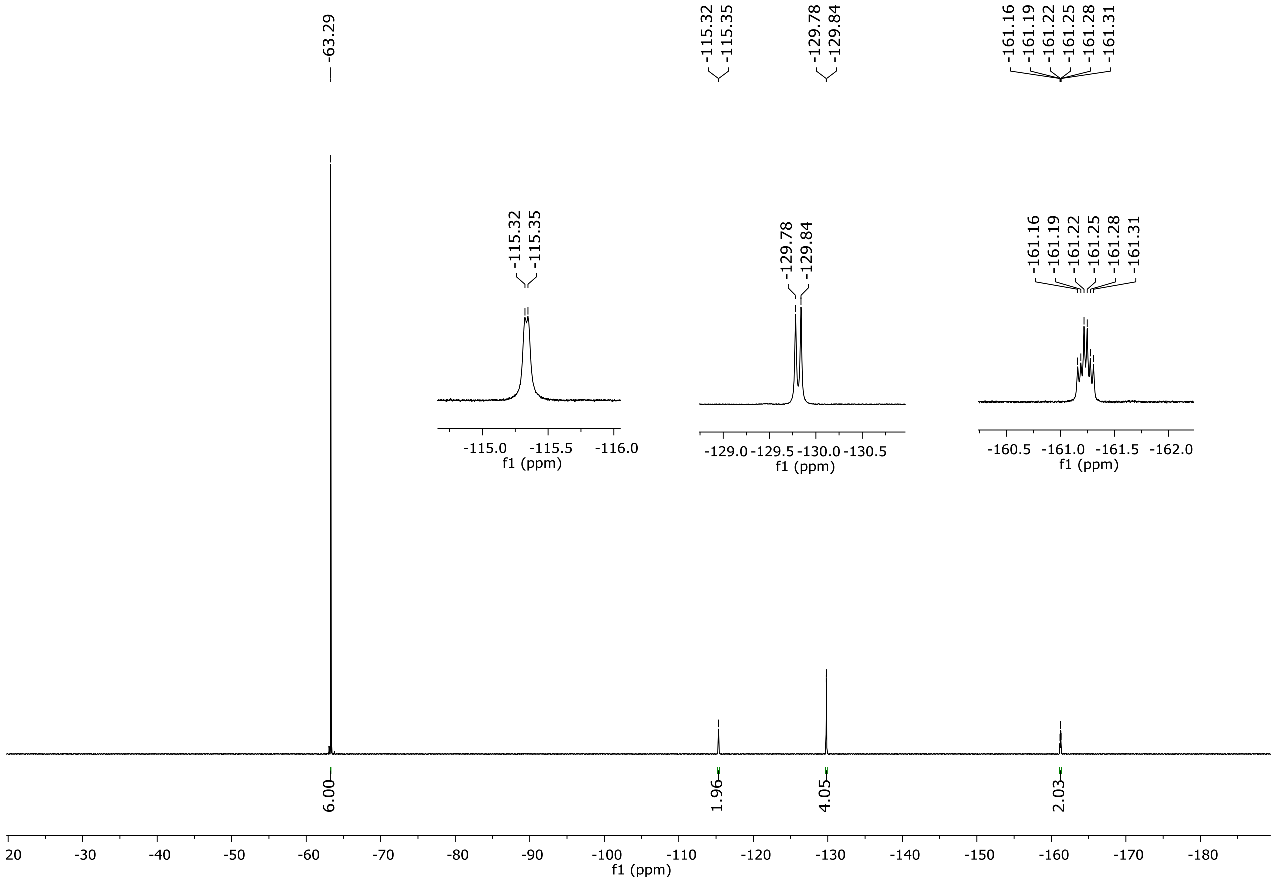

Mac (^{1}H NMR, 400 MHz, DMSO-d$_6$, 298 K)

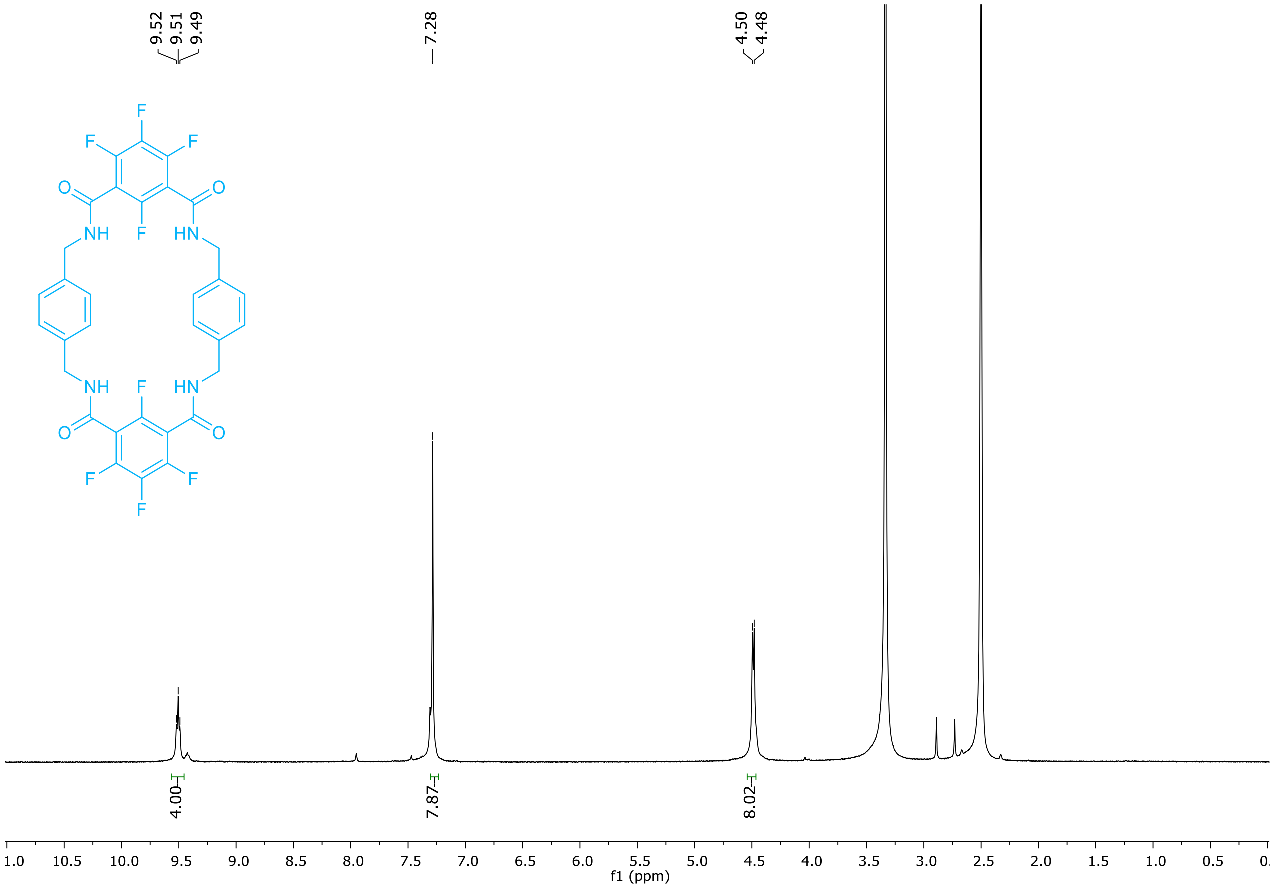

Mac (COSY ^{1}H-^{1}H NMR, 400 MHz, DMSO-d$_6$, 298 K)

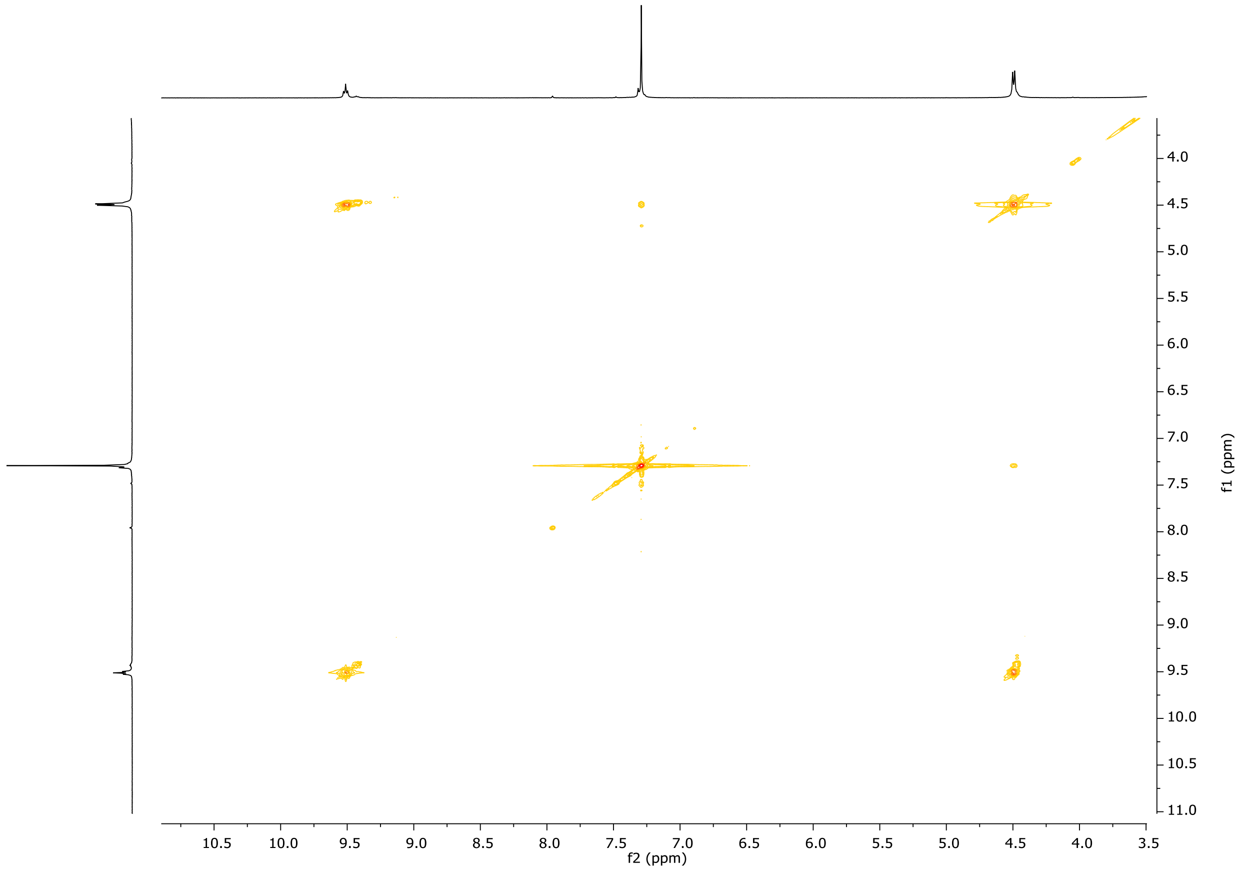

Mac (^{13}C NMR, 100 MHz, DMSO-d_6, 298 K)

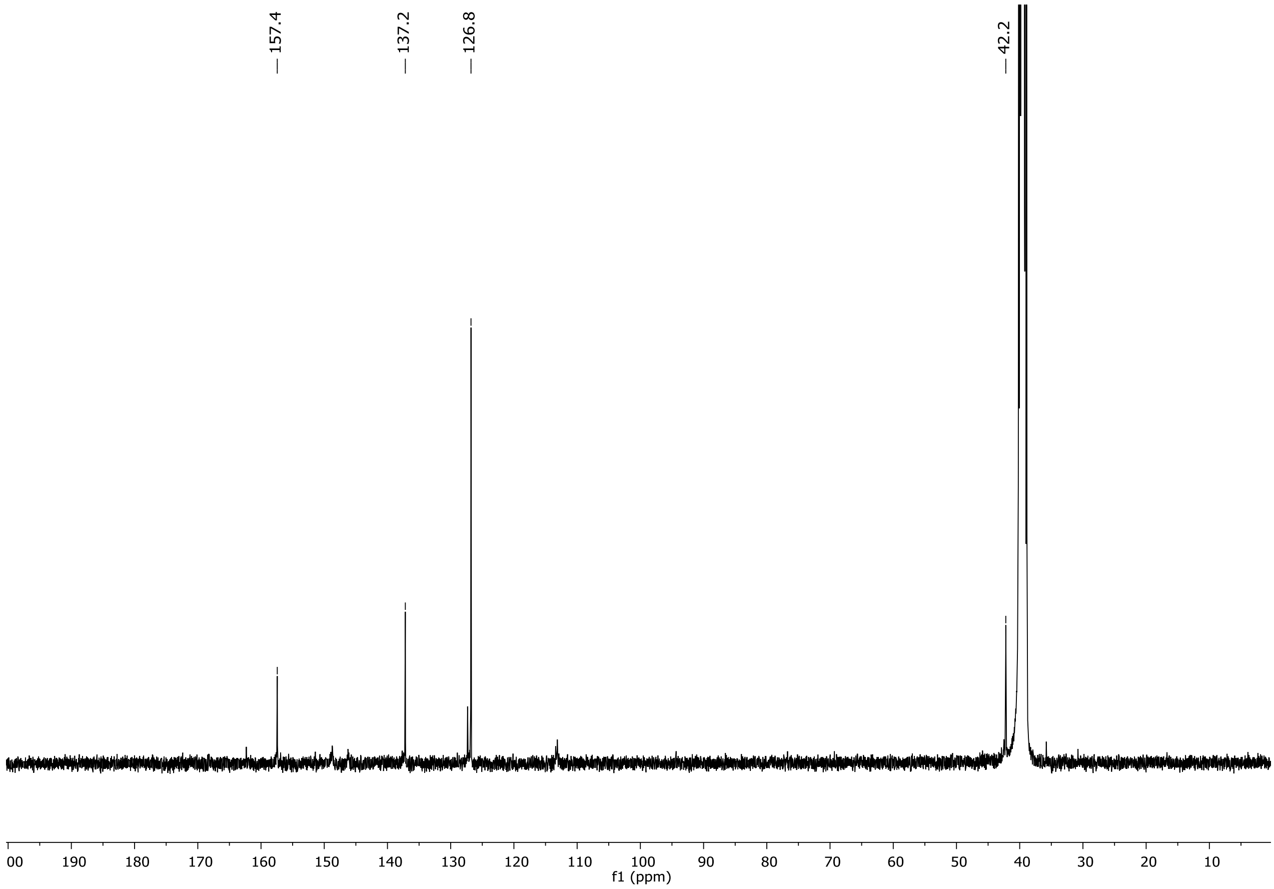

Mac (DEPT ^{13}C NMR, 100 MHz, DMSO-d_6, 298 K)

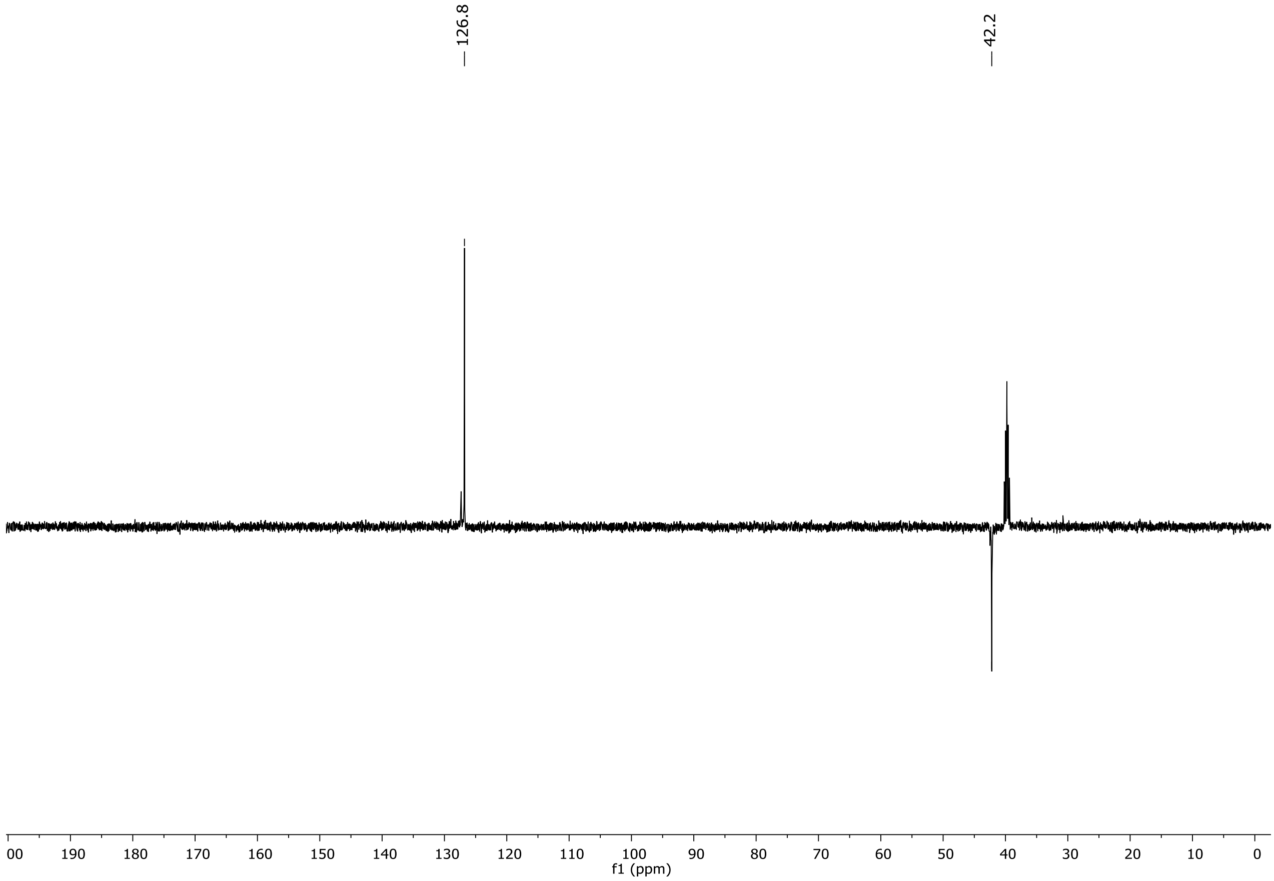

Mac (^{19}F NMR, 376 MHz, DMSO, 298 K)

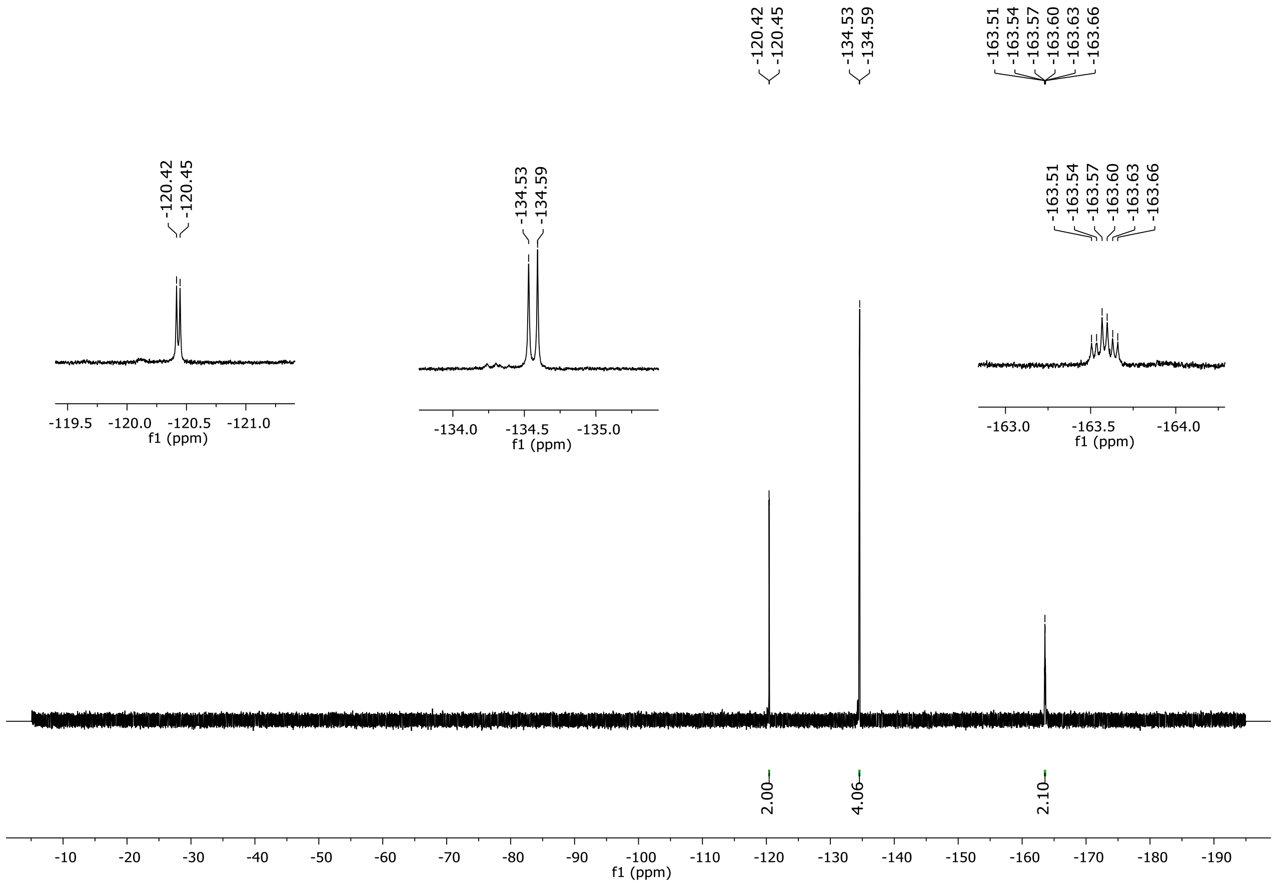

4 (^{1}H NMR, 300 MHz, $CDCl_3$, 298 K)

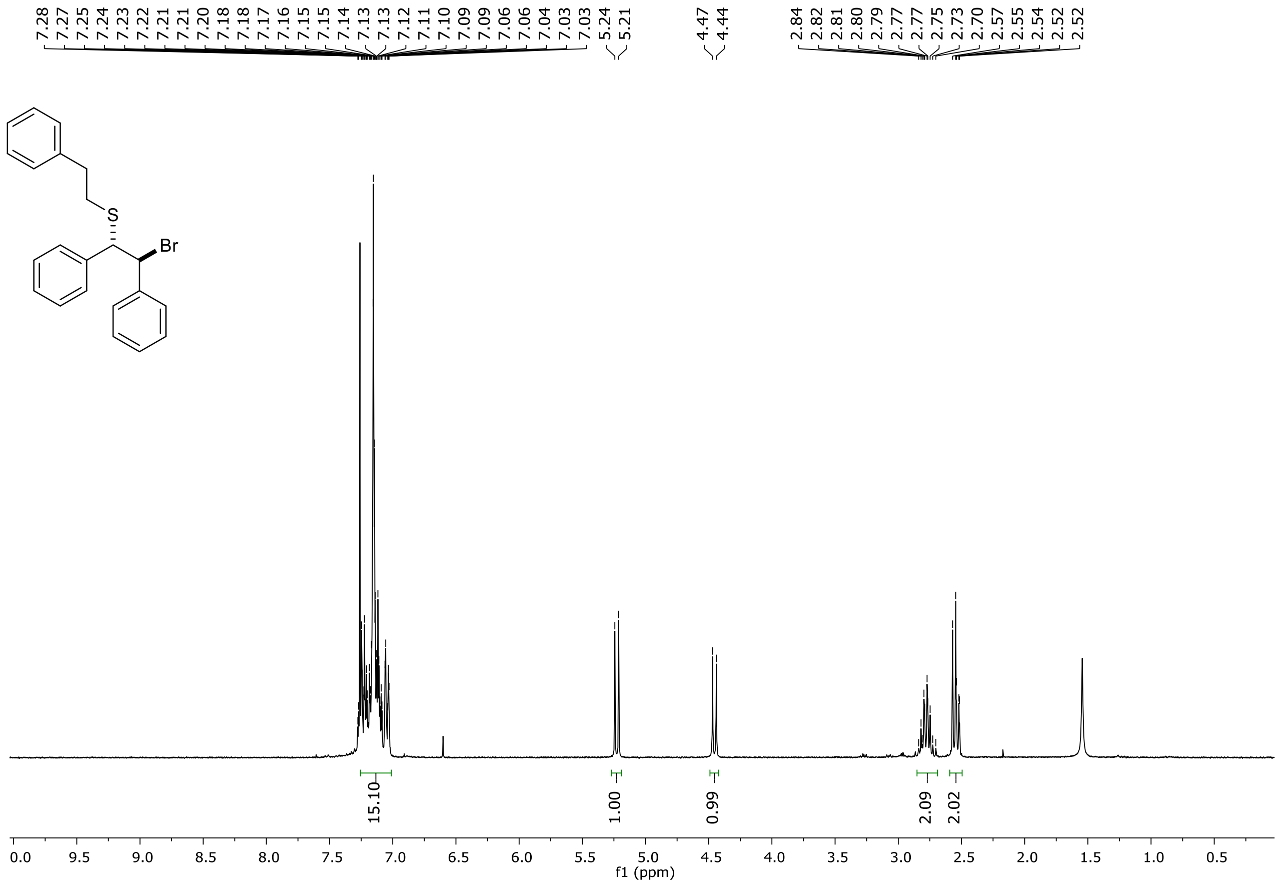

4' (^{1}H NMR, 300 MHz, $CDCl_3$, 298 K)

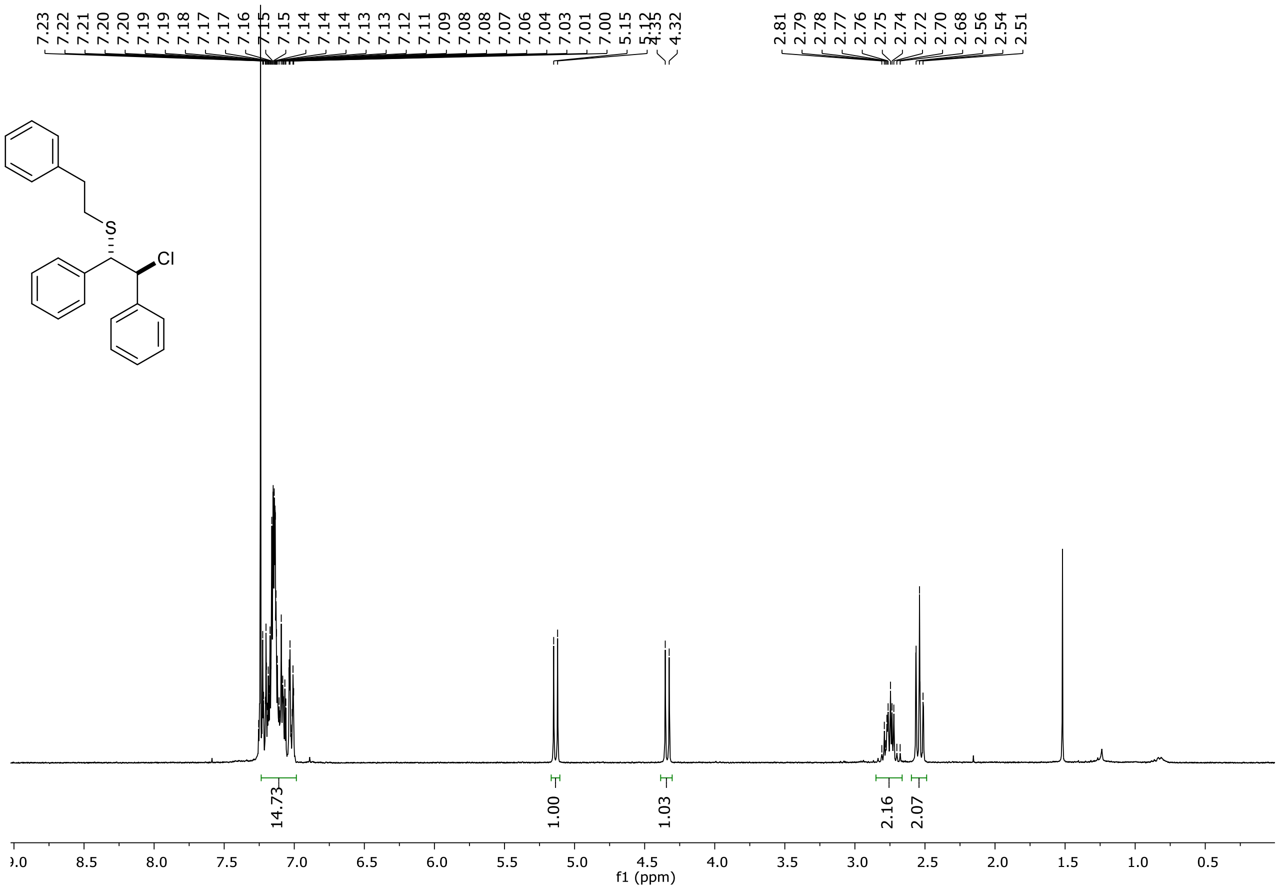

6 (^{1}H NMR, 300 MHz, $CDCl_3$, 298 K)

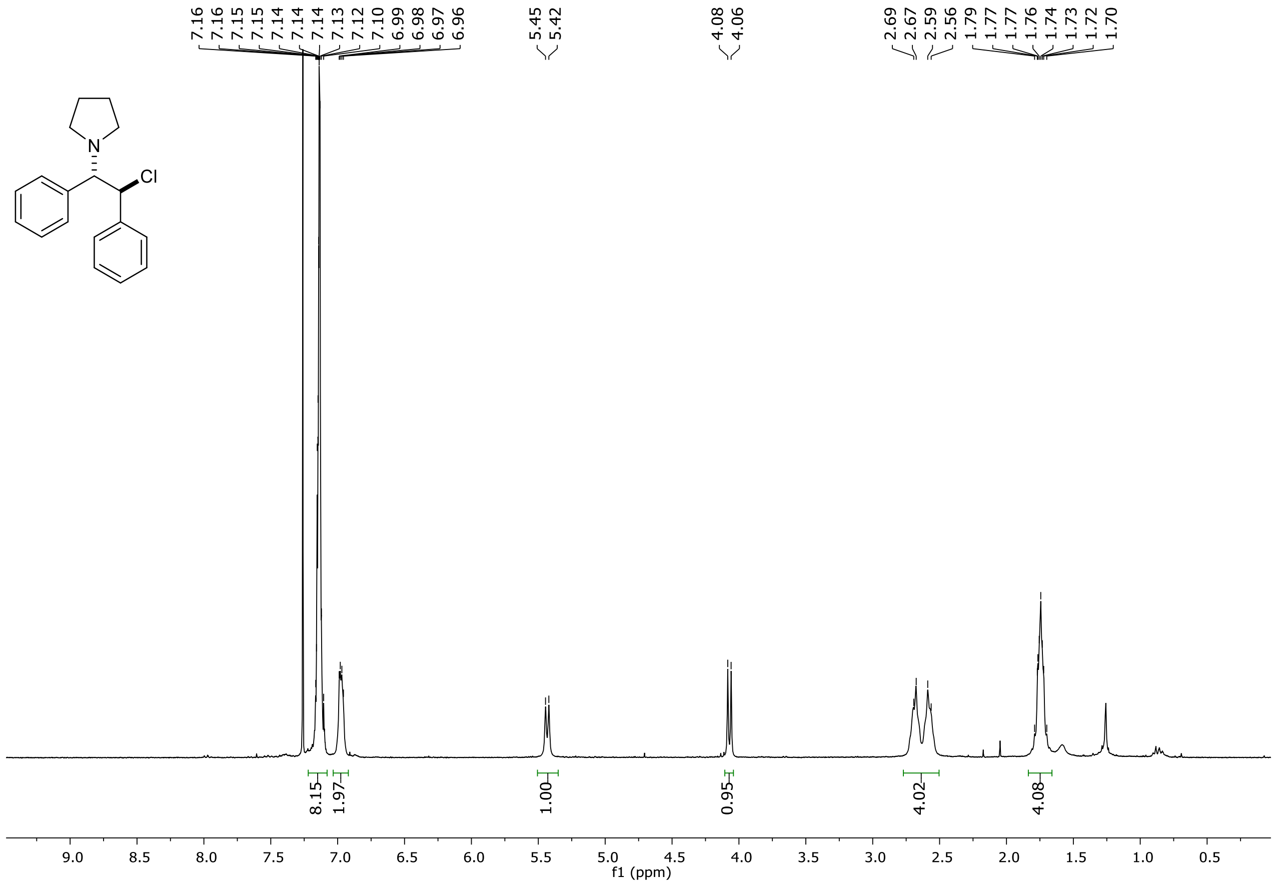

5 (^{1}H NMR, 400 MHz, $CDCl_3$, 298 K)

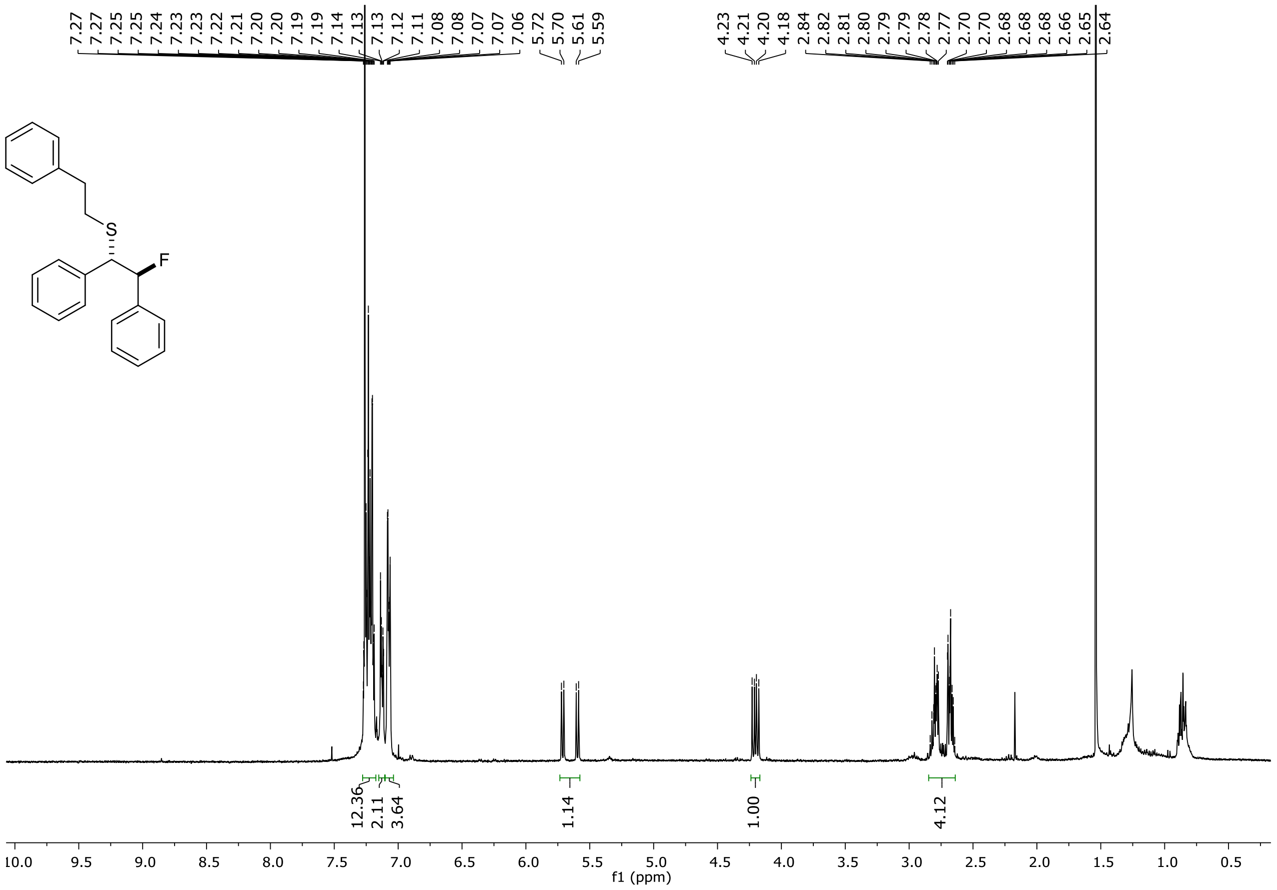

5 (^{19}F NMR, 376 MHz, $CDCl_3$, 298 K)

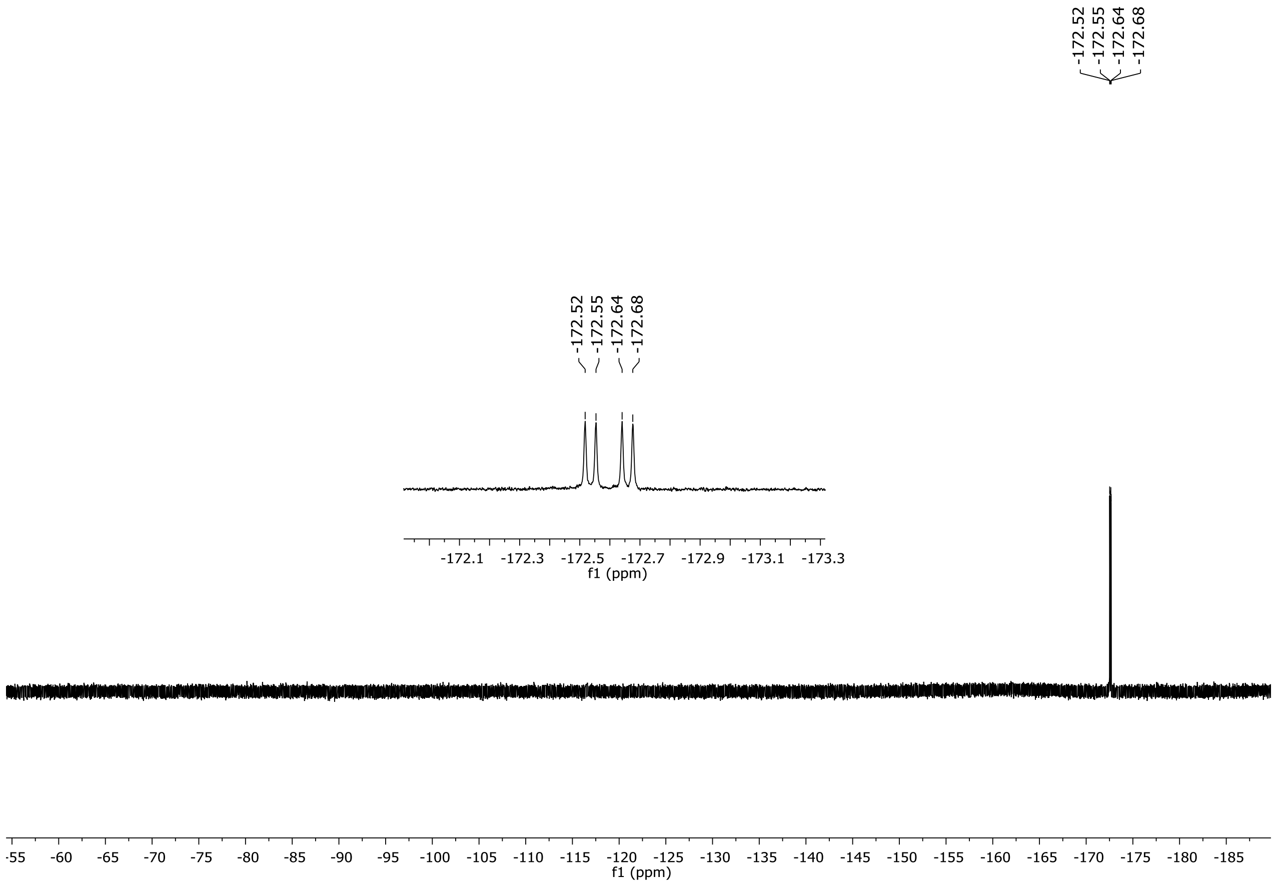

7 (^{1}H NMR, 400 MHz, $CDCl_3$, 298 K)

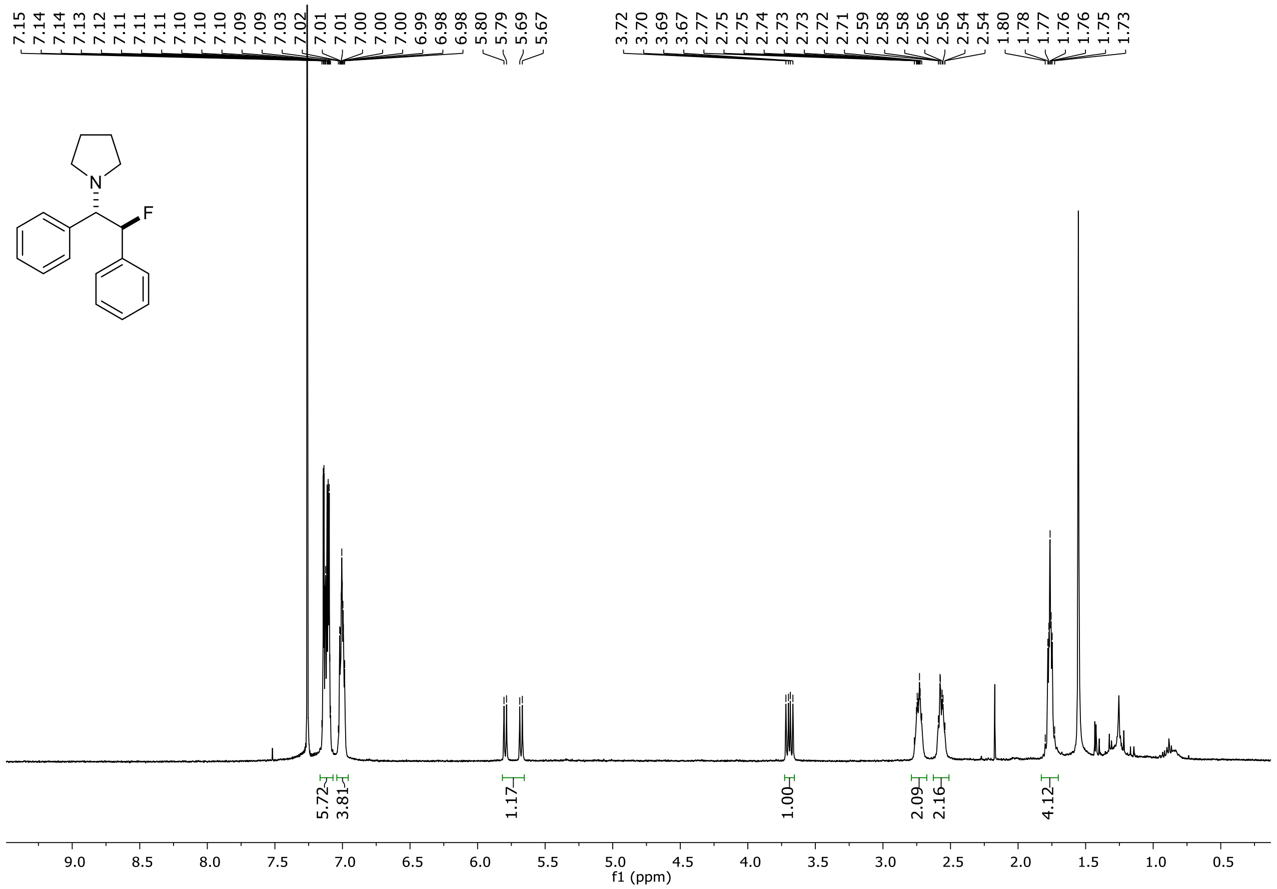

7 (^{19}F NMR, 376 MHz, $CDCl_3$, 298 K)

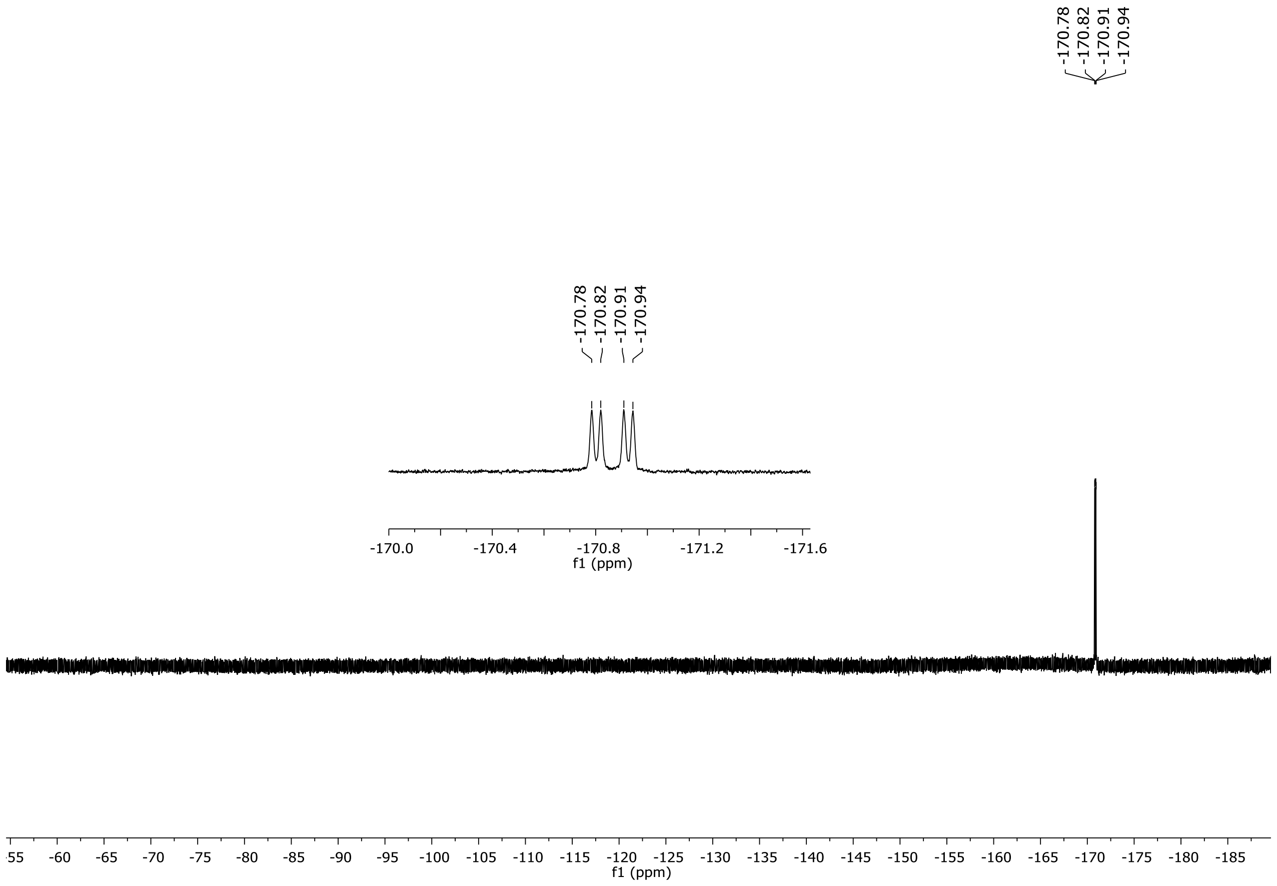

12. References

[1] J. Puigcerver, M. Alajarin, A. Martinez-Cuezva, J. Berna, *Org. Biomol. Chem.*, **2023**, *21*, 9070–9075.
[2] G. Pupo, F. Ibba, D. M. H. Ascough, A. C. Vicini, P. Ricci, K. E. Christensen, L. Pfeifer, J. R. Morphy, J. M. Brown, R. S. Paton, V. Gouverneur, *Science*, **2018**, *360*, 638–642.
[3] G. Pupo, A. C. Vicini, D. M. H. Ascough, F. Ibba, K. E. Christensen, A. L. Thompson, J. M. Brown, R. S. Paton, V. Gouverneur, *J. Am. Chem. Soc.*, **2019**, *141*, 2878–2883.
[4] M. Swain, chemicalize.org. *J. Chem. Inf. Model.*, **2012**, *52*, 613–615.
[5] O. V. Dolomanov, L. J. Bourhis, R. J. Gildea, J. A. K. Howard, H. Puschmann, *J. Appl. Cryst.*, **2009**, *42*, 339–341.
[6] A. Altomare, G. Cascarano, C. Giacovazzo, A. Guagliardi, *J. Appl. Crystallogr.*, **1993**, *26*, 343.
[7] a) G. M. Sheldrick, F2 SHELXL-2014/7: Program for the Solution of Crystal Structures; University of Göttingen: Göttingen, Germany, **2014**; b) G. M. Sheldrick, *Acta Cryst.*, **2015**, *C71*, 3–8.
[8] a) http://supramolecular.org; b) D. Brynn Hibbert, Pall Thordarson, *Chem. Commun.*, **2016**, *52*, 12792–12805; c) P. Thordarson, *Chem. Soc. Rev.*, **2011**, *40*, 1305–1323.
[9] F. Ibba, G. Pupo, A. L. Thompson, J. M. Brown, T. D. W. Claridge, V. Gouverneur, *J. Am. Chem. Soc.*, **2020**, *142*, 46, 19731–19744.
[10] P. Job, *Anal. Chim. Appl.*, **1928**, *9*, 113–203.
[11] S. Grimme, J.G. Brandenburg, C. Bannwarth, A. Hansen, *J. Chem. Phys.*, **2015**, *143*, 054107.
[12] N. Mardirossian, M. Head-Gordon, *Phys. Chem. Chem. Phys.*, **2014**, *16*, 9904–9924.
[13] J. Zheng, X. Xu, D. G. Truhlar. *Theor. Chem. Acc.*, **2011**, *128*, 295–305.
[14] D. Rappoport, F. Furche, *J. Chem. Phys.*, **2010**, *133*, 134105.
[15] A. V. Marenich, C. J. Cramer, D. G. Truhlar, *J. Phys. Chem. B.*, **2009**, *113*, 6378–6396.
[16] a) Y. Zhao, D. G. Truhlar, *Theor. Chem. Acc.*, **2008**, *120*, 215-241. b) E. Papajak, J. Zheng, H. R. Leverentz, D. G. Truhlar, *J. Chem. Theory and Comput.*, **2011**, *7*, 3027–3034.
[17] F. Neese, *WIREs Comput. Mol. Sci.*, **2022**; 12:e1606.
[18] Gaussian 16. Revision C.01. M. J. Frisch, G. W. Trucks, H. B. Schlegel, G. E. Scuseria et al. Gaussian. Inc., Wallingford CT, **2016**.
